# MR-AIV reveals in vivo brain-wide fluid flow with physics-informed AI

**DOI:** 10.1126/sciadv.aeb0404

**Published:** 2026-05-27

**Authors:** Juan Diego Toscano, Yisen Guo, Zhibo Wang, Mohammad Vaezi, Yuki Mori, George Em Karniadakis, Kimberly A. S. Boster, Douglas H. Kelley

**Affiliations:** ^1^ Division of Applied Mathematics, Brown University, Providence, RI 02912, USA.; ^2^ Department of Mechanical Engineering, University of Rochester, Rochester, NY 14627, USA.; ^3^ School of Engineering, Brown University, Providence, RI 02912, USA.; ^4^ Center for Translational Neuromedicine, University of Copenhagen, Copenhagen 2200, Denmark.

## Abstract

The circulation of cerebrospinal and interstitial fluid plays a vital role in clearing metabolic waste from the brain, and its disruption has been linked to neurological disorders. However, directly measuring brain-wide fluid transport, especially in the deep brain, has remained elusive. Here, we introduce magnetic resonance artificial intelligence velocimetry (MR-AIV), a framework featuring a specialized physics-informed architecture and optimization method that reconstructs three-dimensional fluid velocity fields from dynamic contrast-enhanced magnetic resonance imaging (DCE-MRI). MR-AIV unveils brain-wide velocity maps while providing estimates of tissue permeability and pressure fields, quantities inaccessible to other methods. Applied to the brain, MR-AIV reveals a functional landscape of interstitial and perivascular flow, quantitatively distinguishing slow diffusion-driven transport [∼0.1 micrometers per second (μm/s)] from rapid advective flow (∼3 μm/s). This approach enables new investigations into brain clearance mechanisms and fluid dynamics in health and disease, with broad potential applications to other porous medium systems, from geophysics to tissue mechanics.

## Teaser

Physics-informed AI reveals brain fluid flow from MRI, key to understanding brain health and neurological diseases.

## INTRODUCTION

Fluids permeate the brain. Their circulation clears metabolic wastes whose accumulation is linked to diseases like Alzheimer’s and is altered in pathological conditions like stroke (*1*–*5*). Mapping and quantifying the flow of cerebrospinal fluid (CSF) in large spaces and the putative flow of interstitial fluid (ISF) in small extracellular spaces (ECSs) is crucial to understanding the function, failure, and potential rehabilitation of the brain’s waste removal, or “glymphatic,” system. Yet, direct measurement of the brain-wide fluid movement has been impossible. This inability to quantify the fluid dynamics governing waste clearance has fundamentally limited our understanding of diseases like Alzheimer’s.

Current techniques for imaging brain flows are often invasive, superficial, or lack spatial resolution. Tracking particles imaged in vivo with two-photon microscopy (*4*, *6*–*8*) yields high-fidelity velocity measurements [especially when supplemented with artificial intelligence velocimetry (AIV); (*9*, *10*)] but can be done only in regions near the brain surface, such as pial (surface) perivascular spaces (PVSs) of mice. Flow velocities have been estimated by tracking tracer dye fronts in brain-wide imaging in mice (*4*, *11*, *12*), but front tracking neglects diffusion and out-of-plane flow. Both particle tracking and front tracking require invasive surgeries, like cranial windows or scalp removal, ruling out widespread clinical use with human patients. Dynamic contrast-enhanced magnetic resonance imaging (DCE-MRI) requires no surgery and measures signal from an injected tracer, over space and time. DCE-MRI has been used to quantify transport with apparent diffusion coefficients (*13*–*15*), but transport by bulk flow (advection) is a fundamentally different process than diffusion and cannot accurately be described by such coefficients. Data-driven approaches show promise for biological flow modeling (*16*–*19*), but without direct measurements of velocity in the deep brain, data-driven methods cannot be applied.

DCE-MRI has been combined with other methods to estimate velocities from observed tracer concentrations. Flows in rat brains have been mapped using optimal mass transport (OMT) (*20*–*24*), an optimal control approach that accounts for diffusion and noise using a regularization term. However, OMT uses a diffusivity-like constant in the mass transport equation that is heuristically chosen, not stated in terms of physical diffusion coefficients, making it difficult to compare the results with other works. Flows in human brains have been estimated by fitting several different models to DCE-MRI measurements using adjoint methods (*25*). However, neither OMT nor adjoint methods have been validated with synthetic data. Additionally, neither can infer permeability or pressure because those quantities are absent from the governing equations used.

Although DCE-MRI can accurately image an injected tracer as it spreads through the brain, mapping and quantifying the flow that is complicit in that transport is difficult. Calculating the velocity field from tracer motion requires solving an ill-posed inverse problem in which boundary conditions are usually unknown. In vivo measurements are sparse: Particle tracking produces measurements only at particles’ locations; front tracking produces measurements only at fronts’ locations; and MRI has spatial resolution coarser than the size of key structures like PVSs, causing partial-volume effects. Measurement noise undermines many methods for solving inverse problems. Efflux routes are not well-known (*26*). Velocity magnitudes in the brain span several orders of magnitude, from ∼10^1^ μm/s measured in PVSs (*6*) to perhaps ∼10^−2^ μm/s or less expected in ECSs (*27*). Tissue properties like permeability, which strongly affect flows, are also likely to span wide ranges. Last, the physics that governs brain flow is not well-known. Some open spaces harbor viscous flow governed by the Stokes equation (*7*). Most of the brain, however, is usually modeled as a porous medium, with momentum governed by Darcy’s law (*28*) or the Darcy-Brinkman equation (*29*). Some studies have modeled tissue as poroelastic (*30*, *31*). Physics involving fractional derivatives may also be applicable (*32*).

To address these challenges, we developed magnetic resonance artificial intelligence velocimetry (MR-AIV), a physics-informed machine learning (PIML) framework that infers three-dimensional (3D) velocity, pressure, and permeability fields solely from tracer concentration data. MR-AIV fundamentally advances the capabilities of standard PIML (*33*) to tackle ill-posed problems from noisy multiscale experimental data through three major innovations.

First, unlike standard single-network models (*9*, *10*, *34*–*36*), MR-AIV uses a modular architecture with four specialized networks to independently model pressure, permeability, the clean concentration signal, and its associated noise. This structure enables Darcy’s law and the steady-state assumptions to be incorporated exactly, thereby reducing the number of stabilizing constraints and simplifying training. Additionally, encoding Darcy’s law reduces the number of variables to model, partially mitigating the problem’s ill-posedness. Notably, having a dedicated network for permeability is crucial for tackling the multiscale nature of the velocities. Because permeability in the brain spans several orders of magnitude, this modular component acts as an adaptive map, enabling the model to accurately capture the sharp transitions between slow interstitial and rapid perivascular flow.

Second, it directly confronts noisy experimental data by modeling the measurement error as Gaussian noise. This assumption enables the use of a negative log-likelihood objective, allowing one of the network modules to explicitly learn the space- and time-dependent noise, while another module learns the clean, denoised concentration signal. The governing physical laws are then constrained only on this clean signal, not the raw data, preventing the model from fitting to noise and significantly improving performance and robustness.

Last, we introduce time-dependent residual-based attention (TD-RBA), an optimization method that addresses the challenge of physical residuals that vary by orders of magnitude over time. This method guides the optimizer using physics-based attention, effectively acting as a pointwise adaptive learning rate that is informed by the governing equations, ensuring that all phases of the tracer’s transport contribute meaningfully to the final solution.

These three innovations were developed specifically to address the challenges inherent in inferring velocity from measurements of tracer injected in a brain, but they represent important improvements to PIML that could be applied to any flow through porous medium governed by Darcy’s Law, including flows in geophysics, tissue mechanics, thrombosis, and biomechanics.

To test MR-AIV, we simulated the spread of tracer injected into the cisterna magna of a mouse, a method common in experiments, using a realistic geometry of a mouse brain with permeabilities spanning four orders of magnitude, a challenging feature that we expect in real brains. An MR-AIV model, trained only on the simulated concentration, accurately reproduced not only the concentration but also the underlying velocity field, which was not used for training.

Model accuracy depended little on the initial guess for the permeability of brain tissue, although permeability varies by orders of magnitude across anatomical regions. Quantifying model uncertainty, we found that high uncertainty, slow flow, and high error were strongly correlated. Next, we applied MR-AIV to DCE-MRI measurements of in vivo cisterna magna injections in five wild-type mice. We inferred 3D velocities throughout the brain, including deep regions inaccessible to optical imaging.

MR-AIV offers a data-driven framework for modeling brain fluid transport without requiring direct velocity measurements, taking a step toward comprehensive, noninvasive characterization of glymphatic flow and its potential alterations in aging and neurological diseases. Although, in this work, we demonstrate MR-AIV on mouse brains, it could potentially be applied to human brains because DCE-MRI, which is already used in clinical settings, is noninvasive. Our results demonstrate that this method can reveal physiologically plausible transport patterns in vivo, providing new tools for studying CSF dynamics and potentially informing diagnosis and treatment of neurodegenerative disorders. Because the MR-AIV framework is generalizable to other imaging modalities, organ systems, and even nonbiological flows, it has wide-reaching implications beyond neuroscience.

## RESULTS

### MR-AIV reconstructs velocity fields from concentration observations

**Fig. 1.**
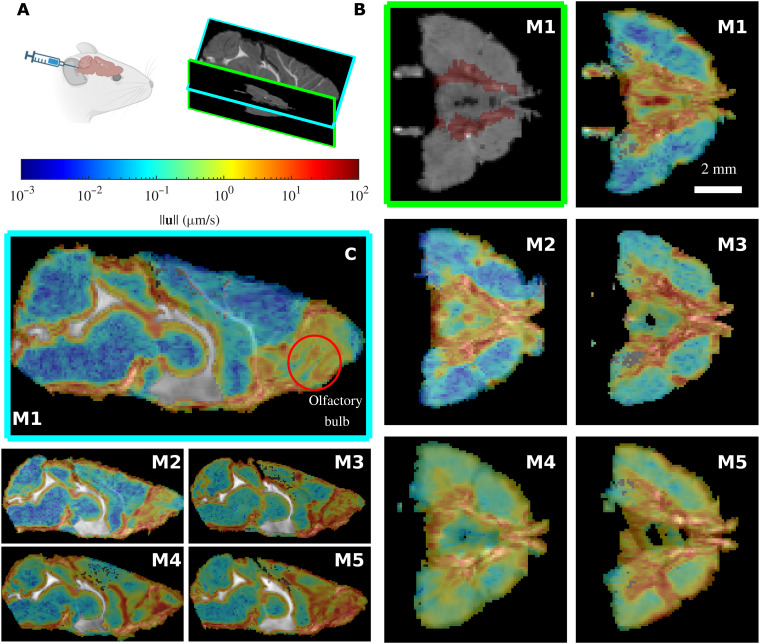
The MR-AIV inferred velocity magnitude ‖u‖ is similar across mice. (**A**) Gadobutrol is infused into the cisterna magna of five mice (M1 to M5), and the tracer movement is recorded via DCE-MRI. (**B**) The Circle of Willis (location marked in red on the M1 structural image at top left) can be seen in the transverse plane. Flow is consistently fast near the Circle of Willis and the olfactory bulb, which can be observed in the midsagittal plane. Velocity fields are overlaid on grayscale structural MRI images, which show through in excluded regions. The velocity magnitudes are similar for the five mice. (**C**) The MR-AIV–inferred velocity magnitude in two planes (midsagittal, left; transverse plane, right).

We injected gadobutrol into the cisterna magna of five wild-type mice and acquired DCE-MRI data. This yielded a time-dependent change in tracer intensity, or signal enhancement ratio (SER), which we normalized by its maximum value and multiplied by 100, using the result as a proxy for concentration *c* across the brain in three dimensions (Fig. 1A and fig. S7A).

For each experiment, an MR-AIV model predicts the velocity u in the deep brain (Fig. 1 and movie S1). We observe a consistent structure across all five mice, as expected if anatomical brain structures shape the flow. Velocities are high in the olfactory bulb, near a well-known large subarachnoid space (*37*). Chen *et al.* (*23*) also found high velocities in the olfactory bulbs of rats, using OMT. We also observe high velocities near the Circle of Willis and, in some mice, around the ventricles, suggesting flow between tissue and ventricles.

### MR-AIV reconstructs concentration fields from sparse observations

**Fig. 2.**
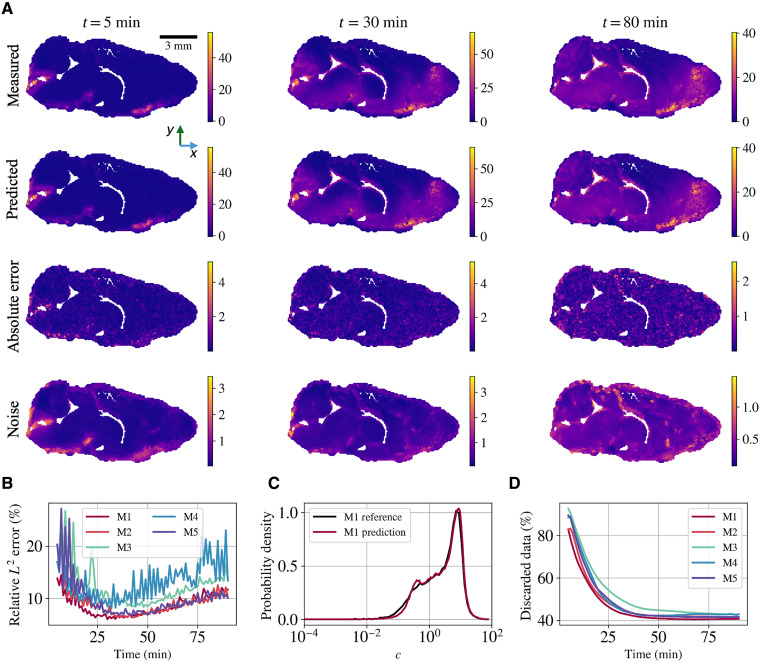
Concentration reconstruction using in vivo data. (**A**) Measured concentration, reconstructed concentration, absolute error, and inferred noise for mouse 1 (M1) on the midsagittal plane at *t* = 5, 30, and 80 min. The unstructured absolute error aligns spatially with the inferred noise, suggesting that discrepancies are dominated by measurement uncertainty. (**B**) Relative *L*^2^ error over time for all five mice (M1 to M5). The error drops sharply after the initial 10 to 20 min, averaging around 10%. (**C**) Probability density functions (PDFs; log scale) of measured and reconstructed concentration for M1. The model accurately captures the bimodal distribution, which spans six orders of magnitude. (**D**) Percentage of data discarded over time due to sensitivity thresholds. Data are discarded only from the differential equation constraint during velocity inference, not from concentration training. Discard rates drop from 90 to ∼40% as the tracer spreads; higher discard rates correlate with larger reconstruction errors.

No previous measurements of flows deep in the brains of mice are available for comparison, so other assessments are required. One way to test MR-AIV is to compare the concentrations it reconstructs to the measured concentrations. We train our models on 50% of the concentration data, used both to learn the concentration field and to extract aleatoric uncertainty via the denoising module. Model performance is evaluated with the unseen data. In mice 1 to 5, the relative *L*^2^ error (see Eq. 8) was 8.63, 9.07, 12.21, 13.14, and 9.83%, respectively. Figure 2A shows the reconstructed concentration, pointwise error, and learned noise for mouse 1 at three representative times, and the temporal evolution of the measured and reconstructed concentration is shown in movies S2 and S3. Predictions closely match measurements in magnitude and spatial variation. Notably, the absolute error appears unstructured, and the inferred uncertainty, trained to represent aleatoric noise, is spatially aligned with the absolute error. Most discrepancies seem to arise from measurement noise rather than modeling errors.

Figure 2B shows the temporal evolution of the relative *L*^2^ error for all five mice, which drops sharply after about 20 min, the time required for the tracer to reach a substantial fraction of the brain, and is steadier thereafter. Probability density functions (PDFs) of the reconstructed and experimental concentrations for mouse 1 (Fig. 2C) agree closely across six orders of magnitude. The concentration is bimodal. Most data from the first 20 min of an experiment are discarded because the concentration and its gradients are too small (Fig. 2D). Once the tracer has spread, more measurements are retained, but about 40% of the brain is never reached by enough tracer to be useful for the MR-AIV model. Some brain regions are apparently perfused much less than others. Experiments where more data were discarded generally show larger relative *L*^2^ error.

### MR-AIV reveals bimodal velocity distributions across distinct brain regions

**Fig. 3.**
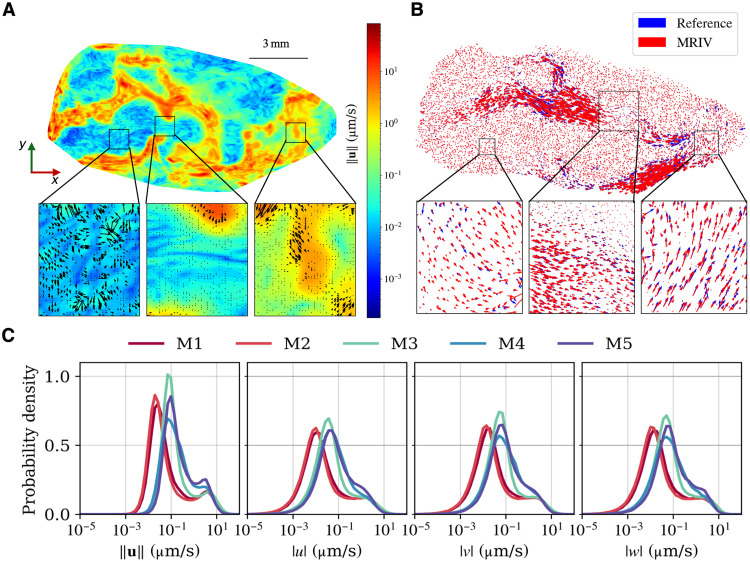
MR-AIV infers high-resolution 3D velocity fields. (**A**) Inferred in vivo velocity field from mouse 1. The high-resolution contour plot shows the velocity magnitude ∥u∥, while the overlaid vectors (quiver plot) illustrate the flow direction. The zoomed-in regions highlight that the inferred vector field is highly structured and spatially organized. (**B**) Vector alignment validation on a realistic synthetic dataset. MR-AIV inferred vectors (red) are coplotted with the ground-truth reference vectors (blue) obtained using a finite element method. The zoomed-in regions demonstrate strong directional agreement and vector overlap, confirming the model’s orientation fidelity. This qualitative agreement is quantified by a mean angular error of $\theta =23.9^{{}^{\circ}}$. (**C**) PDFs for the inferred in vivo velocity magnitude ∥u∥ and the individual velocity components (∣u∣, ∣v∣, and ∣w∣) for five mice (M1 to M5). The consistent distributions across all subjects and components indicate a robustness in the inferred flow.

MR-AIV provides a continuous, differentiable function that approximates the underlying physical fields while satisfying the governing partial differential equations (PDEs). This framework allows for a high-resolution, continuous prediction of the velocity field. As an indicator of the in vivo directional fidelity, Fig. 3A presents the inferred velocity vectors for mouse 1, which are visibly structured and spatially organized. To validate the model’s capability to reconstruct flow orientation, we compared its performance against a ground-truth, realistic synthetic dataset. Figure 3B shows that the inferred MR-AIV vectors (red) are highly aligned with the numerical reference vectors (blue), even in low-velocity regions, confirming the model’s ability to reconstruct the vector field. This directional fidelity is quantified in table S1, which reports a mean angular error of $\bar{\theta }=23.9{}^{\circ}$ for the realistic case. The full details of the numerical simulations, along with further validation across three different synthetic cases, are detailed in the subsequent sections and in the Supplementary Materials.

**Fig. 4.**
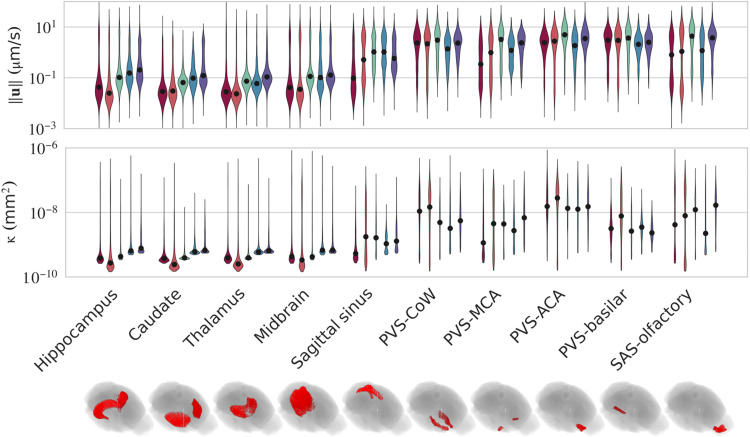
MR-AIV reveals anatomically distinct flow regimes and permeability distributions. The inferred velocity magnitude and permeability distributions in select anatomical regions. The median value is indicated with a black dot. Flows in the deep brain (e.g., hippocampus, caudate, thalamus, and midbrain) are slower. Faster flow and higher inferred permeability values are observed in the PVSs of the sagittal sinus, Circle of Willis (COW), middle cerebral artery (MCA), anterior cerebral artery (ACA), basilar artery, and subarachnoid space of the olfactory bulb.

By inferring 3D velocity throughout the brain, MR-AIV makes detailed velocity statistics available. PDFs of velocity magnitude in all five mice are bimodal, with a low peak near 3 μm/s and a high peak on the order of 0.1 μm/s (Fig. 3C). Bimodal distributions are consistent with the inferred velocity fields (Fig. 1), which show slow flows in most of the brain but fast flows in a few regions. Individual velocity components showed similar distributions, although the peak near 3 μm/s was less distinct. By segmenting the inferred velocity maps according to the Allen Brain Atlas, a process whose accuracy stands to benefit from the continued development of next-generation atlases (*38*), we calculated velocity and permeability statistics for specific anatomical regions (Fig. 4 and tables S2 to S5). Anatomical velocity variations were similar for all five mice, with slow flow in the hippocampus, caudate, thalamus, and sagittal sinus, but fast flow in the subarachnoid space near the olfactory bulb and near PVSs adjacent to the Circle of Willis, middle cerebral artery, anterior cerebral artery, and basilar artery. Permeability tended to be high in the regions where flow was fast and low in the regions where flow was slow (fig. S7E). According to Darcy’s law, velocity is the product of permeability and pressure gradient, so the observed similarity between velocity and permeability suggests that pressure gradients have similar magnitudes throughout the brain.

### MR-AIV delineates advection- and diffusion-dominated transport

**Fig. 5.**
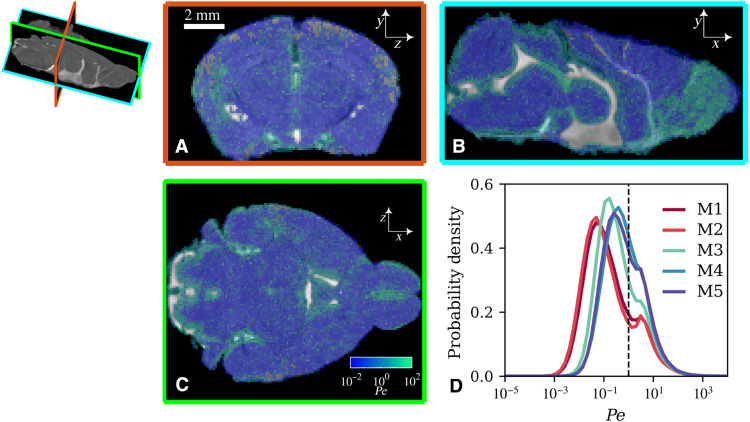
The local Péclet number varies spatially and is similar across mice. (**A** to **C**) Local Péclet number on mid-coronal (*z-y*), sagittal (*x-y*), and transverse (*x-z*) planes in mouse 1. Solute is transported primarily by advection in green regions and diffusion in blue regions. A grayscale structural MRI image is visible in excluded regions. (**D**) The probability densities of local Péclet number throughout the brains of five mice (M1 to M5).

An important question for brain clearance concerns the relative importance of advection and diffusion in transporting solutes. Because MR-AIV infers both concentration and velocity fields, the advective and diffusive transport terms can be calculated at any point in space and time (see Eq. 6). Their time-averaged ratio is a local Péclet number *Pe*; Pe≫1 where advection dominates, and Pe≪1 where diffusion dominates. Across the brain, *Pe* varies by several orders of magnitude, with some regions dominated by advection and others dominated by diffusion (Fig. 5 and movie S4). Diffusion dominates in most of the brain, but advection dominates in many regions where the velocity is large, e.g., near the olfactory bulb, cisterna magna, and Circle of Willis (Fig. 5, A to C). Because *Pe* is proportional to the velocity, it is not unexpected that high-velocity regions often overlap high-*Pe* regions. That said, maps of ‖u‖ and *Pe* are not identical, and PDFs of *Pe* are less bimodal than PDFs of ‖u‖, so diffusion must also affect *Pe*. The PDF of *Pe* is similar for all five mice, suggesting that the distribution results from shared anatomical features.

### MR-AIV maps permeability and pressure

**Fig. 6.**
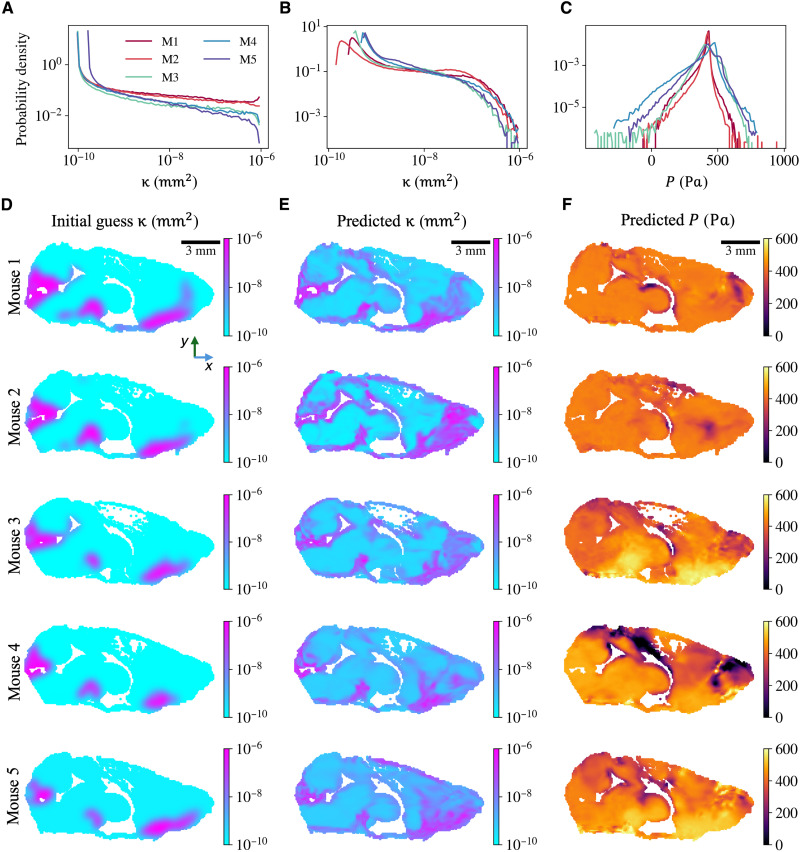
Estimated permeability and pressure. (**A**) PDFs of the initial permeability guess κ for each mouse (M1 to M5). All distributions span more than four orders of magnitude, from 10^−10^ to 10^−6^ mm^2^, with little variability across mice. PDFs of the estimated (**B**) permeability κ and (**C**) pressure *P* after training. (**D**) Initial permeability guess for each mouse (M1 to M3). Whereas the overall dynamic range is consistent, spatial distributions differ across mice. Predicted (**E**) permeability κ and (**F**) pressure *P* are spatially coherent and consistent across five mice.

Maps of inferred permeability exhibit consistent spatial patterns across all five mice (Fig. 6) and show, in more detail, the anatomical variation discussed above: high permeability around the olfactory bulb and PVSs. Inferred permeability maps are similar across mice but differ from initial guesses, suggesting that spatial variations result from anatomical features. Inferred pressures are similarly consistent across mice. The initial permeability guesses (Fig. 6, A and D) are derived from the measured concentration field c and based on the reasoning that high permeability/low resistance regions would approximately correspond to regions of early tracer arrival, as described in Materials and Methods.

To the best of our knowledge, no current experimental method provides a direct, high-resolution validation of spatial pressure maps in vivo. Furthermore, the inverse problem is inherently ill-posed, because the velocity u is a function of two spatially varying unknown fields, K(x) and P(x). Our framework attempts to control this ambiguity by using data-driven initial permeability and velocity guesses (derived from early tracer arrival and front tracking, respectively) as a strong prior. The plausibility of this data-driven approach is supported by the final result, which indicates that our inferred pressure drop (ΔP∼800 Pa, Fig. 6C) is consistent with the experimentally measured range for absolute baseline intracranial pressure in anesthetized mice, which is reported to be between 4.11 mmHg (≈548 Pa) and 12.0 mmHg (≈1600 Pa) (*39*–*45*). Nevertheless, given this reliance on a prior and the inherent ill-posedness, the inferred *P* and *K* fields should be considered a physically plausible solution set, rather than a unique ground truth. This ambiguity is further highlighted in our analysis using synthetic data (table S1), which shows that models can achieve low velocity error while having higher errors in the permeability and pressure fields. However, this framework also opens an avenue for future studies to incorporate more accurate, anatomy-based permeability priors to further refine these estimates.

### MR-AIV eliminates noise, trains on data, and then trains on physics

The MR-AIV framework begins with preprocessing, where initial estimates of permeability *K* and u are obtained, then an initialization step that concurrently denoises the concentration data and aims to match the initial estimates, and a training stage, in which the inferred fields are refined to satisfy the governing physical laws (see Materials and Methods). During the preprocessing stage, we estimate u using front tracking (*4*, *11*, *12*).

The permeability of brain tissue is unknown, with estimates spanning multiple orders of magnitude (*46*), so in MR-AIV, the model learns the permeability, given an initial estimate. That estimate is made by assuming that regions quickly reached by tracer have high permeability and that other regions have permeability 10^4^-fold lower and by subsequently applying some smoothing.

**Fig. 7.**
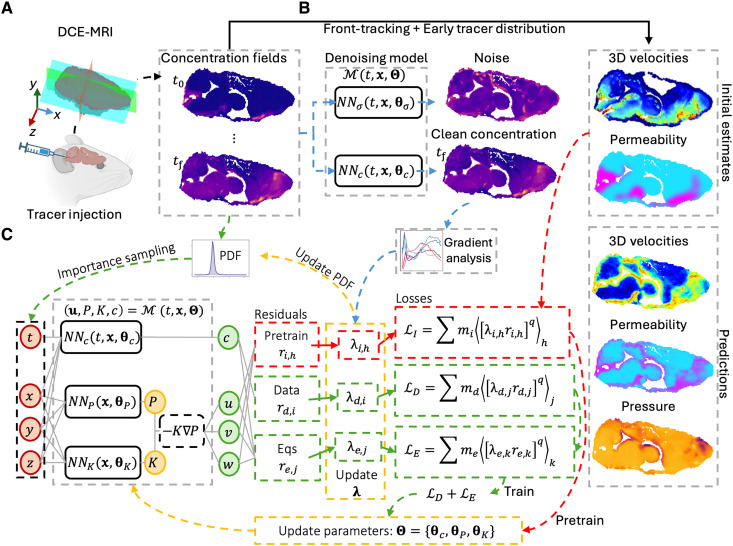
MR-AIV framework. MR-AIV infers 3D steady velocity u, permeability *K*, and pressure *P* fields from time-dependent concentration data. (**A**) Brain-wide concentration fields are obtained by injecting gadobutrol and tracking the tracer using DCE-MRI. (**B**) Initial estimates for u and *K* are derived from front tracking and early tracer distributions. A denoising module learns to separate experimental noise from the underlying signal (blue path). (**C**) The architecture enforces Darcy’s law and steady-state conditions using independent networks for concentration, pressure, and permeability (${\mathrm{NN}}_{c}$, ${\mathrm{NN}}_{p}$, and ${\mathrm{NN}}_{k}$). The model is initialized by matching the initial estimates (red path) and then refined by minimizing the residuals of the governing equations and concentration data (green path). Convergence is enhanced by TD-RBA, which uses weights (λ) based on residuals and clean concentration gradients to dynamically resample high-error regions, thereby improving convergence across the entire domain.

During the first step of the initialization (see Fig. 7B), we account for noise in experimental data by incorporating two neural networks, ${\mathrm{NN}}_{c}$ and ${\mathrm{NN}}_{\sigma }$, which learn the mean concentration field and its associated time-dependent SD (i.e., noise), respectively, by minimizing the negative log-likelihood of the observed concentration data (*47*).

As shown in Fig. 7C, the MR-AIV architecture ℳ(x,Θ) with learnable parameters $\Theta =\left\{\theta_{c},\theta_{P},\theta_{K}\right\}$ consists of three neural networks: ${\mathrm{NN}}_{c}\left(t,x,\theta_{c}\right)$, which predicts the time-dependent concentration field *c* at any location x=(x,y,z); and ${\mathrm{NN}}_{P}\left(x,\theta_{P}\right)$ and ${\mathrm{NN}}_{K}\left(x,\theta_{K}\right)$, which predict the steady-state pressure P(x) and permeability K(x), respectively. (We define the *x*, *y*, and *z* directions to be normal to the transverse, coronal, and midsagittal planes, respectively.) The velocity u=(u,v,w) can then be calculated via Darcy’s law: u=−K∇P.

During the second step of the initialization stage, the pressure and permeability networks are optimized using a data-driven approach that minimizes a loss function $L_{I}$, which penalizes the residuals $r_{i}$ (i.e., pointwise errors) between the initial estimates and the predictions. Last, during the training stage, predictions are refined by optimizing a combined loss function $L_{D}+L_{E}$ that minimizes the residuals of measured concentration ($r_{d}$) and the governing equations ($r_{e}$) (see Fig. 7C).

To promote uniform convergence during training, we introduce TD-RBA. This weighting approach improves previous residual-based attention (RBA) strategies (*36*, *48*–*52*) by computing attention weights $\lambda_{i}$ based on the residual magnitudes and a physics-based time dependent scalar computed from the spatial gradients of the clean concentration field. TD-RBA are crucial for minimizing the multiscale residuals of governing equations and are used both as local multipliers to balance pointwise errors in the loss function and to define a residual-based PDF for resampling high-error regions. This resampling procedure improves predictions, allowing the model to focus training on regions with large residuals while maintaining stability across the full spatiotemporal domain. A comprehensive description of the MR-AIV architecture, sequential training strategy, and the proposed (TD-RBA) is presented in Materials and Methods and the Supplementary Materials.

### MR-AIV is validated with synthetic data, and uncertainty with real data is quantified

To evaluate MR-AIV, we conducted a validation study on three synthetic datasets generated using finite element simulations that provided ground-truth velocity, pressure, and permeability fields (see figs. S5 and S6). Our validation confirmed that the framework successfully reconstructs concentration fields with high accuracy (relative *L*^2^ error <2%, fig. S4). For the more challenging task of velocity inference, performance depended on the complexity of the underlying permeability map. For the most complex realistic case, the average relative *L*^2^ error for the total velocity magnitude was 36.0% (see table S1). However, as shown in fig. S5, this error is not uniform; it is overwhelmingly concentrated in extremely low-velocity regions. This pattern is an expected consequence of the data: In slow-flow regions, minimal tracer is transported, resulting in a weak concentration signal that provides little information to constrain the velocity inference. In a simpler smooth case, the model performed optimally, with 88.5% of the domain having a relative error below 25% (see fig. S5A).

In addition to magnitude error, the model’s ability to capture key distributional and directional features was also assessed. Our componentwise analysis (table S1) details the relative *L*^2^ error for u, v, and w individually. The errors for the v and w components are observed to be slightly higher, which is an expected artifact of the relative error metric as the true magnitudes of these components are significantly smaller than the primary flow components. More importantly, the Wasserstein distance ($W_{d}$) for the distributions of all three components remains low, indicating the model correctly captures their statistical profiles. This is further supported by the model’s ability to capture the bimodal velocity distribution (fig. S5B), a key physical feature not retained by standard PIML models. Moreover, Fig. 3B confirms that the predicted direction is highly consistent with the ground truth for the more complex realistic case. Crucially, MR-AIV also successfully captured the correct spatial distributions of permeability and pressure (see fig. S6), providing the first-ever estimates of these fields.

**Fig. 8.**
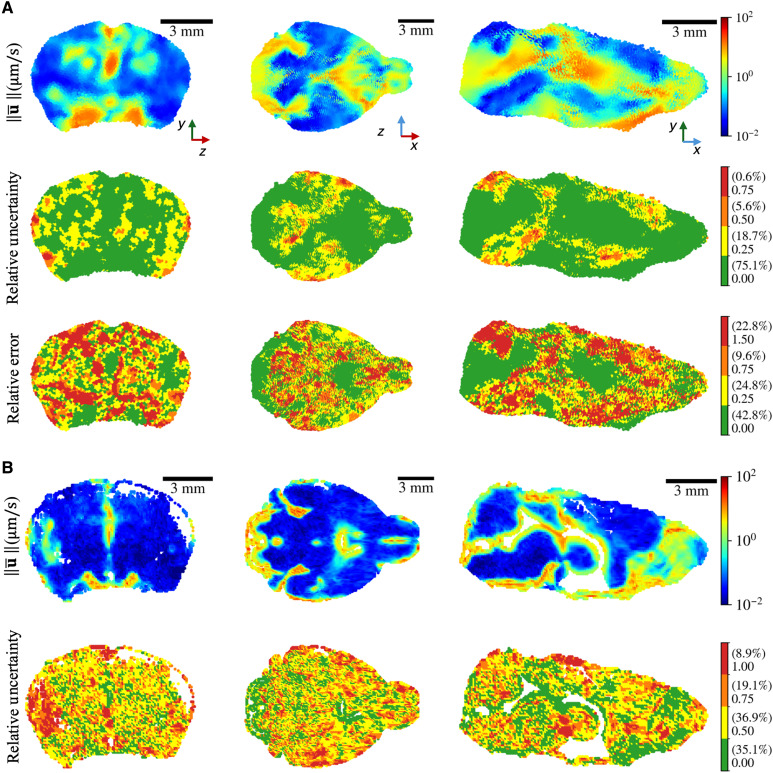
Uncertainty quantification of inferred velocity fields. (**A**) Uncertainty quantification for realistic synthetic data. The top row shows the velocity magnitude $∥\bar{u}∥$ averaged across an ensemble of four models. The middle row shows the relative uncertainty $\sigma_{∥u∥}/∥\bar{u}∥$, and the bottom row shows the pointwise relative error compared to ground truth. High uncertainty correlates with high error, and both are concentrated in low-velocity regions. The average relative *L*^2^ error for the ensemble is 34%. Parenthetical values indicate the percentage of total brain volume in each color. (**B**) Uncertainty quantification for mouse 1. The top row shows the velocity magnitude averaged across the ensemble, and the bottom row shows the relative uncertainty. Consistent with the synthetic data, uncertainty is highest in low-velocity regions yet remains below 100% across 90% of the domain.

We then performed a robustness analysis on the initial permeability guess, which, in turn, assesses the epistemic uncertainty of our framework. In our model, the initial permeability guess effectively defines the parameter initialization for the subsequent velocity inference stage. We therefore quantified this uncertainty using an ensemble-of-models approach, training multiple models with different initial permeability guesses. For the synthetic data, this analysis revealed a strong correlation between the predicted relative uncertainty and the true pointwise error (Fig. 8A), validating our uncertainty estimate as a reliable proxy for accuracy. When applied to the in vivo data, the uncertainty was highest in low-velocity regions, consistent with the pattern that we observed in the synthetic data (Fig. 8B). Despite the inherent difficulty of the problem, the uncertainty remained below 100% across 90% of the brain, providing informative, first-of-their-kind measurements of deep brain flow dynamics.

## DISCUSSION

In summary, we introduce MR-AIV, a specialized PIML framework designed to infer steady, brain-wide velocity, pressure, and permeability from time-dependent DCE-MRI concentration data. MR-AIV can infer flows in the brain from sparse, noisy data without requiring velocity measurements, offering a generalizable and data-efficient tool for modeling transport in biological systems. We validated MR-AIV on synthetic data designed to mimic DCE-MRI measurements of tracer injected in the brains of mice and observed good agreement (see fig. S5). An uncertainty quantification analysis demonstrated the robustness of the inferred velocities. We have made the MR-AIV source code freely available.

We used MR-AIV to quantify fluid flow and Péclet number throughout the entire brains of five wild-type mice. Typical velocity magnitudes, Péclet numbers, and anatomical variations of both were broadly similar across mice, suggesting that the observed features are robust. PVSs and subarachnoid spaces harbored fast flow, while flow elsewhere was much slower, supporting the idea that PVSs and subarachnoid spaces serve a distinct role as high-speed pathways (*53*). That distinct role is further supported by maps of the Péclet number: In small regions around PVSs, ventricles, and the olfactory bulb, Pe≫1, implying advective solute transport, but elsewhere, Pe≪1, implying diffusive solute transport. The finding of rapid, advection-dominated transport in the olfactory bulb is particularly significant as this area has been identified as a primary entry route for environmental neurotoxins, such as inhaled iron nanoparticles that contribute to sex-specific, neurodegenerative pathologies (*54*). Local velocity is apparently determined more by variations of permeability, which spans four orders of magnitude and correlates strongly with velocity (see fig. S7E), than by variations of pressure, which spans just one. Thus, the brain seems to regulate flow primarily through tissue properties, not pressure sources. Across mice, permeability was large near ventricles, the olfactory bulb, the cisterna magna, and the Circle of Willis (see Fig. 4). Pressure was low in the olfactory bulb and high near the Circle of Willis. Inferred pressures reached 600 Pa, lower than the typical cardiac pulse pressure and of the same order as numerical estimates (*55*). Future measurements of pressure gradients could test these predictions. We repeatedly observed fast flows near the ventricle walls; future work should explore the possibility of substantial flow there.

For each mouse, the largest peak in the PDF of velocity magnitude occurred near 0.1 μm/s, corresponding to velocities common in most of the parenchyma. For comparison, Ray *et al.* (*27*) estimated interstitial flow velocities of 0.1 μm/s by incorporating the possibility of bulk flow into a transport model of real-time iontophoresis and comparing with experimental measurements in mice. Vinje *et al.* (*25*) used forward and inverse subject-specific modeling to show that tracer transport in humans could be explained by a combination of diffusion and interstitial flow velocities of 0.017 to 0.15 μm/s. Chen *et al.* (*23*) estimated velocities of 0.1 μm/s in the brain parenchyma of rats using DCE-MRI and regularized OMT (rOMT), an inverse method based on minimizing tracer displacement (*20*–*24*). Thus, MR-AIV infers parenchymal velocities consistent with several prior estimates and supports the idea of slow but important bulk flow of ISF (*56*).

For each mouse, another peak in the PDF of the velocity magnitude occurred near 3 μm/s, corresponding to velocities common in open regions and similar to the 2 to 5 μm/s previously estimated for penetrating and capillary PVSs and parenchyma (*14*) and the ∼20 μm/s measured in pial PVSs (*6*, *8*, *9*, *57*). The fact that PVSs are smaller than a voxel probably contributes to our velocities, which are necessarily averaged over a voxel, being comparatively slow. Chen *et al.* (*23*) estimated velocities of 0.2 μm/s in large surface PVSs, 15-fold slower than the ∼3 μm/s inferred by MR-AIV. The difference may be explained by the fact that Darcy’s law is used in MR-AIV but not in rOMT. In particular, by incorporating Darcy’s law, our framework captures the multiscale nature of brain fluid flow, distinguishing high-flow conduits from the parenchyma, a distinction that is less pronounced in methods that do not enforce this physical constraint.

When we made models without Darcy’s law, predicted velocities spanned a smaller range and were unimodal, failing to distinguish high-speed pathways from other regions (fig. S8C). Although Chen *et al.* (*23*) did not report PDFs of velocities, the fact that they found velocities in pial PVSs exceeding those in porous regions by only a factor of two suggests a narrower distribution, consistent with what we observed when we did not use Darcy’s law. Another possible explanation is image resolution: Compared to the 100-μm voxels used here, the 234-μm voxels of Chen *et al.* (*23*) would introduce stronger partial-volume effects. Voxel volumes being 12.8-fold larger might explain the estimated velocities being 15-fold smaller. On the other hand, the largest PVSs are bigger in rats than in mice, partly alleviating partial volume effects.

MR-AIV inferences from synthetic data showed higher errors and uncertainties in regions with slow flow (see figs. S5 and S8). There, diffusion tends to flatten concentration gradients, while advection transports tracer only weakly, so the effective signal-to-noise ratio for inferring velocity from advection is low.

Additionally, little signal is available in slow regions because the tracer tends to appear there only near the end of an experiment or simulation. At each time during an experiment, at least 40% of the concentration data was discarded because its local magnitude or gradient was too small to be useful. Despite sometimes having relatively large uncertainty (∼100%) in the low velocity regions (see Fig. 8), inferences from MR-AIV are nonetheless informative, considering the scarcity of published measurements of flow velocities in the deep brain. The ability to estimate uncertainty with MR-AIV allows systematic improvement in future work.

The synthetic data used for validation were made as realistic as possible (see fig. S3), with the domain shape taken from mouse 1, and the inlet and outlet pressure boundary conditions chosen to produce velocity magnitudes similar to those inferred from the real data (Fig. 8). The resulting velocity map matched anatomical expectations, with high velocities in the olfactory bulb, near the cisterna magna, and near the Circle of Willis and with low velocities in the brain’s interior.

Additionally, we calculated the epistemic uncertainty arising from the initial guess for the permeability, using the same permeability maps that we used for the in vivo data, and we saw similar trends in the uncertainty map. Synthetic data captured key features of in vivo data, like bimodal velocity distributions that span more than four orders of magnitude. Our synthetic data are freely available and can be used to benchmark alternatives to MR-AIV. One limitation of the synthetic data is that it is constructed under the assumption of Darcy flow throughout the brain, despite the presence of regions where Stokes flow is known to occur. Those regions are accounted for with the spatially varying permeability, where regions with open flow have a high permeability because they are typically subvoxel in size, so their location cannot be determined from MRI data.

MR-AIV infers permeability, pressure, and velocity by assuming that the observed concentration is governed by the advection-diffusion equation, the continuity equation, and Darcy’s law. Combining these three equations yields two expressions (Eq. 4) involving products of permeability and pressure (or their derivatives). However, except in products, permeability and pressure do not appear, so multiple combinations of the permeability and pressure can yield identical concentrations and velocities: scaling permeability by a constant factor and pressure by the inverse of that factor has no effect on velocity. Without additional constraints, the inferred pressure and permeability fields are not unique (although velocity is).

That said, the spatial variations of permeability and pressure, aside from the scaling factor, are fully captured by Eq. 4. Consistent with that fact, MR-AIV models trained on synthetic data infer the spatial variations accurately.

In vivo, true permeability is not known, but permeabilities inferred from experiments were more similar across mice than to the initial permeability guess (Fig. 6, D and E), and four different initial guesses resulted in similar inferred permeability fields (fig. S9), further supporting the idea that inferred permeabilities are valid. Future measurements of the absolute permeability or pressure in even one location would eliminate ambiguity from Eq. 4 and allow MR-AIV to make unique inferences.

We constructed an initial guess of the permeability purely from the tracer concentration soon after injection (see Materials and Methods). This data-driven approach is repeatable and avoids bias associated with extensive user input. However, in future work, better guesses might come by combining early measurements with anatomical knowledge. In our guess, the maximum permeability was set by calculating the equivalent resistance of an open, 6-μm cylindrical vessel. Many open structures are larger and presumably allow faster flows, but nearly all are also smaller than a voxel, so voxel-averaged velocities and effective permeabilities would be reduced, offsetting the large size. Because the amount of reduction is unknown, the maximum permeability is uncertain. For low-permeability regions, published estimates vary widely, from 1 × 10^11^ to 4.50 × 10^−9^ mm^2^ (*28*, *58*). We chose 1 × 10^−10^ mm^2^, although, again, the value is uncertain. Accordingly, we estimated the epistemic uncertainty arising from the initial permeability field guess because we consider that it is the largest source of uncertainty in our modeling. More accurate measurements of brain permeability would substantially reduce the uncertainty stemming from the initial permeability guess. That said, uniformly scaling the entire initial permeability guess by a constant factor, even by an order of magnitude, changes our results little because the initial guess is nondimensionalized and scaled during calculations. As shown in eq. S1, a scaling factor would effectively redefine the characteristic permeability and reciprocally modify the characteristic pressure without changing the velocity.

We used the scaled SER as a proxy for tracer concentration, implying a linear relationship between the two quantities. Ratner *et al.* (*20*), using a similar imaging protocol, suggested that the relationship is nearly linear for SER less than 200%, which is true for >99% of our individual measurements.

MR-AIV is limited to approximating velocities, permeabilities, and pressures as steady. CSF flows fluctuate in response to cardiac, respiratory, and slow vasomotor activity and can vary over time. However, our experiments were performed with unperturbed, anaesthetized mice whose brain activity presumably varied relatively little. Slow vasomotion is suppressed during anesthesia (*59*–*61*), and cardiac and respiratory fluctuations occur at a much higher frequency than the DCE-MRI acquisitions and are relatively stable during anesthesia, allowing us to infer the time-averaged flow field.

Considering the prevalence of DCE-MRI data, the fact that it is one of the few approaches for visualizing in vivo fluid flows in humans, and the insight into the glymphatic system that this imaging modality has already provided, brain-wide velocity fields inferred by MR-AIV may unlock key questions about glymphatic flows in the future. Integrating these novel fluid dynamics maps with state-of-the-art anatomical resources, such as high-resolution, distortion-corrected brain atlases (*38*, *62*), and correlating them with other advanced modalities that probe tissue microstructure (*63*), represents a promising direction for creating a more complete biophysical model of the brain.

## MATERIALS AND METHODS

### Imaging data collection and preprocessing

Five 11- to 13-week-old male C57BL/6 wild-type mice were used for this experiment. The MRI data were collected in a 9.4-T preclinical scanner (BioSpec 94/30 USR, ParaVision 6.0.1 software, Bruker BioSpin, Ettlingen, Germany) equipped with a 1H cryogenically cooled quadrature-resonator transmit/receive coil (CryoProbe, Bruker) and a gradient coil of 240 mT/m (BGA-12S, Bruker) at the Preclinical MRI Core Facility, University of Copenhagen. During the imaging, all the mice in the MRI scanner were placed in the prone position and anaesthetized with the intraperitoneal injection of the mixture of ketamine and dexmedetomidine (75/1 mg/kg). The body temperature was maintained at 37°±1°C with a magnetic resonance–compatible and thermostatically controlled waterbed and monitored by a remote monitoring system (SA Instruments, NY, USA), together with the respiratory rate. A stereotactic holder with ear bars was used to minimize the head movement during imaging. To investigate fluid flow dynamics, a contrast agent was injected intracisternally as previously described (*64*, *65*). Briefly, a 30-gauge copper needle (outer diameter, 0.32 mm; Nippon Tokushukan, Mfg, Tokyo, Japan) attached to a PE10 tubing was inserted into the cisterna magna. A T2-weighted structural image was conducted using 3D constructive interference steady state (3D-CISS). Each 3D-CISS image was calculated as a maximum intensity projection of four realigned 3D True Fast Imaging with Steady-state Precession (3D-TrueFISP) volumes with four orthogonal phase encoding directions [repetition time (TR)/echo time (TE), 3.9/1.95 ms; number of excitation (NEX), 1; flip angle (FA), 50°; field of view (FOV), 19.2 mm by 12.8 mm by 12.8 mm; matrix, 192 × 128 × 128]. For DCE-MRI, T1-weighted imaging was acquired using a 3D Fast Imaging with Steady-state Precession (3D-FISP) sequence (TR/TE, 4/2 ms; NEX, 1; FA, 15°; FOV, 19.2 mm by 12.8 mm by 12.8 mm; matrix, 192 × 128 × 128). First, three baseline DCE-MRI scans (3 min) were acquired. T1-enhancing contrast agent gadobutrol (15 mM; Gadovist, Bayer Pharma AG, Leverkusen, Germany) was infused into the cisterna magna. Over a 10-min period, 10 μl of gadobutrol was infused at a rate of 1 μl/min using a 100-μl Hamilton syringe mounted on a motorized pump. The image spatial resolution was 100 μm isotropic (0.001 mm^3^) with one scan every 60 s. After the three baseline scans, the follow-up scans continued over 90 min. Further image processing pipelines were applied, including motion correction, bias field correction, and spatial coregistration between 3D-CISS and DCE-MRI images. To normalize the image in each time series, their voxel intensities were subjected to Gaussian normalization using the first 3D-FISP volume. The resulting images were smoothed with a 3 × 3 × 3–voxel kernel of [0.2, 1, 0.2] weights along each axis, to reduce the influence of possible artefacts after the automatic registration and subtracting the baseline volume. Voxel-based percentage enhancement of contrast from baseline (SER) was calculated as equation of $\text{SER}=\left(S_{t}-S_{0}\right)/S_{0}\times 100$ (fig. S7A).

To avoid using a source term in our governing equations, we excluded the concentration data in the cisterna magna. We also excluded the ventricles, where CSF is produced, regions outside of the brain, and regions that the tracer did not reach during the 93-min scan duration (defined as regions where the change in SER through all scans was less than three times the change in SER during the baseline portion of the scan). The excluded regions are shown on a midsagittal slice in fig. S7B. Accordingly, there are no concentration, velocity, or permeability predictions in the excluded regions.

### Initial velocity estimate from front tracking

We used front tracking to provide an initial estimate of the velocity field, which is used to initialize the neural networks. In front tracking, fronts, or regions of constant concentration, are tracked over time and space. Front tracking neglects diffusion and thus has low accuracy in regions where diffusion plays a substantial role in transport. The front tracking algorithm that we use is inspired by one described by Nevins and Kelley (*66*, *67*) but has been adapted for three dimensions and does not fit curves to the fronts, as further described in the Supplementary Materials. We combine sparse velocity information from many different fronts corresponding to various concentrations and assume the flow is steady, thereby obtaining estimates of the velocity at nearly every location tracer reaches in the brain. Figure S7D shows example velocity estimates from front tracking.

### Material property estimates

The material properties of CSF and ISF are similar to those of water, so we used the density and viscosity of water at 37°C (density ρ = 993 kg/m^3^, viscosity μ = 6.95 × 10^−4^ Pa · s). We used *D* = 2.4 × 10^−4^ mm^2^/s for the diffusion coefficient of gadobutrol. The free diffusion coefficient for gadolinium diethylenetriaminepentaacetic acid (Gd-DTPA), which has a similar molecular weight as gadobutrol (550 Da compared to 604 Da) is $D_{\text{free}}=3.8\times 10^{-4}$ mm^2^/s (*13*), but because most of the brain tissue is porous, the effective diffusion coefficient in the porous regions is $D_{\text{eff}}=1.48\times 10^{-4}$ mm^2^/s (where $D_{\text{eff}}=D_{\text{free}}/\lambda^{2}$, assuming the tortuosity is λ = 1.6, which has been repeatedly measured for ECS). This also agrees with estimates of the diffusion coefficient from Ringstad *et al.* (*68*). Because the diffusion coefficient number only varies by a factor of 2.5 between open and porous regions, we used the constant value $D=2.4\times 10^{-4}$ mm^2^/s everywhere in the domain.

Brain tissue is typically modeled as a porous medium, but the permeability is highly uncertain (*46*). Furthermore, while the brain tissue may resemble a porous medium, it is filled with open channels where CSF flows rapidly, like the ventricles, subarachnoid spaces, and pial PVSs (*7*, *69*). Identifying open spaces within the brain, particularly those that are smaller than an MRI voxel (e.g., PVSs), is not trivial, and most voxels containing “open” spaces also contain porous tissue. Therefore, we modeled the entire brain as a porous medium with spatially varying permeability to be learned by the neural network. We provided an initial estimate of the permeability map by assuming that regions that tracer reached (defined as having a SER greater than 150%) quickly (within 16 min) had high permeability (1 × 10^−6^ mm^2^, the effective permeability of an open cylindrical channel with a diameter of 6 μm), and all other regions had low permeability (1 × 10^−10^ mm^2^). Although high-permeability voxels are likely to contain open spaces larger than 6 μm, because they also contain porous medium, we did not set the effective permeability higher. The most accurate permeability would be a weighted average across the voxel’s volume, but no methods yet exist to provide the necessary information. The value for the low permeability was chosen on the basis of estimates for the permeability of brain tissue in the literature (*28*, *58*). We smoothed the binary permeability map using a 3D Gaussian filter with an SD of one or three voxels for the “sharp” and “smooth” cases, respectively. Because the high-permeability regions are small, smoothing sometimes lowers their maximum permeability; we then scaled κ to return its maximum to 1 × 10^−6^ mm^2^. The velocities shown in Figs. 1 and 4 come from the smooth initial permeability maps shown in Fig. 6.

### Underlying physical laws

Tracer transport was assumed to follow the advection-diffusion equation with constant diffusivity *D*

$$c_{t}+\left(u\cdot \nabla \right)c=D\nabla^{2}c$$ (1)

where *c* denotes the tracer concentration. The tracer evolves over the spatiotemporal domain $\Omega =\Omega_{x}\times \Omega_{t}\subset {\mathbb{R}}^{3+1}$, with $\Omega_{t}=\left\{t\in \mathbb{R}|t_{0}\leq t\leq t_{f}\right\}$. Here, $t_{f}=90$ min corresponds to the final acquisition time, and $t_{0}=5$ min is selected to exclude the initial injection phase, because the specific source term in the advection-diffusion equation was unknown. We assumed that the flow was incompressible and imposed conservation of mass in the form

∇⋅u=0 (2)

Notice that trying to reconstruct the three velocity components only from concentration observations using Eqs. 1 and 2 and no boundary conditions is an ill-posed problem. This challenge became evident in models that attempted to reconstruct the velocity field u directly using Eqs. 1 and 2, which led to poor performance and struggled to capture the true velocity distribution (see table S6 and fig. S5C). Therefore, to further constrain the model, we assume that the flow behaves as if in a porous medium and use Darcy’s law as an extra constraint. The porous medium assumption has been extensively explored for the brain in traditional numerical methods.

u=−K∇P (3)

Here, u is the velocity field, *K* is the hydraulic permeability, and *P* is the pressure. Incidentally, K=κ/μ, where κ is the intrinsic permeability and μ is the fluid viscosity. The velocity was assumed to be steady within the spatial domain $\Omega_{x}=\left\{x\in {\mathbb{R}}^{3}|x\in G_{m}\right\}$, where $G_{m}$ denotes the set of spatial locations defining the brain geometry of each individual mouse *m*. Notice that, under this assumption, we can represent Eqs. 1 and 2 as follows

$$c_{t}-\left[\left(K\nabla P\right)\cdot \nabla \right]c=D\nabla^{2}c$$ (4)

$$\left(\nabla K\right)\cdot \left(\nabla P\right)+K\nabla^{2}P=0$$ (5)

Because *D* is constant and we have observations of *c* (e.g., experimental data or simulations), this representation helps us tackle the ill-posedness because now we are only inferring 2 variables (*K* and *P*). Furthermore, the permeability field *K* acts as a learned spatial map, enabling the model to capture the sharp transitions between high- and low-velocity regions that span several orders of magnitude. Empirically, we observed that this reformulation was essential to obtain more accurate predictions and mainly to capture the bimodal distribution of the velocity as described in Results. Once *K* and *P* have been inferred, u can be calculated using Eq. 3.

These equations are nondimensionalized before use, a critical step for numerical precision, as the physical quantities span many orders of magnitude (e.g., κ ∼ 10^−10^ mm^2^). If used directly, gradient contributions from terms with small coefficients could potentially be lost due to single-precision (32-bit floating-point) arithmetic error, preventing the optimizer from learning these physical processes. This is a standard and recommended practice for improving stability in PIMLs (*70*, *71*). The full, nondimensionalized governing equations used in our framework are detailed in the Supplementary Materials.

#### Local Péclet number

The local Péclet number *Pe* is a dimensionless parameter that describes the relative contributions of advection and diffusion to mass transport, defined as

$$\mathrm{Pe}=\bar{\left|\frac{u\cdot \nabla c}{D\nabla^{2}c}\right|}$$ (6)

where the operator $\bar{\cdot }$ denotes an average over time.

### Magnetic resonance artificial intelligence velocimetry

MR-AIV is a scientific machine learning framework inspired by AIV (*9*, *10*, *35*) and by AIV and thermometry (*36*), designed to infer continuous and differentiable steady-state velocity fields u=(u,v,w) from time-dependent, sparse concentration measurements *c*. For robustness and stability (*70*), MR-AIV approximates the solutions of the governing Eqs. 3, 2, and 1 in their nondimensional form (see the Supplementary Materials)

$$\left(c,u^{*}\right)=ℳ\left(t^{*},x^{*};\Theta \right)$$

where Θ represents the trainable parameters of the model, $\left(t^{*},x^{*}\right)=(t^{*},x^{*},y^{*},z^{*})$ denotes the nondimensional temporal and spatial inputs, and *c* and $u^{*}=\left(u^{*},v^{*},w^{*}\right)$ are the nondimensional concentration and velocity fields (see the Supplementary Materials).

As shown in Fig. 7C, the proposed model ℳ(⋅) is composed of three independent neural networks: ${\mathrm{NN}}_{c}\left(\cdot \right)$, ${\mathrm{NN}}_{K}\left(\cdot \right)$, and ${\mathrm{NN}}_{P}\left(\cdot \right)$, which respectively approximate the nondimensional concentration (*c*), pressure ($P^{*}$), and permeability ($K^{*}$) fields

$$\begin{array}{c} c\left(t^{*},x^{*}\right)={\mathrm{NN}}_{c}\left(t^{*},x^{*};\theta_{c}\right),\\ P^{*}\left(x^{*}\right)={\mathrm{NN}}_{P}\left(x^{*};\theta_{c}\right),\\ K^{*}\left(x^{*}\right)=C_{1}\text{exp}\left\{C_{2}\sigma \left[NN_{K}\left(x^{*};\theta_{K}\right)\right]\right\} \end{array}$$

where $\theta_{c}$, $\theta_{P}$, and $\theta_{K}$ denote the parameters of each neural network. The sigmoid function σ:ℝ→[0,1] is used in combination with the constants $C_{1}=K_{\text{max}}$ and $C_{2}=\text{log}\left(K_{\text{min}}^{*}\right)-\text{log}\left(K_{\text{max}}^{*}\right)$ to ensure that permeability remains bounded between $K_{\text{min}}^{*}$ and $K_{\text{max}}^{*}$. The nondimensional velocity field $u^{*}$ is then recovered from $K^{*}$ and $P^{*}$ via $u^{*}=-K^{*}\text{∇}P^{*}$, thereby strictly enforcing the nondimensional Darcy’s law (see the Supplementary Materials). This formulation also inherently satisfies the steady-state assumption as both $K^{*}$ and $P^{*}$ depend solely on the spatial coordinates $x^{*}$.

For the network architecture, all models use the hyperbolic tangent (tanh) as an activation function, so their inputs are normalized between (−1,1) for stability (*70*). The specific architectures (layers and neurons) are described in Table 1. Furthermore, all MR-AIV subnetworks [${\mathrm{NN}}_{c}\left(\cdot \right)$, ${\mathrm{NN}}_{\sigma }\left(\cdot \right)$, ${\mathrm{NN}}_{K}\left(\cdot \right)$, and ${\mathrm{NN}}_{P}\left(\cdot \right)$] in this study use weight normalization (*72*) to speed up convergence, adaptive residual connections to avoid vanishing gradients (*73*), and a fifth-degree polynomial feature expansion to improve the model’s representation capabilities (*50*). The remaining implementation details are described in the Supplementary Materials.

**Table 1.** MR-AIV architectures. MR-AIV subnetworks’ number of adaptive residual layers (*73*) and neurons per layer.

Network	Layer	Neurons
${\mathrm{NN}}_{K}$	5	150
${\mathrm{NN}}_{P}$	5	150
${\mathrm{NN}}_{c}$	5	200
${\mathrm{NN}}_{\sigma }$	5	66

Once the model is trained, we obtain the corresponding dimensional fields as follows

$$c=c_{\text{char}}c$$

$$u=U_{\text{char}}u^{*}$$

where $c_{\text{char}}$ and $U_{\text{char}}$ are the corresponding characteristic dimensions as specified in the Supplementary Materials.

#### Training

Because MR-AIV encodes Darcy’s law directly into its architecture, training a model involves optimizing two objective functions: the advection-diffusion equation and continuity as a PDE constraint, and the data fidelity term. However, this process requires inferring the velocity field from experimental concentration data, which is inherently noisy. Additionally, the model must learn the permeability field, which spans four orders of magnitude. Without proper initialization, these factors can lead the model to become trapped in poor local minima. To mitigate this issue, we divide the training process into two stages: initialization and full training.

During the initialization stage, we initialize the network parameters $\Theta =\left\{\theta_{c},\theta_{K},\theta_{P}\right\}$ using the experimental concentration data together with the initial estimates of pressure and permeability. This stage is further divided into two main steps: first, learning the experimental concentration data and its associated noise structure by minimizing the negative log-likelihood loss (see the Supplementary Materials); and second, fitting the initial permeability estimates, followed by refining the pressure field to match the initial velocity estimates (see the Supplementary Materials).

During the training stage, we refine the initial field estimates by incorporating the physical constraints, ensuring that the model produces physically consistent solutions that also match the observed concentration data. Toward this end, we minimize a combined loss function that penalizes both data mismatch and equation residuals. Each loss term is defined as

$$L_{G}\left(X_{G},\Theta \right)=\sum_{\alpha }m_{\alpha }{\langle {\left[\lambda_{\alpha ,i}r_{\alpha }\left(x_{i}^{*},\Theta \right)\right]}^{q}\rangle }_{i}$$ (7)

where $x_{i}^{*}\in \Omega_{G}$ and G∈{D,E} indicates the loss group: data ($L_{D}$) or physics-based equation constraints ($L_{E}$). The operator ${\langle \cdot \rangle }_{i}$ denotes the mean over the training points $x_{i}^{*}=\left(t_{i}^{*},x_{i}^{*},y_{i}^{*},z_{i}^{*}\right)$ sampled from the subset $X_{G}\subset \Omega_{G}$. Sampling is performed iteratively using a probability distribution $p_{G,\alpha }$, defined using a residual-based-attention resampling (*36*, *74*) strategy as described in the Supplementary Materials. The exponent q>0 controls the smoothness of the loss function, allowing for a transition between *L*^2^ and *L*^1^ norms (with *q* = 2 and *q* = 1, respectively) during training.

Each loss group *G* targets specific physical quantities, indexed by α. For the physics-based loss (G=E), we enforce the advection-diffusion equation and conservation of mass in their residual form, corresponding to α={AD,CM} (see the Supplementary Materials). For the data loss (G=D), we constrain only the concentration field, with α={c}. We note that all governing equations and corresponding losses are evaluated in nondimensional form (see eqs. S1 to S3). The specific details of each loss subterm are described in the Supplementary Materials.

The residual $r_{\alpha }\left(x_{i}^{*},\Theta \right)=|\hat{\alpha }\left(x_{i}^{*}\right)-\alpha (x_{i}^{*},\Theta )|$ quantifies the discrepancy between the predicted value $\alpha \left(x_{i}^{*},\Theta \right)$ and the target value $\hat{\alpha }\left(x_{i}^{*}\right)$ (e.g., experimental observation or zero for the PDEs). Last, RBA weights (*49*) $\lambda_{\alpha ,i}$ are used as local multipliers to control the pointwise contribution of each residual, while global weights $m_{\alpha }$ balance the relative importance of each subcomponent α across the loss groups. This sequential training is implemented by varying these global $m_{\alpha }$ weights at each stage, which adaptively shifts the optimizer’s focus from the initial estimates to the PDE and data losses (see fig. S1). The full details of this stagewise weighting, including the specific values for each stage, are provided in Table 2. The specific formulation for the RBA weights is described in the Supplementary Materials.

**Table 2.** Comprehensive training schedule and weighting strategy. Summary of the sequential training stages. The table unifies the global balancing weights ($m_{\alpha }$), the local adaptive weights ($\lambda_{\alpha ,i}$), and the loss norm (*q*). Note that, after the concentration is initialized, the advection-diffusion (AD) term consistently uses the TD-RBA scaling $1/C\left(t_{i}\right)$. CM, conservation of mass.

Stage	Loss term	Subterm (α)	Norm (*q*)	Global ($m_{\alpha }$)	Local ($\lambda_{\alpha ,i}$)
0	Data low (eq. S11)	$K_{L}$	2	100	$\lambda_{K_{L},i}$
0	Data high (eq. S11)	$K_{H}$	2	1	$\lambda_{K_{H},i}$
1	Data (eq. S10)	$c,\sigma_{c}$ (eq. S9)	–	100	$\lambda_{c,i}$
1	Initialization (eq. S12)	*u*, *v*, *w*	2	100	$\lambda_{u,i},\lambda_{v,i},\lambda_{w,i}$
1	Equations (eq. S13)	AD (eq. S4)	2	10^−16^	$\lambda_{\text{AD},i}$
1	Equations (eq. S13)	CM (eq. S5)	2	10^−16^	$\lambda_{\text{CM},i}$
2	Equations (eq. S13)	AD (eq. S4)	2	10	$\lambda_{\text{AD},i}/C\left(t_{i}\right)$
2	Equations (eq. S13)	CM (eq. S5)	2	10^−2^	$\lambda_{\text{CM},i}$
3	Equations (eq. S13)	AD (eq. S4)	2	10	$\lambda_{\text{AD},i}/C\left(t_{i}\right)$
3	Equations (eq. S13)	CM (eq. S5)	2	10^−2^	$\lambda_{\text{CM},i}$
4	Data (eq. S14)	*c*	2	100	$\lambda_{c,i}$
4	Equations (eq. S16)	AD (eq. S4)	2	10	$\lambda_{\text{AD},i}/C\left(t_{i}\right)$
4	Equations (eq. S16)	CM (eq. S5)	2	10^−2^	$\lambda_{\text{CM},i}$
5	Data (eq. S14)	*c*	1	10	$\lambda_{c,i}$
5	Equations (eq. S16)	AD (eq. S4)	1	10	$\lambda_{\text{AD},i}/C\left(t_{i}\right)$
5	Equations (eq. S16)	CM (eq. S5)	1	10^−2^	$\lambda_{\text{CM},i}$

#### TD-RBA with resampling

A key challenge in this problem is the vast dynamic range of the concentration field, which causes the physical residuals in the loss function to vary by orders of magnitude over time. To ensure stable training, we developed TD-RBA, an optimization method that acts as a pointwise adaptive learning rate, guided directly by the physics of the advection-diffusion equation. This is achieved by introducing a time-dependent weight for the advection-diffusion loss, $\lambda_{\mathrm{AD},i}\left(t_{i}\right)=\lambda_{\mathrm{AD},i}/C\left(t_{i}\right)$, where $\lambda_{\mathrm{AD},i}$ is a standard RBA weight. The main contribution of this study is the time-dependent scaling factor, $C\left(t_{i}\right)$, which normalizes the residuals at each time point and is defined by the maximum magnitude of the advection-diffusion equation’s components

$$C\left(t_{i}^{*}\right)=\text{max}\left(|c_{x}|,|c_{y}|,|c_{z}|,|c_{t}-\frac{1}{{\mathrm{Pe}}_{g}}\nabla^{2}c|\right)$$

where all gradients are evaluated across the spatial domain at time $t_{i}^{*}$. Here, ${\mathrm{Pe}}_{g}$ is the global Péclet number obtained after nondimensionalizing our equations as described in the Supplementary Materials. Notice that this scaling is applied only to the AD residuals. This approach aims to ensure all phases of tracer transport contribute meaningfully to the solution. The full formulation, including the underlying RBA with resampling, is provided in the Supplementary Materials.

### Computational fluid dynamics validation

To evaluate the performance of MR-AIV, we conduct a comprehensive validation using high-fidelity synthetic datasets generated via the finite element method. Each simulation consists of ∼2 million tetrahedral elements (see fig. S3A). Full details of the numerical setup are provided in the Supplementary Materials.

We evaluate MR-AIV using three permeability maps to solve Darcy’s law, each yielding a distinct velocity field. To match the scale of real data, low and high permeability regions are set to 10^−6^, mm^2^ and 10^−10^, mm^2^, respectively. While all maps are predominantly binary, they differ in the sharpness and complexity of the transitions between regions. The smooth map contains gradual transitions, the sharp map features abrupt interfaces, and the “realistic” map combines sharp transitions with a more intricate spatial layout (see fig. S3B). Once the velocity fields are obtained, we solve the advection-diffusion equation to compute the corresponding concentration fields.

We use 50% of the concentration data to train our MR-AIV model following the same strategy as for the real data. Then, we evaluate performance using the relative *L*^2^ error quantified as

$${\mathrm{RL}}_{2}=\frac{∥\hat{d}\left(x\right)-d\left(x\right){∥}_{2}}{∥\hat{d}\left(x\right){∥}_{2}}=\frac{\sqrt{\sum_{i=1}^{n}{\left[\hat{d}\left(x_{i}\right)-d(x_{i},\theta )\right]}^{2}}}{\sqrt{\sum_{i=1}^{n}\hat{d}{\left(x_{i}\right)}^{2}}}$$ (8)

where $d\left(x_{i}\right)$ is the predicted value (i.e., concentration, velocity, permeability, or pressure) at point $x_{i}$.

The model successfully reconstructed the synthetic concentration fields (fig. S4), maintaining a relative *L*^2^ error below 2%. For the velocity magnitude inference (fig. S5), relative errors were 36% as detailed in table S1. These inference errors were primarily concentrated in regions of extremely low velocity, a finding corroborated by the uncertainty quantification analysis as shown in Fig. 8 and fig. S8.

To assess the directional fidelity of the inferred velocity fields, we calculate the mean angular error. This metric quantifies the average deviation in orientation between the predicted and reference velocity vectors, independent of their magnitudes. The pointwise angular error, $\theta \left(x_{i}\right)$, is computed via the cosine similarity

$$\theta \left(x_{i}\right)=\text{arccos}\left(\frac{\hat{u}\left(x_{i}\right)\cdot u\left(x_{i}\right)}{∥\hat{u}\left(x_{i}\right)∥∥u\left(x_{i}\right)∥}\right)$$ (9)

We report the arithmetic mean, $\bar{\theta }=\frac{1}{N}\sum_{i=1}^{N}\theta \left(x_{i}\right)$, in table S1. The results indicate strong directional alignment for the smooth case ($\bar{\theta }\approx 10.1{}^{\circ}$). While the error increases to 23.9° for the Realistic case, this is largely attributed to sensitivity in low-velocity regions.

Last, the results for the estimated pressure and permeability are shown in fig. S6. Notably, the predicted fields exhibit strong agreement with the reference fields, preserving key structural features and regional contrasts. However, while the spatial trends are well recovered, the absolute magnitudes of the predicted fields deviate from the ground truth due to the ill-posedness of the problem, as detailed further in the Supplementary Materials and their corresponding uncertainty quantification (fig. S9).

## Supplementary Materials

### The PDF file includes:

Supplementary Materials and Methods Figs. S1 to S9 Tables S1 to S7 Legends for movies S1 to S4 References

## References

[1] L. Xie, H. Kang, Q. Xu, M. J. Chen, Y. Liao, M. Thiyagarajan, J. O’Donnell, D. J. Christensen, C. Nicholson, J. J. Iliff, T. Takano, R. Deane, M. Nedergaard, Sleep drives metabolite clearance from the adult brain. Science 342, 373–377 (2013). 24136970 10.1126/science.1241224 PMC3880190

[2] M. K. Rasmussen, H. Mestre, M. Nedergaard, The glymphatic pathway in neurological disorders. Lancet Neurol. 17, 1016–1024 (2018). 30353860 10.1016/S1474-4422(18)30318-1 PMC6261373

[3] B. A. Plog, M. Nedergaard, The glymphatic system in central nervous system health and disease: Past, present, and future. Annu. Rev. Pathol. 13, 379–394 (2018). 29195051 10.1146/annurev-pathol-051217-111018 PMC5803388

[4] H. Mestre, T. Du, A. M. Sweeney, G. Liu, A. J. Samson, W. Peng, K. N. Mortensen, F. F. Stæger, P. A. R. Bork, L. Bashford, E. R. Toro, J. Tithof, D. H. Kelley, J. H. Thomas, P. G. Hjorth, E. A. Martens, R. I. Mehta, O. Solis, P. Blinder, D. Kleinfeld, H. Hirase, Y. Mori, M. Nedergaard, Cerebrospinal fluid influx drives acute ischemic tissue swelling. Science 367, eaax7171 (2020). 32001524 10.1126/science.aax7171 PMC7375109

[5] D. H. Kelley, J. H. Thomas, Cerebrospinal fluid flow. Annu. Rev. Fluid Mech. 55, 237–264 (2023). 39691763 10.1146/annurev-fluid-120720-011638 PMC11651633

[6] H. Mestre, J. Tithof, T. Du, W. Song, W. Peng, A. M. Sweeney, G. Olveda, J. H. Thomas, M. Nedergaard, D. H. Kelley, Flow of cerebrospinal fluid is driven by arterial pulsations and is reduced in hypertension. Nat. Commun. 9, 4878 (2018). 30451853 10.1038/s41467-018-07318-3 PMC6242982

[7] F. M. Rivas, J. Liu, B. C. Martell, D. Ting, H. Mestre, M. Nedergaard, J. Tithof, J. H. Thomas, D. H. Kelley, Surface periarterial spaces of the mouse brain are open, not porous. J. R. Soc. Interface 17, 20200593 (2020). 33171075 10.1098/rsif.2020.0593 PMC7729052

[8] A. Raghunandan, A. Ladrón-de-Guevara, J. Tithof, H. Mestre, D. Ting, M. Nedergaard, J. H. Thomas, D. H. Kelley, Bulk flow of cerebrospinal fluid observed in periarterial spaces is not an artifact of injection. eLife 10, e65958 (2021). 33687330 10.7554/eLife.65958 PMC7979157

[9] K. A. S. Boster, S. Cai, A. Ladrón-de-Guevara, J. Sun, X. Zheng, T. Du, J. H. Thomas, M. Nedergaard, G. E. Karniadakis, D. H. Kelley, Artificial intelligence velocimetry reveals in vivo flow rates, pressure gradients, and shear stresses in murine perivascular flows. Proc. Natl. Acad. Sci. U.S.A. 120, e2217744120 (2023). 36989300 10.1073/pnas.2217744120 PMC10083563

[10] J. D. Toscano, W. Chenxi, A. Ladrón-de-Guevara, D. Ting, M. Nedergaard, D. H. Kelley, G. E. Karniadakis, K. A. S. Boster, Inferring in vivo murine cerebrospinal fluid flow using artificial intelligence velocimetry with moving boundaries and uncertainty quantification. Interface Focus 14, 20240030 (2024). 39649446 10.1098/rsfs.2024.0030 PMC11621842

[11] B. A. Plog, H. Mestre, G. E. Olveda, A. M. Sweeney, H. M. Kenney, A. Cove, K. Y. Dholakia, J. Tithof, T. D. Nevins, I. Lundgaard, T. Du, D. H. Kelley, M. Nedergaard, Transcranial optical imaging reveals a pathway for optimizing the delivery of immunotherapeutics to the brain. JCI Insight 3, e120922 (2018). 30518698 10.1172/jci.insight.126138 PMC6328017

[12] A. S. Munk, W. Wang, N. B. Bèchet, A. M. Eltanahy, A. X. Cheng, B. Sigurdsson, A. Benraiss, M. A. Mäe, B. T. Kress, D. H. Kelley, C. Betsholtz, K. Møllgård, A. Meissner, M. Nedergaard, I. Lundgaard, PDGF-B is required for development of the glymphatic system. Cell Rep. 26, 2955–2969.e3 (2019). 30865886 10.1016/j.celrep.2019.02.050 PMC6447074

[13] L. M. Valnes, S. K. Mitusch, G. Ringstad, P. K. Eide, S. W. Funke, K.-A. Mardal, Apparent diffusion coefficient estimates based on 24 hours tracer movement support glymphatic transport in human cerebral cortex. Sci. Rep. 10, 9176 (2020). 32514105 10.1038/s41598-020-66042-5 PMC7280526

[14] L. A. Ray, M. Pike, M. Simon, J. J. Iliff, J. J. Heys, Quantitative analysis of macroscopic solute transport in the murine brain. Fluids Barriers CNS 18, 55 (2021). 34876169 10.1186/s12987-021-00290-z PMC8650464

[15] B. Zapf, J. Haubner, M. Kuchta, G. Ringstad, P. K. Eide, K.-A. Mardal, Investigating molecular transport in the human brain from MRI with physics-informed neural networks. Sci. Rep. 12, 15475 (2022). 36104360 10.1038/s41598-022-19157-w PMC9474534

[16] J. Oldenburg, J. Renkewitz, M. Stiehm, K.-P. Schmitz, Contributions towards data driven deep learning methods to predict steady state fluid flow in mechanical heart valves. Curr. Dir. Biomed. Eng. 7, 625–628 (2021).

[17] X. Hou, P. Guo, P. Wang, P. Liu, D. D. M. Lin, H. Fan, Y. Li, Z. Wei, Z. Lin, D. Jiang, Deep-learning-enabled brain hemodynamic mapping using resting-state fMRI. NPJ Digit. Med. 6, 116 (2023). 37344684 10.1038/s41746-023-00859-y PMC10284915

[18] D. R. Rutkowski, A. Roldán-Alzate, K. M. Johnson, Enhancement of cerebrovascular 4D flow MRI velocity fields using machine learning and computational fluid dynamics simulation data. Sci. Rep. 11, 10240 (2021). 33986368 10.1038/s41598-021-89636-z PMC8119419

[19] S. Talebi, S. Gai, A. Sossin, V. Zhu, E. Tong, M. R. K. Mofrad, Deep learning for perfusion cerebral blood flow (CBF) and volume (CBV) predictions and diagnostics. Ann. Biomed. Eng. 52, 1568–1575 (2024). 38402314 10.1007/s10439-024-03471-7 PMC11082011

[20] V. Ratner, Y. Gao, H. Lee, R. Elkin, M. Nedergaard, H. Benveniste, A. Tannenbaum, Cerebrospinal and interstitial fluid transport via the glymphatic pathway modeled by optimal mass transport. Neuroimage 152, 530–537 (2017). 28323163 10.1016/j.neuroimage.2017.03.021 PMC5490081

[21] A. F. Frangi, J. A. Schnabel, C. Davatzikos, C. Alberola-López, G. Fichtinger, *GlymphVIS: Visualizing Glymphatic Transport Pathways Using Regularized Optimal Transport* (Springer International Publishing, 2018). 10.1007/978-3-030-00928-1_95 PMC6426141 30906935

[22] S. Koundal, R. Elkin, S. Nadeem, Y. Xue, S. Constantinou, S. Sanggaard, X. Liu, B. Monte, X. Feng, W. Nostrand, M. Nedergaard, H. Lee, J. Wardlaw, H. Benveniste, A. Tannenbaum, Optimal mass transport with Lagrangian workflow reveals advective and diffusion driven solute transport in the glymphatic system. Sci. Rep. 10, 1–18 (2020). 32029859 10.1038/s41598-020-59045-9 PMC7004986

[23] X. Chen, X. Liu, S. Koundal, R. Elkin, X. Zhu, B. Monte, X. Feng, F. Dai, M. Pedram, H. Lee, J. Kipnis, A. Tannenbaum, W. E. Van Nostrand, H. Benveniste, Cerebral amyloid angiopathy is associated with glymphatic transport reduction and time-delayed solute drainage along the neck arteries. Nat. Aging 2, 214–223 (2022). 36199752 10.1038/s43587-022-00181-4 PMC9531841

[24] S. Koundal, X. Chen, Z. Gursky, H. Lee, K. Xu, F. Liang, Z. Xie, F. Xu, H.-M. Lin, W. E. Van Nostrand, X. Gu, R. Elkin, A. Tannenbaum, H. Benveniste, Divergent brain solute clearance in rat models of cerebral amyloid angiopathy and Alzheimer’s disease. iScience 27, 111463 (2024). 39720539 10.1016/j.isci.2024.111463 PMC11667077

[25] V. Vinje, B. Zapf, G. Ringstad, P. K. Eide, M. E. Rognes, K.-A. Mardal, Human brain solute transport quantified by glymphatic mri-informed biophysics during sleep and sleep deprivation. Fluids Barriers CNS 20, 62 (2023). 37596635 10.1186/s12987-023-00459-8 PMC10439559

[26] T. Bohr, P. G. Hjorth, S. C. Holst, S. Hrabětová, V. Kiviniemi, T. Lilius, I. Lundgaard, K.-A. Mardal, E. A. Martens, Y. Mori, U. V. Nägerl, C. Nicholson, A. Tannenbaum, J. H. Thomas, J. Tithof, H. Benveniste, J. J. Iliff, D. H. Kelley, M. Nedergaard, The glymphatic system: Current understanding and modeling. iScience 25, 104987 (2022). 36093063 10.1016/j.isci.2022.104987 PMC9460186

[27] L. Ray, J. J. Iliff, J. J. Heys, Analysis of convective and diffusive transport in the brain interstitium. Fluids Barriers CNS 16, 6 (2019). 30836968 10.1186/s12987-019-0126-9 PMC6402182

[28] K. E. Holter, B. Kehlet, A. Devor, T. J. Sejnowski, A. M. Dale, S. W. Omholt, O. P. Ottersen, E. A. Nagelhus, K.-A. Mardal, K. H. Pettersen, Interstitial solute transport in 3D reconstructed neuropil occurs by diffusion rather than bulk flow. Proc. Nat. Acad. Sci. U.S.A. 114, 9894–9899 (2017). 10.1073/pnas.1706942114 PMC5604020 28847942

[29] M. Keith Sharp, R. O. Carare, B. A. Martin, Dispersion in porous media in oscillatory flow between flat plates: Applications to intrathecal, periarterial and paraarterial solute transport in the central nervous system. Fluids Barriers CNS 16, 13 (2019). 31056079 10.1186/s12987-019-0132-y PMC6512764

[30] F. Romanò, V. Suresh, P. A. Galie, J. B. Grotberg, Peristaltic flow in the glymphatic system. Sci. Rep. 10, 21065 (2020). 33273489 10.1038/s41598-020-77787-4 PMC7713425

[31] R. T. Kedarasetti, P. J. Drew, F. Costanzo, Arterial vasodilation drives convective fluid flow in the brain: A poroelastic model. Fluids Barriers CNS 19, 34 (2022). 35570287 10.1186/s12987-022-00326-y PMC9107702

[32] M. M. Meerschaert, C. Tadjeran, Finite difference approximations for fractional advection–dispersion flow equations. J. Comput. Appl. Math. 172, 65–77 (2004).

[33] M. Raissi, P. Perdikaris, G. E. Karniadakis, Physics-informed neural networks: A deep learning framework for solving forward and inverse problems involving nonlinear partial differential equations. J. Comput. Phys. 378, 686–707 (2019).

[34] M. Raissi, A. Yazdani, G. E. Karniadakis, Hidden fluid mechanics: Learning velocity and pressure fields from flow visualizations. Science 367, 1026–1030 (2020). 32001523 10.1126/science.aaw4741 PMC7219083

[35] S. Cai, H. Li, F. Zheng, F. Kong, M. Dao, G. E. Karniadakis, S. Suresh, Artificial intelligence velocimetry and microaneurysm-on-a-chip for three-dimensional analysis of blood flow in physiology and disease. Proc. Natl. Acad. Sci. U.S.A. 118, e2100697118 (2021). 33762307 10.1073/pnas.2100697118 PMC8020788

[36] J. D. Toscano, T. Käufer, Z. Wang, M. Maxey, C. Cierpka, G. E. Karniadakis, AIVT: Inference of turbulent thermal convection from measured 3D velocity data by physics-informed Kolmogorov-Arnold networks. Sci. Adv. 11, eads5236 (2025). 40333963 10.1126/sciadv.ads5236 PMC12057682

[37] S. Kida, R. O. Weller, E.-T. Zhang, M. J. Phillips, F. Iannotti, Anatomical pathways for lymphatic drainage of the brain and their pathological significance. Neuropathol. Appl. Neurobiol. 21, 181–184 (1995). 7477725 10.1111/j.1365-2990.1995.tb01048.x

[38] H. Mansour, R. Azrak, J. J. Cook, K. J. Hornburg, Y. Qi, Y. Tian, R. W. Williams, F.-C. Yeh, L. E. White, G. A. Johnson, The duke mouse brain atlas: MRI and light sheet microscopy stereotaxic atlas of the mouse brain. Sci. Adv. 11, eadq8089 (2025). 40305623 10.1126/sciadv.adq8089 PMC12042906

[39] M. Moazen, A. Alazmani, K. Rafferty, Z.-J. Liu, J. Gustafson, M. L. Cunningham, M. J. Fagan, S. W. Herring, Intracranial pressure changes during mouse development. J. Biomech. 49, 123–126 (2016). 26620442 10.1016/j.jbiomech.2015.11.012

[40] E. A. Schmidt, F. Despas, A. P.-L. Traon, Z. Czosnyka, J. D. Pickard, K. Rahmouni, A. Pathak, J. M. Senard, Intracranial pressure is a determinant of sympathetic activity. Front. Physiol. 9, 11 (2018). 29472865 10.3389/fphys.2018.00011 PMC5809772

[41] G. Shen, S. Link, S. Kumar, D. M. Nusbaum, D. Y. Tse, F. Yingbin, S. M. Wu, B. J. Frankfort, Characterization of retinal ganglion cell and optic nerve phenotypes caused by sustained intracranial pressure elevation in mice. Sci. Rep. 8, 2856 (2018). 29434244 10.1038/s41598-018-21254-8 PMC5809383

[42] L. Bordoni, B. Li, S. Kura, D. A. Boas, S. Sakadžić, L. Østergaard, S. Frische, E. Gutiérrez-Jiménez, Quantification of capillary perfusion in an animal model of acute intracranial hypertension. J. Neurotrauma 38, 446–454 (2021). 32998634 10.1089/neu.2019.6901 PMC8020532

[43] K. Oshio, H. Watanabe, Y. Song, A. S. Verkman, G. T. Manley, Reduced cerebrospinal fluid production and intracranial pressure in mice lacking choroid plexus water channel Aquaporin-1. FASEB J. 19, 76–78 (2005). 15533949 10.1096/fj.04-1711fje

[44] S. Feiler, B. Friedrich, K. Schöller, S. C. Thal, N. Plesnila, Standardized induction of subarachnoid hemorrhage in mice by intracranial pressure monitoring. J. Neurosci. Methods 190, 164–170 (2010). 20457182 10.1016/j.jneumeth.2010.05.005

[45] B. Yang, Z. Zador, A. S. Verkman, Glial cell aquaporin-4 overexpression in transgenic mice accelerates cytotoxic brain swelling. J. Biol. Chem. 283, 15280–15286 (2008). 18375385 10.1074/jbc.M801425200 PMC2397463

[46] K. A. S. Boster, J. Tithof, D. D. Cook, J. H. Thomas, D. H. Kelley, Sensitivity analysis on a network model of glymphatic flow. J. R. Soc. Interface 19, 20220257 (2022). 35642425 10.1098/rsif.2022.0257 PMC9156905

[47] B. Lakshminarayanan, A. Pritzel, C. Blundell, Simple and scalable predictive uncertainty estimation using deep ensembles. Adv. Neural Inf. Proces. Syst. 30, 6405–6416 (2017).

[48] S. J. Anagnostopoulos, J. D. Toscano, N. Stergiopulos, G. E. Karniadakis, Learning in PINNs: Phase transition, total diffusion, and generalization. arXiv:2403.18494 (2024). 10.1016/j.neunet.2025.107983 PMC13444385 40884895

[49] S. J. Anagnostopoulos, J. D. Toscano, N. Stergiopulos, G. E. Karniadakis, Residual-based attention in physics-informed neural networks. Comput. Methods Appl. Mech. Eng. 421, 116805 (2024).

[50] J. D. Toscano, L.-L. Wang, G. E. Karniadakis, KKANS: Ku˙rková-Kolmogorov-Arnold networks and their learning dynamics. Neural Netw. 191, 107831 (2025). 40664158 10.1016/j.neunet.2025.107831 PMC13151308

[51] K. Shukla, J. D. Toscano, Z. Wang, Z. Zou, G. E. Karniadakis, A comprehensive and FAIR comparison between MLP and KAN representations for differential equations and operator networks. Comput. Methods Appl. Mech. Eng. 431, 117290 (2024).

[52] C. Wu, J. D. Toscano, K. Shukla, Y. Chen, A. Shahmohammadi, E. Raymond, T. Toupy, N. Nazemifard, C. Papageorgiou, G. E. Karniadakis, FMEnets: Flow, material, and energy networks for non-ideal plug flow reactor design. arXiv:2505.20300 (2025). 10.1016/j.ces.2025.122348 PMC13456525 42578006

[53] J. J. Iliff, M. Wang, B. A. Yang Liao, W. P. Plog, G. A. Gundersen, H. Benveniste, G. E. Vates, R. Deane, S. A. Goldman, E. A. Nagelhus, M. Nedergaard, A paravascular pathway facilitates CSF flow through the brain parenchyma and the clearance of interstitial solutes, including amyloid β. Sci. Transl. Med. 4, 147ra111 (2012). 10.1126/scitranslmed.3003748 PMC3551275 22896675

[54] J. V. George, K. J. Hornburg, A. Merrill, E. Marvin, K. Conrad, K. Welle, R. Gelein, D. Chalupa, U. Graham, G. Oberdörster, G. A. Johnson, D. A. Cory-Slechta, M. Sobolewski, Brain iron accumulation in neurodegenerative disorders: Does air pollution play a role? Part. Fibre Toxicol. 22, 9 (2025). 40312348 10.1186/s12989-025-00622-z PMC12046710

[55] R. D. Penn, A. Linninger, The physics of hydrocephalus. Pediatr. Neurosurg. 45, 161–174 (2009). 19440003 10.1159/000218198

[56] Y. Guo, K. Quirk, D. H. Kelley, J. H. Thomas, Advection and diffusion in perivascular and extracellular spaces in the brain. J. R. Soc. Interface 22, 20250010 (2025). 40393523 10.1098/rsif.2025.0010 PMC12092104

[57] B. Bedussi, M. Almasian, J. de Vos, E. Van Bavel, E. N. Bakker, Paravascular spaces at the brain surface: Low resistance pathways for cerebrospinal fluid flow. J. Cereb. Blood Flow Metab. 38, 719–726 (2018). 29039724 10.1177/0271678X17737984 PMC5888857

[58] P. J. Basser, Interstitial pressure, volume, and flow during infusion into brain tissue. Microvasc. Res. 44, 143–165 (1992). 1474925 10.1016/0026-2862(92)90077-3

[59] P. J. Drew, A. Y. Shih, D. Kleinfeld, Fluctuating and sensory-induced vasodynamics in rodent cortex extend arteriole capacity. Proc. Natl. Acad. Sci. U.S.A. 108, 8473–8478 (2011). 21536897 10.1073/pnas.1100428108 PMC3100929

[60] S. J. van Veluw, S. S. Hou, M. Calvo-Rodriguez, M. Arbel-Ornath, A. C. Snyder, M. P. Frosch, S. M. Greenberg, B. J. Bacskai, Vasomotion as a driving force for paravascular clearance in the awake mouse brain. Neuron 105, 549–561.e5 (2020). 31810839 10.1016/j.neuron.2019.10.033 PMC7028316

[61] X. Wang, J. A. Padawer-Curry, A. R. Bice, B. Kim, Z. P. Rosenthal, J.-M. Lee, M. S. Goyal, S. L. Macauley, A. Q. Bauer, Spatiotemporal relationships between neuronal, metabolic, and hemodynamic signals in the awake and anesthetized mouse brain. Cell Rep. 43, 114723 (2024). 39277861 10.1016/j.celrep.2024.114723 PMC11523563

[62] G. A. Johnson, Y. Tian, D. G. Ashbrook, G. P. Cofer, J. J. Cook, J. C. Gee, A. Hall, K. Hornburg, C. C. Kaczorowski, Y. Qi, F.-C. Yeh, N. Wang, L. E. White, R. W. Williams, Merged magnetic resonance and light sheet microscopy of the whole mouse brain. Proc. Natl. Acad. Sci. U.S.A. 120, e2218617120 (2023). 37068254 10.1073/pnas.2218617120 PMC10151475

[63] X. Han, S. Maharjan, J. Chen, Y. Zhao, Y. Qi, L. E. White, G. Allan Johnson, N. Wang, High-resolution diffusion magnetic resonance imaging and spatial-transcriptomic in developing mouse brain. Neuroimage 297, 120734 (2024). 39032791 10.1016/j.neuroimage.2024.120734 PMC11377129

[64] E. H. Stanton, N. D. Å. Persson, R. S. Gomolka, T. Lilius, B. Sigurdsson, H. Lee, A. L. R. Xavier, H. Benveniste, M. Nedergaard, Y. Mori, Mapping of csf transport using high spatiotemporal resolution dynamic contrast-enhanced mri in mice: Effect of anesthesia. Magn. Reson. Med. 85, 3326–3342 (2021). 33426699 10.1002/mrm.28645

[65] R. S. Gomolka, L. M. Hablitz, H. Mestre, M. Giannetto, D. Ting, N. L. Hauglund, L. Xie, W. Peng, P. M. Martinez, M. Nedergaard, Y. Mori, Loss of aquaporin-4 results in glymphatic system dysfunction via brain-wide interstitial fluid stagnation. eLife 12, e82232 (2023). 36757363 10.7554/eLife.82232 PMC9995113

[66] T. D. Nevins, D. H. Kelley, Front tracking for quantifying advection-reaction-diffusion. Chaos 27, 043105–043110 (2017). 28456164 10.1063/1.4979668

[67] T. D. Nevins, D. H. Kelley, Front tracking velocimetry in advection-reaction-diffusion systems. Chaos 28, 043122–043111 (2018). 31906630 10.1063/1.5020055

[68] G. Ringstad, L. M. Valnes, A. M. Dale, A. H. Pripp, S.-A. S. Vatnehol, K. E. Emblem, K.-A. Mardal, P. K. Eide, Brain-wide glymphatic enhancement and clearance in humans assessed with mri. JCI Insight 3, 7 (2018). 10.1172/jci.insight.121537 PMC6124518 29997300

[69] P. K. Eide, G. Ringstad, Functional analysis of the human perivascular subarachnoid space. Nat. Commun. 15, 2001 (2024). 38443374 10.1038/s41467-024-46329-1 PMC10914778

[70] J. D. Toscano, V. Oommen, A. J. Varghese, Z. Zou, N. A. Daryakenari, W. Chenxi, G. E. Karniadakis, From pinns to pikans: Recent advances in physics-informed machine learning. arXiv:2410.13228 [cs.LG] (2024). 10.1007/s44379-025-00015-1 PMC13290273 42344579

[71] S. Wang, S. Sankaran, H. Wang, P. Perdikaris, An expert’s guide to training physics-informed neural networks. arXiv:2308.08468 [cs.LG] (2023).

[72] T. Salimans, D. P. Kingma, Weight normalization: A simple reparameterization to accelerate training of deep neural networks. Adv. Neural Inf. Proces. Syst. 29, 901–909 (2016).

[73] S. Wang, B. Li, Y. Chen, P. Perdikaris, Piratenets: Physics-informed deep learning with residual adaptive networks. J. Mach. Learn. Res. 25, 19707–19757 (2024).

[74] J. D. Toscano, D. T. Chen, V. Oommen, J. Darbon, G. E. Karniadakis, A variational framework for residual-based adaptivity in neural PDE solvers and operator learning. arXiv:2509.14198 [cs.LG] (2025). 10.1038/s44387-026-00084-4 PMC12967272 41804505

[75] J. Quiñonero-Candela, I. Dagan, B. Magnini, F. D’Alché-Buc, Machine Learning Challenges: Evaluating Predictive Uncertainty, Visual Object Classification, and Recognizing Textual Entailment, First Pascal Machine Learning Challenges Workshop, MLCW 2005, Southampton, UK, April 11-13, 2005, Revised Selected Papers (Springer, 2006), vol. 3944, 10.1007/11736790.

[76] I. Loshchilov, F. Hutter, Decoupled weight decay regularization. arXiv:1711.05101 [cs.LG] (2017).

[77] L. D. McClenny, UlissesMBraga-Neto., Self-adaptive physics-informed neural networks. J. Comput. Phys. 474, 111722 (2023).

[78] J. D. Toscano, D. T. Chen, G. E. Karniadakis, ATHENA: Agentic team for hierarchical evolutionary numerical algorithms. arXiv:2512.03476 [cs.LG] (2025).

[79] W. Chenxi, M. Zhu, Q. Tan, Y. Kartha, L. Lu, A comprehensive study of non-adaptive and residual-based adaptive sampling for physics-informed neural networks. Comput. Methods Appl. Mech. Eng. 403, 115671 (2023).

[80] A. A. Linninger, M. Xenos, D. C. Zhu, M. B. R. Somayaji, S. Kondapalli, R. D. Penn, Cerebrospinal fluid flow in the normal and hydrocephalic human brain. IEEE Trans. Biomed. Eng. 54, 291–302 (2007). 17278586 10.1109/TBME.2006.886853

[81] T. Koch, K.-A. Mardal, Estimation of fluid flow velocities in cortical brain tissue driven by the microvasculature. Interface Focus 15, 20240042 (2025). 40191021 10.1098/rsfs.2024.0042 PMC11969191

[82] J. P. Kinney, J. Spacek, T. M. Bartol, C. L. Bajaj, K. M. Harris, T. J. Sejnowski, Extracellular sheets and tunnels modulate glutamate diffusion in hippocampal neuropil. J. Comp. Neurol. 521, 448–464 (2013). 22740128 10.1002/cne.23181 PMC3540825

[83] A. F. Psaros, X. Meng, Z. Zou, L. Guo, G. E. Karniadakis, Uncertainty quantification in scientific machine learning: Methods, metrics, and comparisons. J. Comput. Phys. 477, 111902 (2023).

[84] Z. Zou, X. Meng, G. E. Karniadakis, Uncertainty quantification for noisy inputs-outputs in physics-informed neural networks and neural operators arXiv:2311.11262 [cs.LG] (2023).

[85] Z. Zou, X. Meng, A. F. Psaros, G. E. Karniadakis, NeuralUQ: A comprehensive library for uncertainty quantification in neural differential equations and operators. SIAM Rev. 66, 161–190 (2024).

