# Supplementary material for: MR-AIV reveals in vivo brain-wide fluid flow with physics-informed AI
Source: Sci Adv. 2026 May 27;12(22):eaeb0404. doi: 10.1126/sciadv.aeb0404 (PMC13215179; doi:10.1126/sciadv.aeb0404)
Supplement: Supplementary file 1 — Supplementary Materials and Methods Figs. S1 to S9 Tables S1 to S7 Legends for movies S1 to S4 References [file sciadv.aeb0404_sm.pdf]

Science Advances
AAAS

Supplementary Materials for

MR-AIV reveals in vivo brain-wide fluid flow with physics-informed AI

Juan Diego Toscano *et al.*

Corresponding author: George Em Karniadakis, george_karniadakis@brown.edu;
Kimberly A. S. Boster, kboster@ur.rochester.edu; Douglas H. Kelley, d.h.kelley@rochester.edu

Sci. Adv. **12**, eaeb0404 (2026)
DOI: 10.1126/sciadv.aeb0404

The PDF file includes:

Supplementary Materials and Methods
Figs. S1 to S9
Tables S1 to S7
Legends for movies S1 to S4
References

Other Supplementary Material for this manuscript includes the following:

Movies S1 to S4

Materials and Methods

Non-dimensionalization

The concentration spans over four orders of magnitude, the permeability over four, and the velocity over five. To enable stable training and prevent numerical instabilities, it is therefore essential to non-dimensionalise these quantities. We define the following characteristic scales:

$$
\begin{aligned}
L_{\text{char}} &= \frac{H}{50}, \\
U_{\text{char}} &= U_0, \\
t_{\text{char}} &= \frac{L_{\text{char}}}{U_{\text{char}}}, \\
\kappa_{\text{char}} &= \kappa_0, \\
P_{\text{char}} &= \frac{U_{\text{char}}\,\mu\,L_{\text{char}}}{\kappa_{\text{char}}},
\end{aligned}
$$

where $H = 17$ mm approximates the maximum length of the brain of mouse 1, and $U_0 = 0.1$ mm/min corresponds to the expected velocity obtained from our front-tracking analysis. The characteristic permeability $\kappa_0 = 10^{-8}$ mm^2 is set to one hundredth of the maximum permeability guess.

The physical quantities are then non-dimensionalised as

$$
\begin{aligned}
x^* &= \frac{\mathrm{x}}{L_{\text{char}}}, \\
t^* &= \frac{\mathrm{t}}{t^*_{\text{char}}}, \\
\boldsymbol{u}^* &= \frac{\mathbf{u}}{U_{\text{char}}}, \\
c &= \frac{SER}{c_{\text{char}}},
\end{aligned}
$$

where t denotes the dimensional time, $\mathrm{x}, \mathrm{y}, \mathrm{z}$ are the dimensional spatial coordinates, and $\mathbf{u} = (\mathrm{u}, \mathrm{v}, \mathrm{w})$ represents the corresponding dimensional velocity components. The characteristic concentration $c_{\text{char}} = \frac{\max SER}{100}$ is chosen such that the non-dimensional concentration falls within $c \in [0, 100]$, and is therefore mouse-independent.

Finally, the non-dimensionalised pressure P^* and permeability K^* are defined as:

$$K^* = \frac{\kappa}{\kappa_{\text{char}}},$$
$$P^* = \frac{p}{P^*_{\text{char}}},$$

where κ and p denote the dimensional permeability and pressure field, respectively.

Using these non-dimensional quantities, we rewrite the governing equations as:

$$\boldsymbol{u}^* = -K^* \boldsymbol{\nabla} P^*, \tag{S1}$$

$$c_t + (\boldsymbol{u}^* \cdot \boldsymbol{\nabla})c = \frac{1}{Pe_g} \nabla^2 c, \tag{S2}$$

$$\boldsymbol{\nabla} \cdot \boldsymbol{u}^* = 0, \tag{S3}$$

where $Pe_g = U_{\text{char}} L_{\text{char}}/D = 2.361$ is the global Péclet number, with $D = 2.4 \times 10^{-4}$ mm^2/s denoting the diffusivity.

Equation Residuals

To enforce the governing equations within the PIML framework, we write them in residual form. The residuals used to constrain the non-dimensional advection-diffusion equation and the conservation of mass are defined as:

$$r_{\text{AD}}(\boldsymbol{x}^*, t^*) = c_t + u^* c_x + v^* c_y + w^* c_z - \frac{1}{Pe_g}(c_{xx} + c_{yy} + c_{zz}), \tag{S4}$$

$$r_{\text{CM}}(\boldsymbol{x}^*) = u^*_x + v^*_y + w^*_z \tag{S5}$$

where the subscript denotes differentiation with respect to the corresponding variable (e.g., $c_t = c_{t^*}$, $c_x = c_{x^*}$). All spatial and temporal derivatives are computed using automatic differentiation. Note that Darcy's law is not included as a residual term, as it is directly encoded into the model architecture.

MR-AIV framework

Denoising Module via Negative Log-likelihood Following (*10*), we use the negative log-likelihood (NLL) (*47, 76*)in PIML to explicitly model the aleatoric uncertainty (uncertainty due to

noise) in the concentration data. Specifically, we assume that the observed concentration field c_{obs} can be decomposed into a noise-free prediction $\bar{c}$ and additive Gaussian noise ϵ with zero mean and with spatially and temporally varying standard deviation σ_c, such that

$$c_{\text{obs}} = \bar{c} + \epsilon, \quad \epsilon \sim \mathcal{N}(0, \sigma_c^2). \tag{S6}$$

In this study, we model both the mean concentration field $\bar{c}$ and the corresponding standard deviation σ_c using two separate neural networks, with parameters $\boldsymbol{\theta}_c$ and $\boldsymbol{\theta}_\sigma$, respectively. The mean field $\bar{c}(\boldsymbol{\theta}_c, \boldsymbol{x}^*)$ provides a denoised estimate of the concentration, while $\sigma_c(\boldsymbol{\theta}_\sigma, \boldsymbol{x}^*)$ captures the spatially and temporally varying uncertainty, where $\boldsymbol{x}^* = (t^*, x^*, y^*, z^*)$ are the non-dimensional inputs.

Following previous studies (*36*), we bound the inferred standard deviation as

$$\sigma_c(\boldsymbol{x}^*, \boldsymbol{\theta}_\sigma) = 10 \cdot g(NN_\sigma(\boldsymbol{x}^*, \boldsymbol{\theta}_\sigma)) + \sigma_0, \quad \text{with } \sigma_0 = 0.01, \tag{S7}$$

where $g : \mathbb{R} \to (0, 1)$ is the sigmoid function:

$$g(x) = \frac{1}{1 + e^{-x}}, \tag{S8}$$

which ensures that $\sigma_c(\boldsymbol{x}) \in (\sigma_0, 10 + \sigma_0)$. This bounding is crucial for both physical plausibility, as the uncertainty σ_c cannot be negative, and numerical stability, as it prevents potential division-by-zero in the Negative Log-Likelihood loss function (equation S9) by ensuring σ_c remains strictly positive. The negative log-likelihood (NLL) loss is then defined as:

$$NLL(\boldsymbol{\theta}_c, \boldsymbol{\theta}_\sigma, \boldsymbol{x}^*) = \frac{\log\left[\sigma_c^2(\boldsymbol{\theta}_\sigma, \boldsymbol{x}^*)\right]}{2} + \frac{(c_{\text{obs}}(\boldsymbol{x}^*) - \bar{c}(\boldsymbol{\theta}_c, \boldsymbol{x}^*))^2}{2\sigma_c^2(\boldsymbol{\theta}_\sigma, \boldsymbol{x}^*)} + C_\sigma, \tag{S9}$$

where the constant $C_\sigma = -\log(\sigma_0^2)/2$ normalizes the metric such that the NLL is zero when the residual vanishes and $\sigma_c = \sigma_0$. The denoising loss $\mathcal{L}_N$ learns both the noise structure and the clean concentration field by penalizing the mismatch between the reconstructed mean concentration $\bar{c}$ and the experimental observations c_{obs}. It is explicitly defined as

$$\mathcal{L}_N(X_D, \boldsymbol{\theta}_c, \boldsymbol{\theta}_\sigma) = \left\langle \lambda_{c,i}^2 \, NLL(\boldsymbol{\theta}_c, \boldsymbol{\theta}_\sigma, \boldsymbol{x}_i^*) \right\rangle_i, \quad \text{where } \boldsymbol{x}_i^* \in \Omega_D. \tag{S10}$$

Here, $\langle\cdot\rangle_i$ denotes the mean operator over the batch X_D selected from the data domain Ω_D. The residual-based attention (RBA) weights $\lambda_{c,i}$ adjust the point-wise contribution of the residuals, enabling uniform convergence.

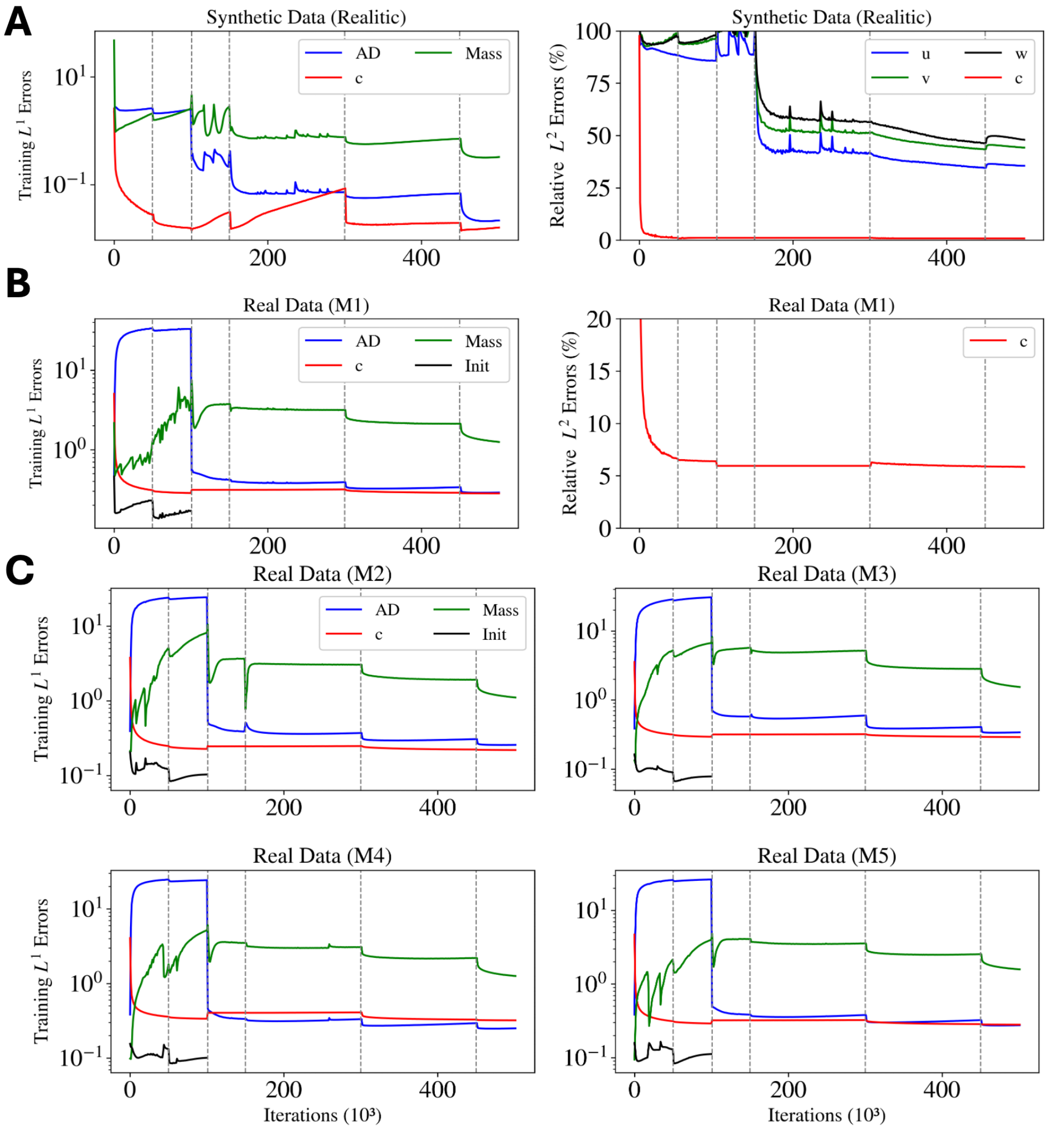

Figure S1: **Sequential Training Dynamics of MR-AIV** Training error metrics across sequential stages. Left column: Training L^1 Errors for advection-diffusion (AD, blue), conservation of mass (Mass, green), concentration (c, red), and initial estimates (Init, black). Right column: Relative L^2 Error. **(A)** Dynamics for the 'Realistic' synthetic dataset. **(B)** Dynamics for the *in vivo* Mouse 1 (M1) dataset. **(C)** L^1 error metrics for the remaining *in vivo* datasets (M2-M5). The plots show the sequential strategy: first, the initialization error (Init) is minimized while PDE errors (AD, Mass) are high. This phase handles signal denoising and field initialization. Subsequently, the initialization constraint is removed (black line terminates), and the AD and Mass errors are progressively reduced as physical constraints are enforced. Across all datasets, the Mass error metric is consistently 10 to 100 times higher than the Advection-Diffusion (AD) error.

Sequential Training

Initialization During this stage, we initialize the network parameters $\mathbf{\Theta}$ using concentration measurements along with the initial estimates of pressure and permeability. This stage is further divided into two steps: first, fitting the initial concentration and permeability estimates, and second, refining the pressure to match the initial velocity estimates (See Figure S1).

Step 0: We initialize the parameters $\boldsymbol{\theta}_K$ of the permeability network NN_K by training the model to match the initial permeability estimates. To address the distinct scales in the domain, we minimize the first initialization loss $\mathcal{L}_I^{(1)}$, defined as:

$$\mathcal{L}_I^{(1)}(X_I, \boldsymbol{\theta}_K) = \sum_{\alpha} m_\alpha \left\langle \left[\lambda_{\alpha,i}\, r_{K,i}(\boldsymbol{x}_i, \boldsymbol{\theta}_K)\right]^2 \right\rangle_i , \tag{S11}$$

where $\alpha \in \{K_{\text{high}}, K_{\text{low}}\}$ indicates the permeability region (high or low). The operator $\langle\cdot\rangle_i$ denotes the mean over the training points sampled from the corresponding subset $X_\alpha \subset X_I$. The weights m_α and $\lambda_{\alpha,i}$ correspond to the global and local balancing weights for each region, respectively (see Table 2). The term $r_{K,i} = |K(\boldsymbol{x}_i, \boldsymbol{\theta}_K) - \hat{K}_i|$ is the residual (point-wise error) between the prediction and the initial guess at location $\boldsymbol{x}_i$. We initialize the permeability network for $N_{s_0} = 500000$ ADAMw (*77*) iterations.

Step 1: We initialize the parameters of the concentration network $\boldsymbol{\theta}_c$ using our denoising module, which simultaneously recovers a clean concentration field and estimates the noise distribution. During this step, the denoising loss $\mathcal{L}_N$ is introduced to enable the learning of both the noise structure and the clean concentration by minimizing the negative log-likelihood of the experimental concentration data (Equation S9).

Concurrently, since the permeability was initialized in the previous step, we fix $\boldsymbol{\theta}_K$ and proceed to initialize the parameters of the pressure network $\boldsymbol{\theta}_P$ by fitting the initial velocity estimates obtained from the front-tracking analysis. Constraining the velocity in this way also implicitly constrains the pressure network through Darcy's law, since the pressure gradients govern the velocity field. The corresponding initialization loss is defined as:

$$\mathcal{L}_I^{(2)}(X_I, \boldsymbol{\theta}_P) = \sum_{\alpha} m_\alpha \left\langle \left[\lambda_{\alpha,i}\, r_\alpha(\boldsymbol{x}_i, \boldsymbol{\theta}_P)\right]^q \right\rangle_i , \tag{S12}$$

where $\alpha = \{u, v, w\}$ indexes the velocity components, $r_\alpha(\boldsymbol{x}_i, \boldsymbol{\theta}_P)$ is the residual between the inferred and initial velocity estimates for each component, m_α is the global weight balancing the contribution

of each sub-term, and $\lambda_{\alpha,i}$ is the corresponding local weight. Note that the symbol m_α consistently denotes global weights across all loss terms, though their specific values vary by training stage (see Table 2). We initialize the concentration and pressure for $N_{s_1} = 100000$ ADAMw (*77*) iterations.

Filtering Once we obtain the concentration field from the denoising module, the relatively high spatial resolution allows us to approximate the true gradients using automatic differentiation. Figure S2(A) shows the time evolution of the concentration gradients c_x, c_y, c_z, and $c_t - \frac{1}{Pe_g}\nabla^2 c$. The results indicate that the maximum values of these gradients exhibit high fluctuations, which may be associated with measurement noise. Moreover, the gradient magnitudes vary substantially across times, spanning several orders of magnitude. This variability highlights a fundamental challenge for stable velocity inference. Therefore, under the assumption that the concentration gradients c_x, c_y, c_z, and $c_t - \frac{1}{Pe}\nabla^2 c$ follow a log-normal distribution, we filter the data by discarding points that fall outside three standard deviations from the mean in the log-scaled domain. The mean and standard deviation of the log-scaled gradients are computed as follows:

$$\mu_g(x) = \frac{1}{N_d}\sum_{i=1}^{N_d} \log g_i,$$

$$\sigma_g(x) = \sqrt{\frac{1}{N_d}\sum_{i=1}^{N_d} \left(\log g_i - \mu_g(x)\right)^2},$$

where $g \in \{c_x, c_y, c_z, c_t - \frac{1}{Pe}\nabla^2 c\}$ and N_d denotes the number of data points at a given time. Accordingly, we discard data points where any of the gradients lie outside the range $\mu_g \pm 3\sigma_g$.

Additionally, analysis of the synthetic data (see Figure S4(C)) shows that concentration values below 10^{-2} tend to diverge from the target. To avoid introducing unreliable supervision, we also discard concentration values below 10^{-1} when learning the velocity field. We emphasize that these low-concentration regions provide minimal information for velocity reconstruction, and their exclusion prevents the propagation of weak or ambiguous signals into the PDE-constrained learning stage. Notably, this filtering step is applied exclusively during velocity inference; the concentration model is trained using unfiltered concentration data.

Training **Step 2** We begin the training stage by refining the initial pressure field. During this step, we fix the concentration and permeability parameters $\boldsymbol{\theta}_c$ and $\boldsymbol{\theta}_K$ learned in the initialization

stage, and update only the pressure parameters $\boldsymbol{\theta}_P$. Pressure refinement is performed by enforcing the physical laws, aiming to minimize the advection-diffusion and conservation of mass residuals through the equation loss:

$$\mathcal{L}_E^{(1)}(X_E, \boldsymbol{\theta}_P) = \sum_{\alpha} m_\alpha \left\langle \left[\lambda_{\alpha,i}\, r_\alpha(t_i, \boldsymbol{x}_i, \boldsymbol{\theta}_P)\right]^q \right\rangle_i, \tag{S13}$$

where $\alpha = \{\mathrm{AD}, \mathrm{CM}\}$ indexes the advection-diffusion and conservation of mass equation residuals, respectively. As in the previous cases, m_α is the global weight balancing the contribution of each term, and $\lambda_{\alpha,i}$ is the corresponding residual-based attention (RBA) weight. In this step, we use $q = 2$, inducing a squared norm appropriate for the initial training stage. Our analysis of the concentration revealed that the concentration and its gradients have a fast decay after the first few minutes of an experiment (see Figure S2(A)). Therefore, we further split the time domain into three intervals and apply three separate losses to aid the model to learn the information from the low concentration gradients. We train the pressure network for $N_{s2} = 50000$ ADAMw (*77*) iterations.

Step 3 After reducing the advection-diffusion residuals through pressure refinement, we proceed to jointly refine the permeability field. At this stage the pressure and permeability parameters $\boldsymbol{\theta}_K$, and $\boldsymbol{\theta}_P$ are updated following Equation S13. We train the pressure and permeability networks for $N_{s3} = 150000$ ADAMw (*77*) iterations.

Step 4 At this stage, all network parameters, including $\boldsymbol{\theta}_c$, $\boldsymbol{\theta}_K$, and $\boldsymbol{\theta}_P$, are updated by minimizing a combined loss that balances both the equation residuals and the mismatch with the experimental concentration data. The data loss $\mathcal{L}_D$, which constrains only the concentration field, is defined as:

$$\mathcal{L}_D(X_D, \boldsymbol{\theta}_c) = m_c \left\langle \left[\lambda_{c,i}\, r_c(t_i, \boldsymbol{x}_i, \boldsymbol{\theta}_c)\right]^q \right\rangle_i, \tag{S14}$$

where $r_c(\boldsymbol{x}_i, \boldsymbol{\theta}_c) = |\bar{c}(\boldsymbol{x}_i, \boldsymbol{\theta}_c) - \hat{c}_i|$ is the residual between the reconstructed mean concentration and the experimental observation $\hat{c}_i$ at location $\boldsymbol{x}_i$, m_c is the global weight for the concentration term, $\lambda_{c,i}$ is the corresponding local weight, and $q = 2$ is the exponent inducing a quadratic loss. The combined loss for this full training stage is given by:

$$\mathcal{L} = \mathcal{L}_E + \mathcal{L}_D, \tag{S15}$$

where $\mathcal{L}_D$ is the data loss penalizing the mismatch with the experimental concentration data (see Equation S14) and $\mathcal{L}_E$ is the equation loss enforcing the advection-diffusion and conservation of

mass constraints:

$$\mathcal{L}_E(X_E, \mathbf{\Theta}) = \sum_\alpha m_\alpha \left\langle \left[\lambda_{\alpha,i}\, r_\alpha(t_i, \boldsymbol{x}_i, \mathbf{\Theta})\right]^q \right\rangle_i, \tag{S16}$$

Notice that the main difference with equation S13 is that we are now optimizing all the parameters $\mathbf{\Theta} = \{\boldsymbol{\theta}_P, \boldsymbol{\theta}_c, \boldsymbol{\theta}_K\}$.

We train the pressure, permeability, and concentration networks for $N_{s4} = 150000$ ADAMw (*77*) iterations.

Step 5 Finally, since a quadratic loss ($q = 2$) tends to over-smooth the loss landscape, especially in the later stages of training, we include a refinement stage that uses $q = 1$. This choice promotes sharper gradients and improves the model's ability to capture fine-scale details. This strategy was introduced in (*36*), where it was experimentally shown to improve the performance of PIML models. We train the pressure, permeability, and concentration networks for $N_{s5} = 50000$ ADAMw (*77*) iterations.

Notice that the final stages correct the initial field estimates and ensure that the model produces physically consistent solutions that also match the observed concentration data.

Residual-Based Attention Methods

Residual-Based Attention weights (RBA) One of the main challenges in training neural networks is that residuals (i.e., point-wise errors) may be overlooked when computing a cumulative loss function (*48, 78*). To address this issue, we employ residual-based attention (RBA) (*49*) as local weights ($\lambda_{\alpha,i}$), which help balance the point-wise contribution of each residual term α. RBA acts as an attention mechanism, guiding the optimizer to focus on spatiotemporal regions where the residuals remain high (*48, 49, 75*). The update rule for an RBA weight $\lambda_{\alpha,i}$, associated with loss term α and point x_i, is based on the exponentially weighted moving average of the residuals:

$$\lambda_{\alpha,i}^{(k+1)} \leftarrow \gamma \lambda_{\alpha,i}^{(k)} + \eta \frac{r_{\alpha,i}^{(k)}}{\|\boldsymbol{r}_\alpha^{(k)}\|_\infty} \tag{S17}$$

where $r_{\alpha,i}^{(k)}$ is the residual for loss term α at point i, $\|\boldsymbol{r}_\alpha^{(k)}\|_\infty$ is the maximum residual at iteration k, η is the learning rate for the weights, and γ is a memory term.

Residual-Based Attention with Resampling (RBA-R) since RBA weights are updated based on the residuals computed at each specific iteration, inconsistencies can arise for large data sets trained in batches. To mitigate this issue, we leverage the RBA weights to resample critical points directly (*36*). Because the weights contain historical information about regions with persistent high error, they can be used to construct a sampling importance map q_α which is subsequently normalized to a probability distribution p_α:

$$q_\alpha^{(k+1)}(\boldsymbol{x}) = \frac{(\boldsymbol{\lambda}_\alpha^{(k)})^\nu}{\mathbb{E}[(\boldsymbol{\lambda}_\alpha^{(k)})^\nu]} + c, \tag{S18}$$

$$p_\alpha^{(k+1)}(\boldsymbol{x}_i) = \frac{q_\alpha^{(k+1)}(\boldsymbol{x}_i)}{\sum_j q_\alpha^{(k+1)}(\boldsymbol{x}_j)}. \tag{S19}$$

Here, $(\boldsymbol{\lambda}_\alpha^{(k)})^\nu$ is the vector of RBA weights raised to a power $\nu = 2$, and $c = 0.5$ is a scalar ensuring all points retain a nonzero sampling probability. We utilize these specific values ($\nu = 2, c = 0.5$) following the original RBA-R (*36*) and RAD (*79*) formulations without additional tuning.

These adaptive strategies are derived from the variational framework established in (*74*). This framework formally connects them to primal optimization objectives by solving a dual problem. The specific methods used here correspond to the linear/quadratic potential case ($\Phi(x) = x^2 + 1$), which targets variance reduction of the loss estimator. This, in turn, reduces the discretization error and enhances learning dynamics by improving the gradient's signal-to-noise ratio.

Time-dependent RBA (TD-RBA) In our framework, we apply RBA-R to all loss terms. However, the advection-diffusion equation presents an additional critical challenge: as shown in Figure S2, the concentration gradients vary by several orders of magnitude over time. This variability means that time points with large gradients can disproportionately dominate the optimization process, preventing the model from learning from the weaker signals present at other times.

To address this imbalance, we introduced our novel optimization method, Time-dependent RBA (TD-RBA). The core idea is to introduce a time-dependent scaling factor, $C(t)$, to ensure that the residuals at all times contribute equally during training. The modified loss for the advection-diffusion equation takes the form:

$$\mathcal{L}_{AD}(X_E, \boldsymbol{\Theta}) = m_{AD} \left\langle \left[\lambda_{AD,i}(t_i)\, r_\alpha(t_i^*, \boldsymbol{x}_i^*, \boldsymbol{\Theta})\right]^q \right\rangle_i, \tag{S20}$$

where the TD-RBA weight is $\lambda_{AD,i}(t_i) = \frac{\lambda_{AD,i}}{C(t_i)}$. The scaling factor $C(t_i)$ is defined by the maximum magnitude of the advection-diffusion equation's components across the spatial domain Ω_x at time t_i^*:

$$C(t_i^*) = \max_{\Omega_x} \left(|c_x|, |c_y|, |c_z|, |c_t - \frac{1}{Pe_g}\nabla^2 c| \right).$$

Since this scaling factor is computed from the clean concentration field (which is learned in an early training stage), it is calculated only once. As shown in Figure S2B, this rescaling successfully maintains the maximum gradient values within a stable range across all times. Our ablation study (Table S6) demonstrates that this physics-guided normalization is critical for reducing modeling errors.

Numerical Simulations for Synthetic Data

To generate the synthetic data, we used the finite element method code COMSOL Multiphysics 6.2 to perform the numerical simulations. The brain is a porous and elastic medium, where the elastic medium consists of the cells and the extracellular matrix (hyperelastic and viscoelastic materials may also be considered). However, for modeling fluid flow through brain tissue, a Darcy flow model is typically sufficient, as suggested by previous research (for example, (*80*) and (*81*)). To further validate this approach, we performed simulations of interstitial flow within 3D electron microscope reconstructions of hippocampal tissue from (*82*), comparing rigid and elastic material properties for solid domains. The results showed that solid deformation is negligible and the computed permeabilities in brain tissue are nearly identical.

The simulations were performed in two steps: the first step was solving for the fluid velocity field, and the second step was calculating the concentration by solving the advection-diffusion equation. The brain was considered to be a porous medium in the simulations, and the fluid motion in the domain was governed by Darcy's law and continuity; see equations 3 and 2, respectively. In all simulations, we used $\mu = 0.7 \times 10^{-3}$ Pa · s, the viscosity of water at 37°C.

The basic equation governing the concentration $C(\mathbf{x}, t)$ of a passive solute in the brain is the *advection-diffusion equation* (see equation 1). We first validated our numerical methods by comparing the numerical results with analytical solutions of one-dimensional advection-diffusion problems, obtaining good agreement. Mesh sensitivity studies were also performed to ensure that

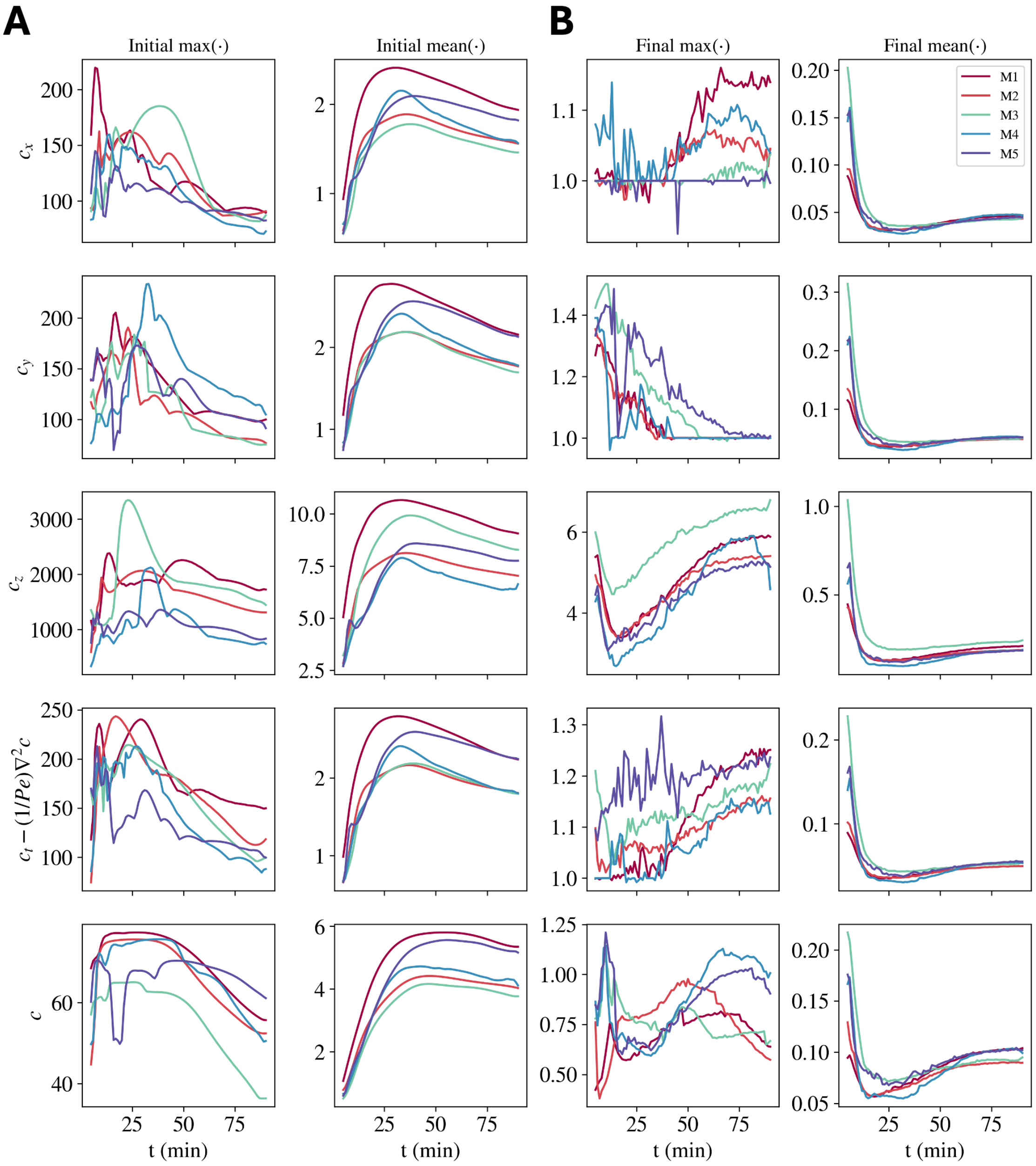

Figure S2: **Effect of filtering and time-dependent residual-based attention (TD-RBA) scaling on the concentration gradients.** (A) Time evolution of the maximum and spatially-averaged concentration gradients before filtering and scaling: c_x, c_y, c_z, $c_t - \frac{1}{Pe_g}\nabla^2 c$, and c. Results are shown for five different mice (M1–M5). Notice the large fluctuations and substantial variability across times and mice, spanning several orders of magnitude. (B) Time evolution of the scaled gradients after filtering and applying TD-RBA scaling. The maximum values are of order one, and means are consistent across all mice. This rescaling ensures uniform contribution of the residuals across times, improving the stability of the velocity inference process.

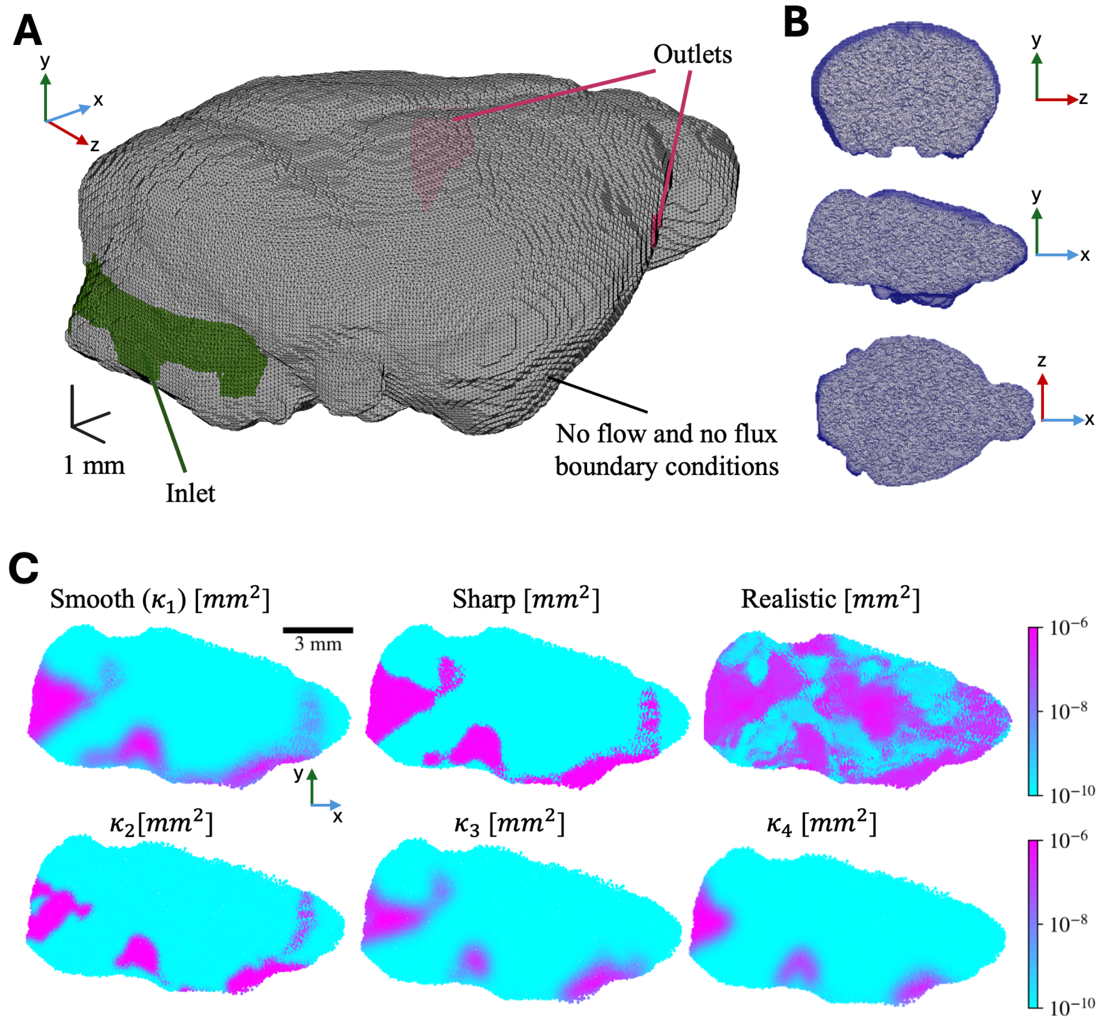

Figure S3: **Tetrahedral mesh and permeability maps for producing synthetic data.** **(A)** Finite element mesh used for simulations, consisting of approximately 2 million tetrahedral elements. The inlet, outlets, and no-flow boundary conditions are indicated. **(B)** Cross-sectional views of the mesh. **(C)** Permeability maps used in simulations: "Smooth" with gradual transitions, "Sharp" with abrupt interfaces, and "realistic" with a complex map that combines sharp and irregular transitions. Maps $\kappa_1 - \kappa_4$ were used as different initial guesses, allowing uncertainty quantification.

the meshes were sufficiently fine to resolve the computational domains and that the numerical results did not change substantially when the mesh size was decreased further. In the simulation results shown in this study, the total number of tetrahedral elements is approximately 2 million, and the mesh size ranges from 0.05 mm to 0.2 mm. The quintic finite element was used when solving the Darcy equation, and the quadratic finite element was used when solving the advection-diffusion equation.

The 3D domain boundaries were obtained from the segmentation of the brain region from Mouse 1 (see Figure S3(A)). In the simulations, we defined an inlet region close to and approximately the same size as the cisterna magna, and we placed two outlets on the domain surface at locations where tracer leaves the segmented brain region in the experimental data. When solving Darcy's law for the fluid velocity field, a constant pressure of 7.5 kPa was applied at the inlet, and zero pressure was applied at the outlets. When solving the advection-diffusion equation, a constant tracer concentration was applied at the inlet for the first 5 minutes, then set to 0 for the remainder of the simulation. All other domain boundaries are treated as no-flow (i.e., $\mathbf{n} \cdot \mathbf{u} = 0$, where $\mathbf{n}$ is the surface normal) when solving Darcy's law, and as no-flux (i.e., $\mathbf{n} \cdot \boldsymbol{\nabla} C = 0$) when solving the advection-diffusion equation (see Figure S3(A)).

To simulate DCE-MRI, we interpolated the concentration field onto a 0.1 mm square grid (the same resolution as the DCE-MRI data) at 1-minute increments for 90 minutes.

Concentration Reconstruction

We train our models using 50% of the available concentration data, which is used both to learn the concentration field and to extract aleatoric uncertainty via the denoising module composed of NN_c and NN_σ. Model performance is then evaluated on the remaining unseen data. As shown in Table S1, the validation error varies depending on the underlying permeability map.

For the "smooth" map, the model achieves a relative L^2 error of 0.2%; for the "sharp" map, performance decreases to 2%; and for the "realistic" map, the error is approximately 1%. Figure S4(A) shows the reference, prediction, and absolute error of the concentration at three representative times.

Additionally, Figure S4(B) shows that the error remains consistent across all times, with a slight degradation in performance at the beginning and end of the time domain. Figure S4(C) presents

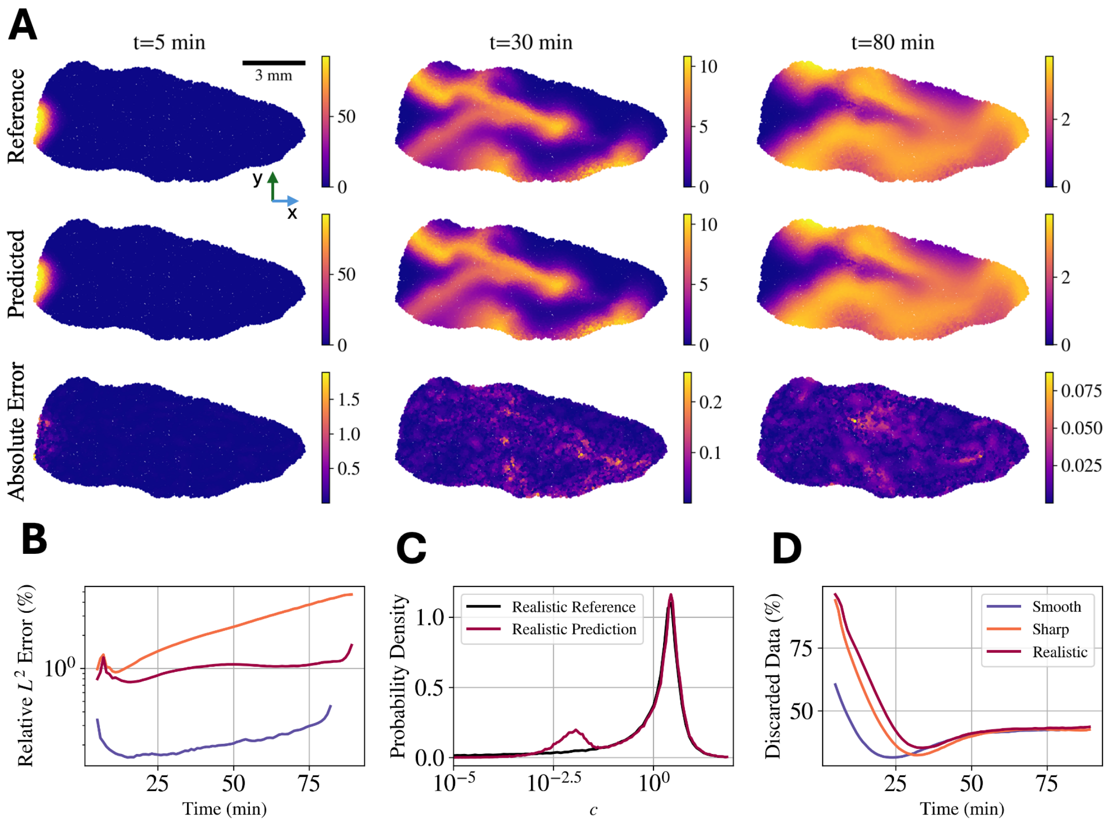

Figure S4: **Reconstruction of concentration using synthetic data.** (A) Tracer concentration in the mid-sagittal plane for the "realistic" synthetic case, at times $t = 5$, 30, and 80 minutes, as measured and as inferred. The reconstructed concentration shows spatially coherent transport. The absolute error, also shown, is greatest in regions of high concentration, particularly at early times, and decreases as the tracer spreads. (B) Relative L^2 error between inferred and measured concentrations varying over time for synthetic data with "smooth", "sharp", and "realistic" permeability. In the "realistic" case, the error usually remains below 2%, with an overall average near 1%. (C) Probability density functions of inferred and measured concentrations for the "realistic" case. The model accurately captures the full distribution across six orders of magnitude, though a mismatch appears in the low-concentration regime ($c < 10^{-3}$) due to weak signals. (D) Percentage of data discarded, based on thresholds tied to sensitivity, varying over time, for all synthetic cases. The discard rate is highest (up to 90%) for the "realistic" case and at early times; it decreases to about 40% as the tracer spreads.

the probability density function (PDF) of the concentration, illustrating that, even though the concentration in the "realistic" data set spans over six orders of magnitude, the model successfully captures the distribution.

Due to the wide dynamic range of the concentration field, we observe a mismatch between reconstructed and reference values in the low-concentration regime ($c < 10^{-4}$), as shown in Figure S4(C). Despite this, the concentration model is trained using all available observations, including those in the low-concentration regime. However, when inferring the velocity field, we introduce a post-processing step to discard regions with low concentration and weak gradients based on the denoised data. This filtering step is intended to prevent the propagation of low-information content into the PDE-constrained velocity learning stage. Specifically, we define a threshold on both the concentration and its gradient to identify regions that provide an insufficient signal for supervising the velocity field. These regions are explicitly excluded from the velocity inference process, as their inclusion could degrade performance by introducing weak or ambiguous supervision. The specific details of the filtering process are described in SM.

We observe that, most of the time, at least 40% of the domain falls below this threshold (see Figure S4(D)). Interestingly, the percentage of discarded data increases to as much as 90% during early time points, aligning with regions where the model has the greatest difficulty reconstructing the concentration field (see Figure S4(B)).

Inferring hidden 3D fields

3D velocity Inference Since the true permeability is unknown in real data sets, we evaluate the robustness of velocity inference in the synthetic validation by using mismatched permeability estimates. Specifically, for the "smooth" case, we use the "sharp" permeability map as the initial estimate. Conversely, for both the "sharp" and "realistic" cases, we use the "smooth" map as the initial guess.

Figure S5(A) shows the reference, predictions, and point-wise relative error for the three synthetic cases. In all scenarios, the velocity magnitude spans more than four orders of magnitude. Notably, in the "smooth" case, the model achieves less than 25% relative error in nearly 90% of the domain. As shown in Table S1, the relative L^2 error for the velocity magnitude $||\mathbf{u}||$ is 12.7%.

The "sharp" permeability map, by contrast, induces abrupt transitions in the velocity field and

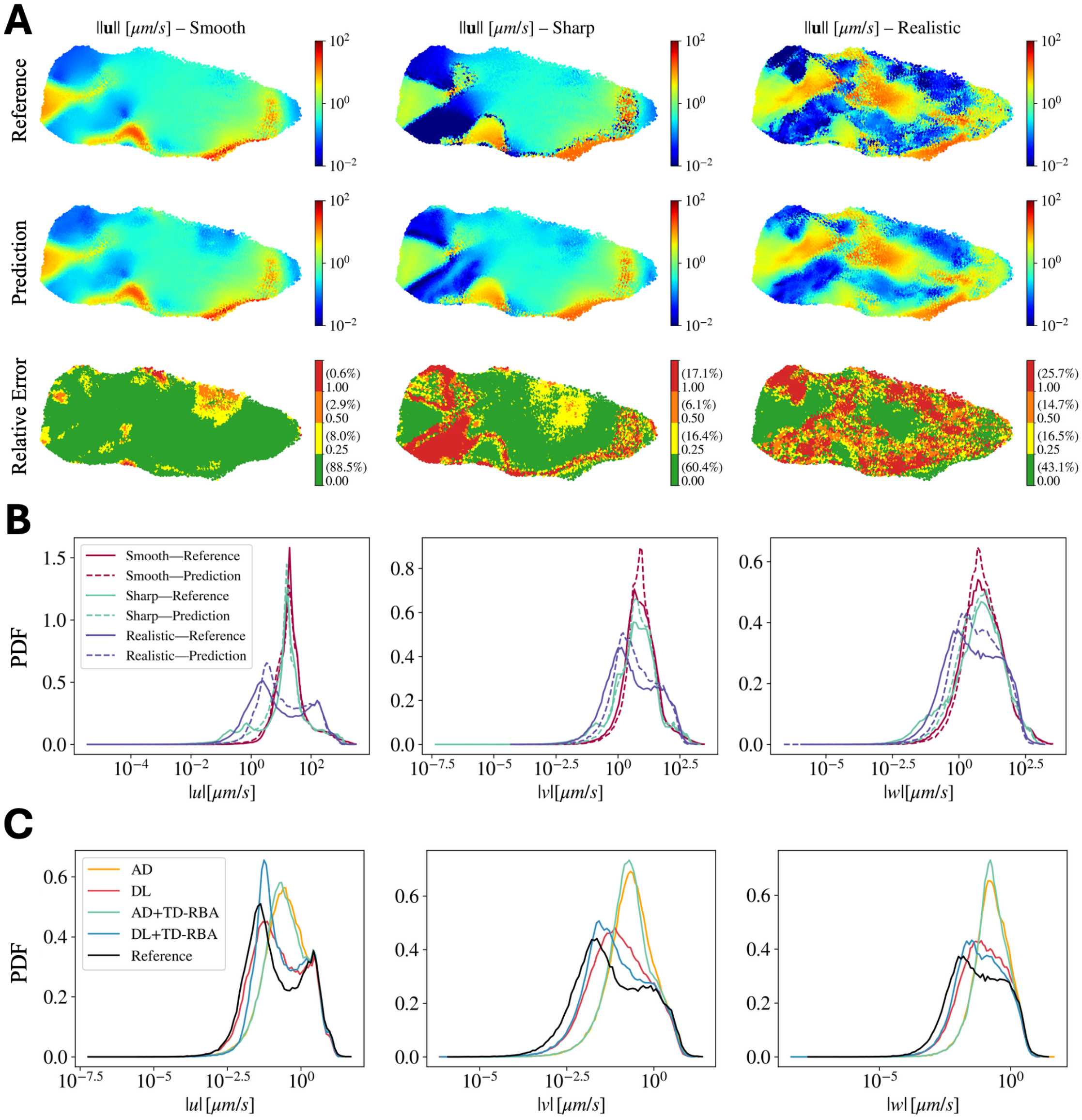

Figure S5: **Velocity inference using synthetic data.** (A) Reference, prediction, and relative error of the velocity magnitude $\|\mathbf{u}\|$ for three synthetic velocity fields: "smooth", "sharp", and "realistic". While accuracy is high for the "smooth" case, errors increase for the more complex fields, concentrating in low-velocity regions and at transition zones. (B) Probability density functions (PDFs) of velocity component magnitudes. The predicted distributions (dashed) accurately capture the reference (solid), including the bimodal structure induced by the "realistic" permeability, across five orders of magnitude. (C) PDFs from an ablation study on the "realistic" dataset. Only models enforcing Darcy's law (DL) recover the bimodal velocity distributions. Models using only the advection-diffusion equation (AD) predict a unimodal, averaged solution, likely due to convergence to poor local minima.

Table S1: **Reconstruction performance on synthetic data.** Relative L^2 errors, angular errors ($\bar{\theta}$) and Wasserstein distances (W_d) for different synthetic permeability maps. The table reports metrics for concentration (c), velocity (total magnitude $||\mathbf{u}||$ and components u, v, w), pressure (P), and permeability (κ).

	Relative L^2 Error (%) & Angular Error (°)								$W_d \cdot 10^2$		
Map	c	$\|\|\mathbf{u}\|\|$	u	v	w	P	κ	$\bar{\theta}$ (°)	$\log_{10}\|u\|$	$\log_{10}\|v\|$	$\log_{10}\|w\|$
Smooth	0.9	12.7	11.7	12.9	16.5	76.1	89.4	10.1	4.0	5.4	6.3
Sharp	2.3	26.2	25.0	33.2	30.4	77.1	63.8	16.4	14.1	10.9	13.7
Realistic	1.0	36.0	35.5	44.2	48.0	43.9	51.3	23.9	18.0	15.7	15.5

regions where the velocity becomes extremely small. Despite these challenges, the model still achieves less than 25% relative error in approximately 60% of the domain, with relative L^2 error for the velocity magnitude of 26.2%. As shown in Figure S5(A), the error is primarily concentrated in regions with very low velocity ($||\mathbf{u}|| \leq 10^{-2}$µm/s) and at transitions between high and low velocities.

This pattern persists in the "realistic" case, which contains a larger fraction of low-velocity regions. For this case, the relative L^2 errors for the velocity magnitude reach36%. However, as shown in Figure S5(A), the errors are primarily concentrated in low-velocity regions. Nevertheless, about 40% of the domain still exhibits relative errors less than 25%. This highlights an important limitation of our method: it struggles to reconstruct extremely low velocities. This behavior is expected, as very slow velocities result in minimal tracer transport (see Figure S4(C)), which limits the information available for accurate reconstruction.

Finally, Figure S5(B) shows that, in the "sharp" and "realistic" cases, the velocity components span a range from 10^{-2} µm/s to 10^{2} µm/s, with most values concentrated between $10^{-2.5}$ µm/s and $10^{-1.5}$ µm/s. Despite this challenging distribution, the predicted velocity histograms closely match the reference distributions, demonstrating the model's ability to recover the full range of values. Moreover, in the "realistic" case, the complexity of the permeability map induces bimodal distributions in the velocity components, which are also successfully captured by the model.

Permeability and Pressure Estimates As shown in Figure S6, models successfully capture the overall spatial structure of both pressure and permeability across all three synthetic cases.

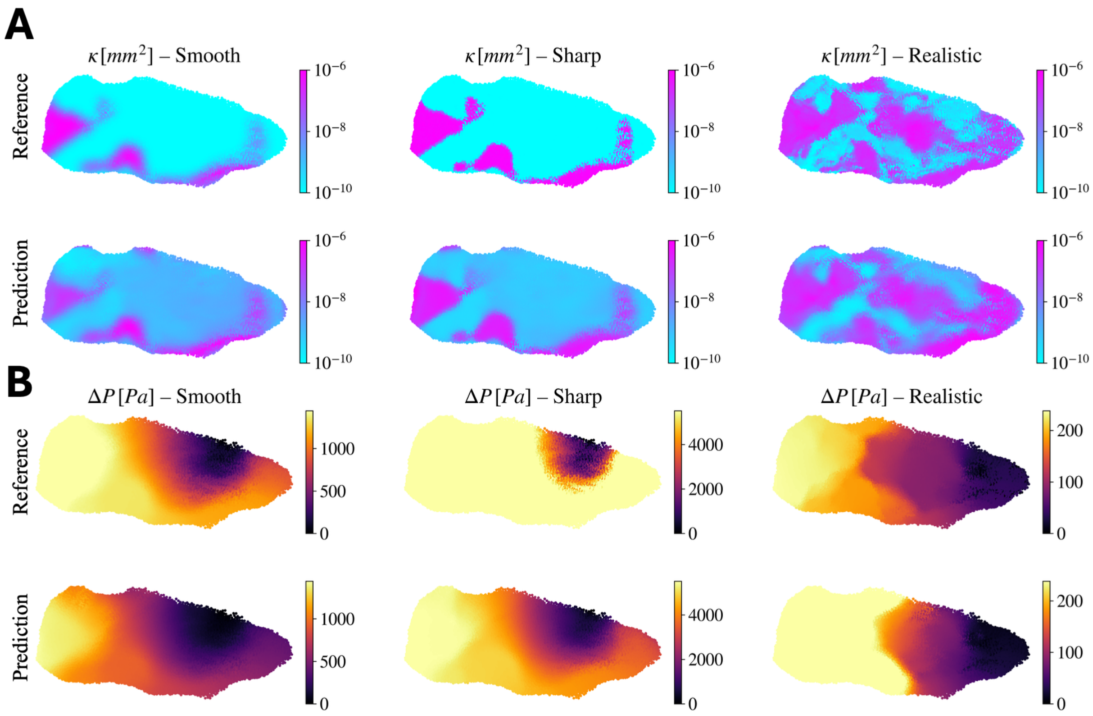

Figure S6: **Pressure and permeability estimation using synthetic data.** (A) Reference and predicted permeability fields for synthetic data with "smooth", "sharp", and "realistic" permeabilities. Models successfully capture the spatial structure and magnitude of the permeability in all cases. (B) Reference and predicted pressure variation fields ($\Delta P = P - P_{min}$) for the same datasets. The model reproduces the spatial distribution of the pressure field.

The predicted fields exhibit strong agreement with the reference fields, preserving key structural features and regional contrasts. However, while the spatial trends are well recovered, the absolute magnitudes of the predicted fields deviate from the ground truth. This discrepancy is reflected in the relative L^2 errors reported in Table S1, where pressure errors range from 76.1% to 48.6%, and permeability errors from 89.4% to 51.3% across the "smooth", "sharp", and "realistic" cases.

These high errors in magnitude stem from the inherent ambiguity in estimating pressure and permeability from velocity-constrained data. According to Darcy's law, $\mathbf{u} = -K\nabla P$, the same velocity field can result from many different combinations of K and ∇P. Since the concentration data only constrains the velocity field through the advection–diffusion equation, it cannot uniquely determine either K or P. The improved accuracy observed in the "realistic" case likely arises from the added flow complexity, which imposes stronger implicit constraints and reduces the space of admissible solutions. As discussed in SM, some permeability initial guesses induce considerably lower errors; nonetheless, the inverse problem remains ill-posed, and the inferred fields should be interpreted as plausible and physically consistent, but not unique.

Real Data Additional Results

Front tracking for initial velocity estimate

In order to provide an initial estimate of the velocity field, we track how "fronts", or regions of constant concentration, move over time. We track fronts corresponding to SER of 25 to 75. For each SER level, we binarize the three-dimensional data at each time point, using the SER level as the threshold. We smooth the binarized image using a three-dimensional box filter, then find the edge of the binarized region, or the front. We estimate the velocity by tracking the front location over time. For each point on the front, we calculate the surface normal and find the distance along the surface normal to the front location at the next time point. We average the velocity estimates from all of the front levels. The front tracking algorithm is available on Zenodo at `https://zenodo.org/records/15345392`.

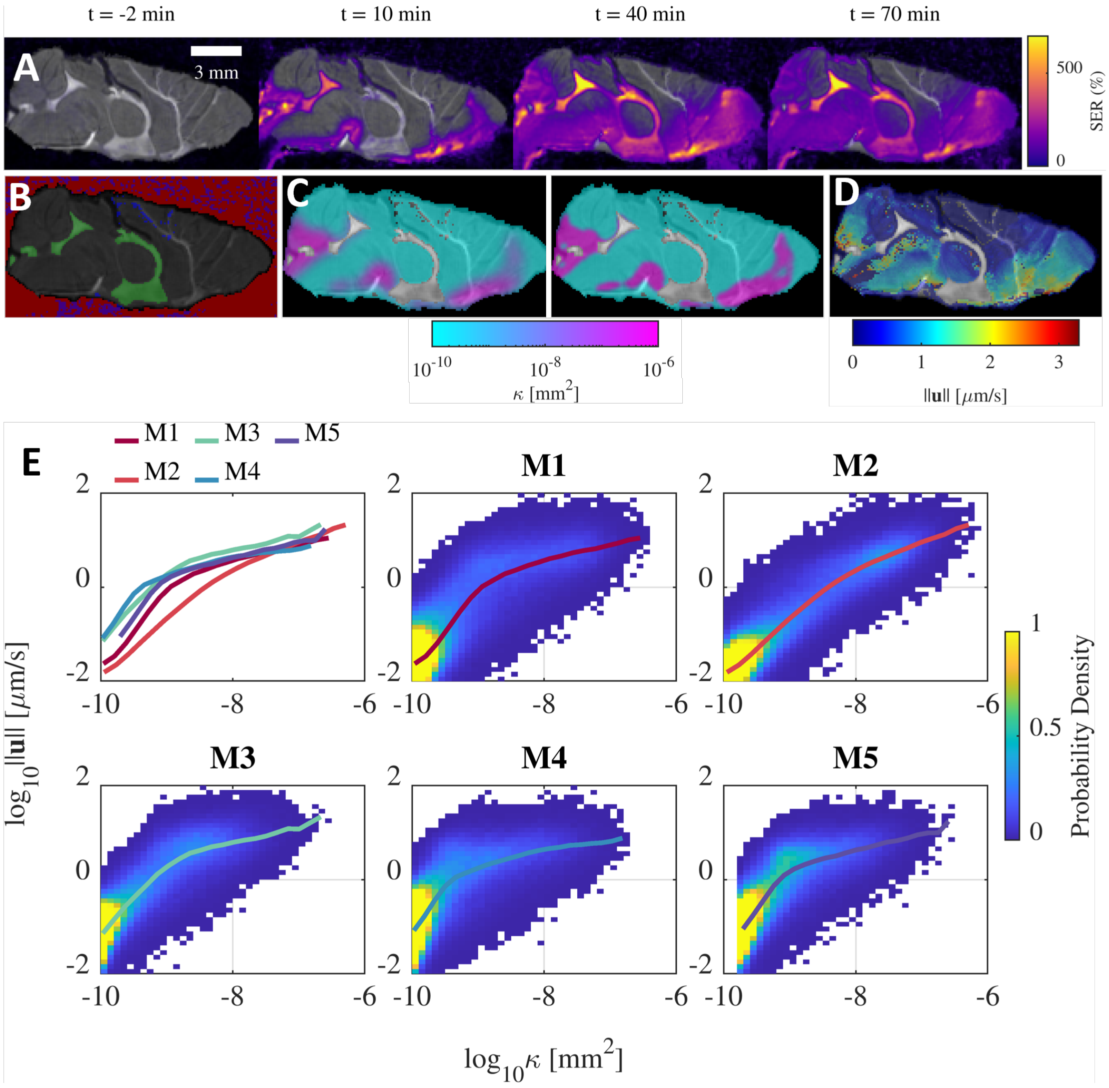

Figure S7: **DCE-MRI data and preprocessing.** (A) Signal enhancement ratio (SER) over time for Mouse 1 after gadobutrol injection. (B) Excluded regions for analysis. (C) Initial permeability estimates ("smooth" and "sharp") derived from early tracer concentration. (D) Initial velocity estimate from front tracking. (E) **Velocity magnitude and permeability are closely coupled.** The bivariate probability density (heat map) and conditional average (solid line) for each mouse confirm that high velocities occur in regions with high inferred permeability.

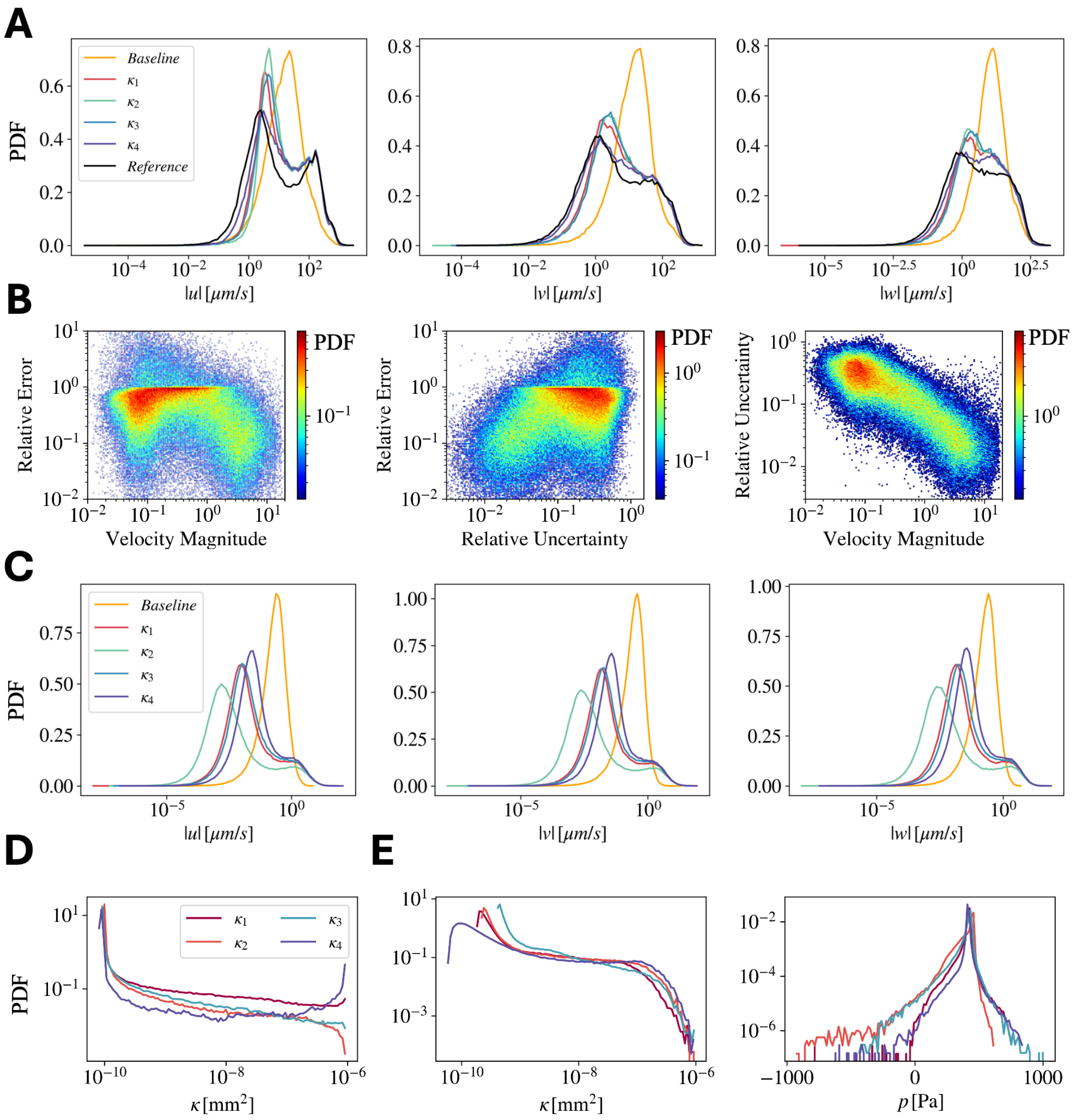

Figure S8: **Distributions and uncertainty quantification.** (A) Probability density functions (PDFs) of velocity component magnitudes for the "realistic" synthetic data. Models enforcing Darcy's law successfully capture the bimodal structure of the reference data, while the baseline model (which only enforces advection-diffusion and continuity) fails. (B) Joint distributions for the synthetic data show that relative error is highest in slow regions and that predicted uncertainty strongly correlates with error. (C) PDFs of velocity component magnitudes for Mouse 1. (D) PDFs of the initial permeability guesses. (E) PDFs of the inferred permeability and pressure for Mouse 1, using different initial permeability guesses.

Table S2: **Median velocity in deep brain regions.** Median velocity magnitude ||**u**|| [µm/s] for selected deep brain regions and the sagittal sinus. Data is shown for five mice (M1-M5) along with the median and standard deviation across all mice.

	Hippocampus	Caudate	Thalamus	Midbrain	Sagittal Sinus
M1	0.043	0.029	0.028	0.042	0.097
M2	0.025	0.030	0.024	0.035	0.515
M3	0.104	0.066	0.074	0.115	1.050
M4	0.154	0.097	0.060	0.103	1.039
M5	0.205	0.123	0.110	0.128	0.578
Median	0.106	0.069	0.059	0.085	0.656
Deviation	0.067	0.037	0.032	0.039	0.358

Permeability and Pressure Estimation

To enable consistent visualization across mice, we apply a normalization step that enforces a common maximum permeability of 10^{-6} mm^2 and displaces each pressure field so that all models have the same mean. This transformation is justified because Darcy's law, $\boldsymbol{u} = -K\boldsymbol{\nabla}P$, is invariant under the rescaling and translation $-\frac{1}{s}K \cdot s\boldsymbol{\nabla}(P - \text{const}) = -K\boldsymbol{\nabla}P$. While this normalization allows for meaningful visual comparison, it does not resolve the fundamental non-uniqueness in recovering K and P from velocity alone. Consequently, the inferred fields should be interpreted as plausible and physically consistent estimates, not as unique reconstructions.

Table S3: **Median velocity in perivascular and subarachnoid spaces.** Median velocity magnitude $||\mathbf{u}||$ [µm/s] for selected perivascular spaces (PVS) and the subarachnoid space (SAS) of the olfactory bulb. Acronyms: CoW, Circle of Willis; MCA, middle cerebral artery; ACA, anterior cerebral artery. Data is shown for five mice (M1-M5) along with the median and standard deviation across all mice.

	PVS-CoW	PVS-MCA	PVS-ACA	PVS-basilar	SAS-Olfactory
M1	2.371	0.343	2.444	3.032	0.802
M2	2.189	0.980	2.780	2.971	1.095
M3	3.030	3.251	4.951	3.691	4.370
M4	1.377	1.203	1.869	2.090	1.180
M5	2.354	2.380	3.521	2.520	3.758
Median	2.264	1.632	3.113	2.861	2.241
Deviation	0.529	1.044	1.063	0.537	1.506

Table S4: **Median permeability in deep brain regions.** Median permeability κ ($\times 10^{-10}$ [mm^2]) for selected deep brain regions and the sagittal sinus. Data is shown for five mice (M1-M5) along with the median and standard deviation across all mice.

	Hippocampus	Caudate	Thalamus	Midbrain	Sagittal Sinus
M1	3.86	3.73	3.95	4.35	5.37
M2	2.73	2.45	2.54	3.38	17.8
M3	4.35	3.93	3.96	4.25	16.4
M4	6.46	6.11	5.96	6.78	10.8
M5	7.81	6.82	6.50	6.87	13.0
Median	4.35	3.93	3.96	4.35	13.0
Deviation	1.84	1.62	1.45	1.43	4.42

Table S5: **Median permeability in perivascular and subarachnoid spaces.** Median permeability κ ($\times 10^{-10}$ [mm^2]) for selected perivascular spaces (PVS) and the subarachnoid space (SAS) of the olfactory bulb. Acronyms: CoW, Circle of Willis; MCA, middle cerebral artery; ACA, anterior cerebral artery. Data is shown for five mice (M1-M5) along with the median and standard deviation across all mice.

	PVS-CoW	PVS-MCA	PVS-ACA	PVS-basilar	SAS-Olfactory
M1	110	11.5	155	31.9	41.8
M2	148	45.2	281	78.1	79.9
M3	49.3	44.3	135	26.4	123
M4	32.4	27.6	127	35.1	22.6
M5	55.4	69.0	153	23.5	169
Median	55.4	44.3	153	31.9	79.9
Deviation	43.2	19.2	56.3	20.0	53.2

Sensitivity Analysis

To evaluate the robustness of the MR-AIV framework, we performed a targeted sensitivity analysis. This section details the Ablation Study, which isolates the specific performance contributions of the physics-constrained architecture and the Time-Dependent Residual-Based Attention (TD-RBA) weighting scheme, and an Uncertainty Quantification (UQ), which assesses the model's sensitivity to the data-driven permeability initialization.

Ablation Study

The proposed framework incorporates several key enhancements, which can be decomposed into the following components: (1) a specialized architecture that strictly enforces Darcy's law (DL), and (2) a time-dependent residual-based attention (TS-RBA) weighting scheme. To evaluate the contribution of each component, we performed an ablation study comparing the DL-based architecture against a baseline architecture (AD), which consists of a single multi-layer perceptron (MLP) that predicts the velocity components (u, v, w) without explicitly enforcing Darcy's law. All models were trained using the same sequential strategy and denoising module to ensure a fair comparison.

Table S6: **Ablation study of model architecture and training strategies.** The baseline architecture (AD) uses a direct MLP to predict velocity without enforcing Darcy's law, while the DL architecture encodes this constraint explicitly. All models share the same denoising module and sequential training for fair comparison. Relative L^2 errors measure average accuracy, and Wasserstein distances (W_d) assess the quality of the reconstructed velocity distributions. While AD achieves similar relative L^2 errors, it fails to capture the correct distributions. DL reduces W_d substantially, and TD-RBA further improves the recovery of velocity, pressure, and permeability.

	Relative L^2 Error (%)						$W_d \cdot 10^{-2}$		
Architecture	c	u	v	w	P	κ	$\log_{10}\|u\|$	$\log_{10}\|v\|$	$\log_{10}\|w\|$
AD	0.9	35.0	45.8	71.1	-	-	34.5	54.4	52.8
DL	0.9	43.2	56.5	60.5	80.0	85.8	9.2	23.1	21.2
AD+TD-RBA	1.0	36.9	45.9	54.1	-	-	33.2	51.5	51.4
DL+Seq+TD-RBA	1.0	35.5	44.2	48.0	43.9	51.3	18.0	15.7	15.5

Table S6 summarizes the results of the ablation study across different model configurations. Notice that the DL-based formulation is crucial to reduce the errors, especially in the w-component, which is harder to recover due to its considerably smaller magnitude (See Figure S5). Moreover, the Wasserstein distance (W_d) reveals a critical limitation of the AD architecture: despite similar average errors, it fails to capture the correct velocity distributions, as reflected by substantially higher W_d values across all components. This limitation is further illustrated in Figure S5(A), which shows the distributions of the velocity component magnitudes $|u|$, $|v|$, and $|w|$. Only the DL-based models recover the characteristic bimodal structure of the velocity distribution, while the AD models tend to collapse toward intermediate values between the two peaks, effectively predicting an averaged solution, which may reflect convergence to a poor local minimum.

The DL architecture consistently improves the distributional accuracy, substantially reducing the Wasserstein distance, especially for the dominant velocity component. Furthermore, the inclusion of the TD-RBA weighting scheme provides additional gains, particularly when combined with sequential training. Notably, TD-RBA not only improves velocity reconstruction but also facilitates the recovery of pressure and permeability fields, which are otherwise unstable without these enhancements.

Uncertainty Quantification

Psaros et al. (*83*) identified three main sources of uncertainty in PIML: aleatoric uncertainty, which originates from noise or sparsity in the data; epistemic uncertainty, which stems from limitations in the model architecture, initialization, or assumptions; and model uncertainty, which arises from assumptions in the governing equations. In our approach, aleatoric uncertainty in the concentration field is estimated directly from the experimental data using a denoising network trained with a negative log-likelihood loss, providing uncertainty estimates for concentration measurements (Figure 2(A)).

Epistemic uncertainty, which reflects the sensitivity of model predictions to architectural choices and parameter initialization, is traditionally assessed by training multiple models with different random initializations (*84*). In our framework, however, due to the use of a pre-training stage, the initial permeability guess effectively defines the parameter initialization for the velocity inference. To quantify this uncertainty, we adopt an ensemble-of-models (EoM) approach (*10, 47, 85*), where multiple models are trained independently, each initialized with a different permeability guess. Following (*47*), we approximate the ensemble prediction as a Gaussian distribution, with the mean and variance given by the empirical mean and variance of the individual model outputs, as defined in equations S21 and S22, respectively.

$$\bar{B}(\boldsymbol{x}) = \frac{1}{M}\sum_{m=1}^{M} B_m(\boldsymbol{x};\theta_{1,m},\theta_{2,m}) \tag{S21}$$

$$\sigma_B^2(\boldsymbol{x}) = \frac{1}{M}\sum_{m=1}^{M} \left(B_m(\boldsymbol{x};\theta_{1,m},\theta_{2,m}) - \bar{B}(\boldsymbol{x})\right)^2 \tag{S22}$$

where $B \in \{u, v, w, P, K\}$ denotes the physical quantities of interest: the velocity components (u, v, w), pressure (P), and permeability (K).

We apply this method to both synthetic ("realistic" map) and real data sets (M1) using four different initial permeability maps (see Figure S3). One of our key assumptions is that we can derive a reasonable initial guess of the permeability map from the early tracer distribution. To evaluate the sensitivity of the inferred results on the initial guess, we compare results using κ_3 and κ_4 permeability fields derived from Mice 3 and 4, respectively, which differ substantially from the standard guess κ_1 (from Mouse 1). Additionally, to isolate the influence of structural sharpness,

Table S7: **Permeability-based sensitivity analysis.** The model shows strong and consistent performance in the velocity components, with relative errors in the velocity magnitude $||\mathbf{u}||$ varying by less than 2% across all cases. For pressure and permeability, the model can achieve low errors, but the uncertainty is substantially higher due to the ill-posed nature of the inverse problem under Darcy's law. As a result, some initializations lead to accurate predictions while others do not. Nonetheless, the model consistently captures the overall distribution of the permeability and substantially reduces the Wasserstein distance (W_d) between the log-scaled initial guess κ and the final prediction κ.

Model	W_d ($\times 10^{-2}$)		Relative L^2 Error (%)			
	(Initial) $\log_{10}\|\kappa\|$	$\log_{10}\|\kappa\|$	c	$\|\|\mathbf{u}\|\|$	P	κ
κ_1	147.3	27.8	1.2	36.0	41.9	59.1
κ_2	162.3	51.3	1.0	35.7	86.9	85.0
κ_3	175.2	39.3	1.0	35.9	129.7	63.1
κ_4	170.8	28.4	1.0	34.2	35.6	65.7
Average	163.9	36.7	1.1	35.4	73.5	68.2
Std.	10.7	9.6	0.1	0.7	38.0	9.9

we include the map κ_2, which is derived from the Mouse 1 data and contains abrupt transitions designed to challenge the learning process. This ensemble analysis enables a systematic evaluation of the model's sensitivity to permeability initialization and the robustness of the inferred fields.

Finally, to provide a coarse estimate of the modeling uncertainty, we include results from a baseline model that infers the velocity field using only the advection-diffusion equation. This model is not physically well-posed, as the combination of advection-diffusion and mass conservation involves four coupled unknowns, but it serves to illustrate the limitations of relying solely on tracer transport without incorporating additional physical constraints.

Velocity Inference

Synthetic Data Figure S8(A) shows the distribution of the predicted velocity components for models trained using Darcy's law under different initial permeability guesses κ_1–κ_4. The model

captures a wide dynamic range, particularly for the w component, which spans from 10^{-6} μm/s to 10^{3} μm/s. Additionally, the predictions consistently recover a bimodal distribution across all components, indicating robust structural consistency despite variations in permeability initialization. For comparison, we also show the velocity distribution from a baseline model that does not encode Darcy's law in this architecture and predicts the velocity components directly. In this case, the advection-diffusion equation and mass conservation are insufficient to constrain the four unknowns c, u, v, and w, leading the model to collapse to a unimodal, average-like solution.

Table S7 reports the relative L^2 errors for each velocity component across permeability initializations. The model exhibits consistent performance, with an average relative L^2 error of 35.4%. The variation across models is small, with standard deviations below 2% for all components.

Finally, to visualize the uncertainty distribution from different permeability initializations, Figure 8(A) shows the mean velocity magnitude $||\bar{\mathbf{u}}|| = \sqrt{\bar{u}^2 + \bar{v}^2 + \bar{w}^2}$ computed from the mean velocity components $\bar{u}, \bar{v}, \bar{w}$ calculated from the ensemble average; see Equation S21. Similarly, the figure also shows the relative uncertainty, computed as $\sigma_{||\mathbf{u}||}/||\bar{\mathbf{u}}||$, where $\sigma_{||\mathbf{u}||}$ denotes the standard deviation of the velocity magnitude across the ensemble. The bottom row presents the point-wise relative error with respect to the ground-truth velocity magnitude $||\hat{\mathbf{u}}||$, computed as $|||\bar{\mathbf{u}}|| - ||\hat{\mathbf{u}}|||/||\bar{\mathbf{u}}||$. We observe that regions of high uncertainty often coincide with regions of high error, particularly near sharp permeability interfaces. Additionally, both error and uncertainty are elevated in low-velocity regions, where reconstruction is more sensitive to the initial guess. To quantify this observation, Figure S8(B) shows the joint distributions of relative error vs. average velocity magnitude, relative error vs. relative uncertainty, and relative uncertainty vs. velocity magnitude. Notably, relative errors are concentrated in low-velocity regions, and the predicted uncertainty strongly correlates with true error. Relative errors can fall below 10% in regions of higher velocity, and overall, the relative error remains below 50% across most of the domain, indicating stable velocity reconstruction despite variations in permeability initialization.

Real Data Figure 8(B) shows the distribution of the predicted velocity components for Mouse 1 using different permeability initial guesses. As in the synthetic case, the predicted velocities exhibit a bimodal structure, with peaks at low and high velocities. While the high-velocity peak is consistent across models, there is some variability in the location and height of the low-velocity peak, typically

centred around $10^{-2}\,\mu$m/s. In contrast, the baseline model, which does not enforce Darcy's law, predicts a unimodal distribution, again indicating a collapse toward an average behaviour due to the lack of physical constraints. Overall, the ensemble captures a wide range of velocities spanning over four orders of magnitude, demonstrating the model's flexibility and ability to resolve heterogeneous flow regimes even in the absence of ground-truth velocity data.

Figure 8(B) maps the mean velocity magnitude and its associated relative uncertainty for Mouse 1. Although we cannot compute point-wise errors in the real data due to the lack of a reference solution, the uncertainty map provides insight into prediction reliability. From the synthetic case, we observed that high uncertainty typically aligns with regions of low velocity, which are more sensitive to permeability initialization. A similar pattern emerges here: uncertainty is elevated in low-flow regions but remains below 100% across most of the domain. In contrast, uncertainty is low in regions with high velocity, suggesting that the model's predictions are more reliable in those areas, even under varying initialization. Even though the relative uncertainty is large (uncertainty between 50 and 100% for over half of the brain), we consider this level of uncertainty acceptable, given that velocities in the deep brain were previously so uncertain.

Permeability and Pressure estimates

Synthetic Data Table S7 summarizes the variability in pressure and permeability reconstructions across different initializations. In contrast to the velocity components, which exhibit stable performance regardless of the starting point, both pressure and permeability are notably more sensitive to initialization. This behaviour reflects the ill-posed nature of the inverse problem under Darcy's law, where multiple fields can satisfy the observed velocity data. As a result, some initializations yield relatively accurate estimates (e.g., κ_3 with 35.6% error in pressure and 65.7% in permeability), while others lead to substantially higher errors. The elevated standard deviations in pressure (43.4%) and permeability errors (14.0%) underscore this inconsistency.

Despite this variability, all inferred permeability fields consistently reduce the mismatch between the initial guess (in log scale) and the final prediction, as measured by the Wasserstein distance. This distance provides a meaningful assessment of structural similarity between the predicted and true fields beyond point-wise errors. Spatial maps of the average predictions, relative uncertainties,

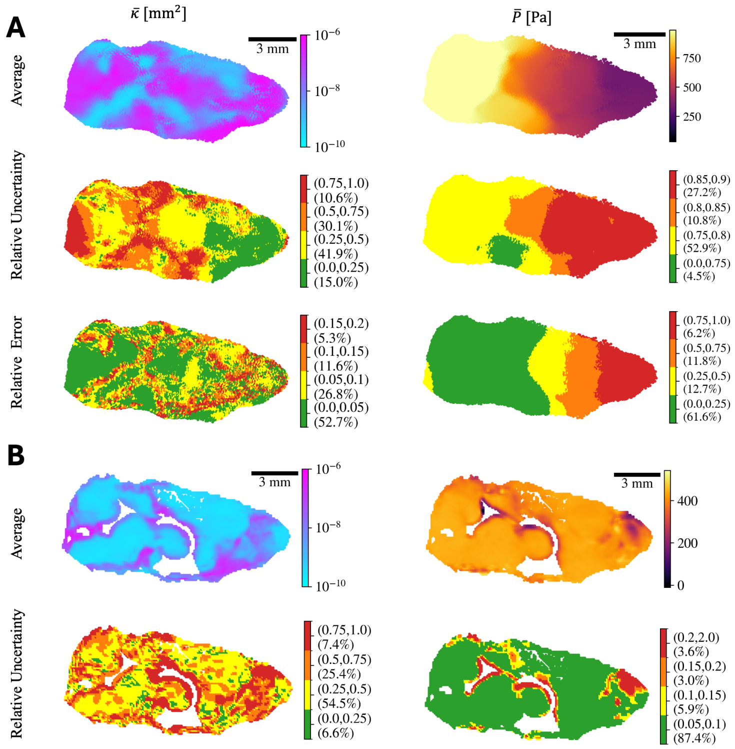

Figure S9: **Uncertainty quantification of the inferred permeability and pressure fields. (A)** (Top row) Average permeability $\bar{\kappa}$ and pressure $\bar{P}$ computed from four models with different initial permeability guesses, for the "realistic" synthetic data. (Middle row) Relative uncertainty across the four models. Colors are marked with the percentage of total brain volume to which each applies. (Bottom row) Relative error. Uncertainty is high around transition regions, which typically correspond to interfaces between low and high permeability zones. The permeability relative error reflects similar spatial trends and remains below 20% and 25% for permeability and pressure in most of the domain. **(B)** (Top row) Average permeability and pressure computed from four models with different initial permeability guesses, for Mouse 1. (Bottom row) Permeability relative uncertainty is highest near structural transitions and regions of elevated spatial variation, as with synthetic data. Pressure relative uncertainty is high near sharp gradients, as with the synthetic data, but is low in most of the domain.

and errors are presented in Figure S9 for permeability and pressure.

Real Data The in-vivo results exhibit similar qualitative behaviour. As shown in Figure S9, uncertainty is primarily concentrated in regions with strong anatomical transitions and steep gradients. Notably, Figure S8 (D) shows that the predicted pressure and permeability remain surprisingly consistent across different initial guesses. This resemblance should not be misinterpreted as evidence of uniqueness. Rather, it may reflect the inherent complexity and spatial richness of the underlying field, which can effectively constrain the space of admissible solutions. Nevertheless, due to the ill-posed nature of the inverse problem, these reconstructions should be interpreted as plausible and physically consistent estimates, not as definitive ground-truth representations.

Implementation Details

Training

For our experiments, development, training, validation, and visualization, we used JAX 0.3.2.9 as a machine learning framework on a single Nvidia A100-80GB GPU.

All models were trained using four AdamW (*77*) optimizers (i.e., one per network) with a weight decay of 10^{-5} for regularization. The initial and final learning rates are 2×10^{-3} and 5×10^{-5}, respectively, with a decay rate of 0.99.

For the RBA-R hyperparameters, we follow (*36*) and use $\gamma = 0.999$, $\eta = 0.1$, $\nu = 2.0$, and $c = 0.5$ (see equation S17). All models were trained using a batch size of $bs = 10000$.

Sequential Training In all cases, the sequential strategy was imposed by varying the global weights m_α during the initialization and training stages as described in Table 2, respectively.

Hyperparameter Selection

The multi-stage training pipeline employs a modular architecture to manage the problem's complexity. This structure allows for training components in stages. For example, the denoising network is trained first, and its parameters are then *frozen*. The output from this network is subsequently used as the concentration signal for the physics-based networks. This sequential approach decom-

poses the optimization problem into a series of sub-problems. The following sections detail the selection logic for the key hyperparameters, which were chosen based on the problem's physical and numerical characteristics.

Automatic Local Balancing (TD-RBA) The weighting of individual spatiotemporal points is addressed by the Time-dependent Residual-Based Attention with Resampling (TD-RBA) mechanism. The hyperparameters for this RBA-R component were adopted directly from cited literature (*36, 49, 79*) based on their established properties. For instance, the RBA (*49*) parameters ($\eta = 0.1$, $\gamma = 0.999$) define a maximum attention weight of $\lambda_{\max} \approx \eta/(1-\gamma) = 10$. As the loss scales as $\mathcal{L} \sim (\lambda r)^2$, this allows the loss contribution of high-residual points to be amplified by up to 100×. The memory term $\gamma = 0.999$ creates an averaging window of approximately 1000 iterations, which was shown in previous studies (*36*) to be stable for batch sizes (e.g., $10,000$) similar to those used here.

Global Balancing (Loss Weights) The global loss weights (m_α) detailed in Tables S9 and S10 were set by considering the expected physical magnitudes of the equation residuals. This balancing is intended to allow all physical constraints to contribute to the loss function. For example, the permeability field K exhibits sharp, 4-order-of-magnitude transitions at interfaces. Consequently, the Conservation of Mass residual (r_{CM}), which involves the derivative ∇K, becomes numerically large at these transitions, resulting in residuals roughly 10 to 10^2 times larger than advection diffusion equation loss (See Figure S1). The weights were therefore chosen to compensate for this disparity, bringing both residual terms to a similar order of magnitude.

Network Architecture Design Network architectures were also based on established design principles. To enhance network expressivity (*71*), a fifth-degree Chebyshev polynomial feature expansion was used. This was selected as Chebyshev-based networks (*51*) tend to be more robust to noise, which is relevant for experimental data. Higher polynomial orders were avoided due to potential instabilities (*51*). We also employ adaptive residual connections to prevent vanishing gradients during training (*73*). The network depth (five adaptive residual layers, equivalent to a 9-layer standard MLP) is comparable to prior AIV studies (*9*). Finally, network widths were set

proportionally to their input dimensions: NN_c (4 inputs: t, x, y, z) uses 200 neurons, while NN_K and NN_P (3 inputs: x, y, z) use 150 neurons, maintaining a 4:3 ratio.

Training Duration (Iterations) Finally, the number of iterations for each training stage was not pre-set but was determined by monitoring convergence. In accordance with standard machine learning practice, each stage is trained until its respective loss function plateaus, as visualized in Figure S1. Training is continued until the loss plateaus to ensure convergence for the sub-problem. The specified iteration counts thus represent the point of convergence for each sub-problem.

Movies

Caption for Movie S1 Velocity magnitude overlaid on the 3D structural image The MR-AIV-inferred velocity magnitude (semitransparent) is overlaid on the three-dimensional greyscale structural image for Mouse 1. Regions of high velocity magnitude align with known anatomical structures where fast flow is expected.

Caption for Movie S2 Measured and reconstructed concentration in a mid-sagittal plane overlaid on the structural image for Mouse 1 Concentration evolution over time shows good agreement between the measured and reconstructed concentration.

Caption for Movie S3 Measured and reconstructed concentration in a mid-sagittal plane overlaid on the structural image from Mouse 3 Concentration evolution over time shows good agreement between the measured and reconstructed concentration.

Caption for Movie S4 Pe overlaid on the 3D structural image The local Péclet number (semi-transparent) is overlaid on the three-dimensional greyscale structural image for Mouse 1, with the transparency emphasizing advection-dominated regions (i.e. $Pe \gg 1$). Advection dominates in many regions where the velocity is large, e.g., near the olfactory bulb, cisterna magna, and Circle of Willis.

REFERENCES

1. L. Xie, H. Kang, Q. Xu, M. J. Chen, Y. Liao, M. Thiyagarajan, J. O'Donnell, D. J. Christensen, C. Nicholson, J. J. Iliff, T. Takano, R. Deane, M. Nedergaard, Sleep drives metabolite clearance from the adult brain. *Science* **342**, 373–377 (2013).

2. M. K. Rasmussen, H. Mestre, M. Nedergaard, The glymphatic pathway in neurological disorders. *Lancet Neurol.* **17**, 1016–1024 (2018).

3. B. A. Plog, M. Nedergaard, The glymphatic system in central nervous system health and disease: Past, present, and future. *Annu. Rev. Pathol.* **13**, 379–394 (2018).

4. H. Mestre, T. Du, A. M. Sweeney, G. Liu, A. J. Samson, W. Peng, K. N. Mortensen, F. F. Stæger, P. A. R. Bork, L. Bashford, E. R. Toro, J. Tithof, D. H. Kelley, J. H. Thomas, P. G. Hjorth, E. A. Martens, R. I. Mehta, O. Solis, P. Blinder, D. Kleinfeld, H. Hirase, Y. Mori, M. Nedergaard, Cerebrospinal fluid influx drives acute ischemic tissue swelling. *Science* **367**, eaax7171 (2020).

5. D. H. Kelley, J. H. Thomas, Cerebrospinal fluid flow. *Annu. Rev. Fluid Mech.* **55**, 237–264 (2023).

6. H. Mestre, J. Tithof, T. Du, W. Song, W. Peng, A. M. Sweeney, G. Olveda, J. H. Thomas, M. Nedergaard, D. H. Kelley, Flow of cerebrospinal fluid is driven by arterial pulsations and is reduced in hypertension. *Nat. Commun.* **9**, 4878 (2018).

7. F. M. Rivas, J. Liu, B. C. Martell, D. Ting, H. Mestre, M. Nedergaard, J. Tithof, J. H. Thomas, D. H. Kelley, Surface periarterial spaces of the mouse brain are open, not porous. *J. R. Soc. Interface* **17**, 20200593 (2020).

8. A. Raghunandan, A. Ladrón-de-Guevara, J. Tithof, H. Mestre, D. Ting, M. Nedergaard, J. H. Thomas, D. H. Kelley, Bulk flow of cerebrospinal fluid observed in periarterial spaces is not an artifact of injection. *eLife* **10**, e65958 (2021).

9. K. A. S. Boster, S. Cai, A. Ladrón-de-Guevara, J. Sun, X. Zheng, T. Du, J. H. Thomas, M. Nedergaard, G. E. Karniadakis, D. H. Kelley, Artificial intelligence velocimetry reveals in vivo

flow rates, pressure gradients, and shear stresses in murine perivascular flows. *Proc. Natl. Acad. Sci. U.S.A.* **120**, e2217744120 (2023).

10. J. D. Toscano, W. Chenxi, A. Ladrón-de-Guevara, D. Ting, M. Nedergaard, D. H. Kelley, G. E. Karniadakis, K. A. S. Boster, Inferring in vivo murine cerebrospinal fluid flow using artificial intelligence velocimetry with moving boundaries and uncertainty quantification. *Interface Focus* **14**, 20240030 (2024).

11. B. A. Plog, H. Mestre, G. E. Olveda, A. M. Sweeney, H. M. Kenney, A. Cove, K. Y. Dholakia, J. Tithof, T. D. Nevins, I. Lundgaard, T. Du, D. H. Kelley, M. Nedergaard, Transcranial optical imaging reveals a pathway for optimizing the delivery of immunotherapeutics to the brain. *JCI Insight* **3**, e120922 (2018).

12. A. S. Munk, W. Wang, N. B. Bèchet, A. M. Eltanahy, A. X. Cheng, B. Sigurdsson, A. Benraiss, M. A. Mäe, B. T. Kress, D. H. Kelley, C. Betsholtz, K. Møllgård, A. Meissner, M. Nedergaard, I. Lundgaard, PDGF-B is required for development of the glymphatic system. *Cell Rep.* **26**, 2955–2969.e3 (2019).

13. L. M. Valnes, S. K. Mitusch, G. Ringstad, P. K. Eide, S. W. Funke, K.-A. Mardal, Apparent diffusion coefficient estimates based on 24 hours tracer movement support glymphatic transport in human cerebral cortex. *Sci. Rep.* **10**, 9176 (2020).

14. L. A. Ray, M. Pike, M. Simon, J. J. Iliff, J. J. Heys, Quantitative analysis of macroscopic solute transport in the murine brain. *Fluids Barriers CNS* **18**, 55 (2021).

15. B. Zapf, J. Haubner, M. Kuchta, G. Ringstad, P. K. Eide, K.-A. Mardal, Investigating molecular transport in the human brain from MRI with physics-informed neural networks. *Sci. Rep.* **12**, 15475 (2022).

16. J. Oldenburg, J. Renkewitz, M. Stiehm, K.-P. Schmitz, Contributions towards data driven deep learning methods to predict steady state fluid flow in mechanical heart valves. *Curr. Dir. Biomed. Eng.* **7**, 625–628 (2021).

17. X. Hou, P. Guo, P. Wang, P. Liu, D. D. M. Lin, H. Fan, Y. Li, Z. Wei, Z. Lin, D. Jiang, Deep-learning-enabled brain hemodynamic mapping using resting-state fMRI. *NPJ Digit. Med.* **6**, 116 (2023).

18. D. R. Rutkowski, A. Roldán-Alzate, K. M. Johnson, Enhancement of cerebrovascular 4D flow MRI velocity fields using machine learning and computational fluid dynamics simulation data. *Sci. Rep.* **11**, 10240 (2021).

19. S. Talebi, S. Gai, A. Sossin, V. Zhu, E. Tong, M. R. K. Mofrad, Deep learning for perfusion cerebral blood flow (CBF) and volume (CBV) predictions and diagnostics. *Ann. Biomed. Eng.* **52**, 1568–1575 (2024).

20. V. Ratner, Y. Gao, H. Lee, R. Elkin, M. Nedergaard, H. Benveniste, A. Tannenbaum, Cerebrospinal and interstitial fluid transport via the glymphatic pathway modeled by optimal mass transport. *Neuroimage* **152**, 530–537 (2017).

21. A. F. Frangi, J. A. Schnabel, C. Davatzikos, C. Alberola-López, G. Fichtinger, *GlymphVIS: Visualizing Glymphatic Transport Pathways Using Regularized Optimal Transport* (Springer International Publishing, 2018).

22. S. Koundal, R. Elkin, S. Nadeem, Y. Xue, S. Constantinou, S. Sanggaard, X. Liu, B. Monte, X. Feng, W. Nostrand, M. Nedergaard, H. Lee, J. Wardlaw, H. Benveniste, A. Tannenbaum, Optimal mass transport with Lagrangian workflow reveals advective and diffusion driven solute transport in the glymphatic system. *Sci. Rep.* **10**, 1–18 (2020).

23. X. Chen, X. Liu, S. Koundal, R. Elkin, X. Zhu, B. Monte, X. Feng, F. Dai, M. Pedram, H. Lee, J. Kipnis, A. Tannenbaum, W. E. Van Nostrand, H. Benveniste, Cerebral amyloid angiopathy is associated with glymphatic transport reduction and time-delayed solute drainage along the neck arteries. *Nat. Aging* **2**, 214–223 (2022).

24. S. Koundal, X. Chen, Z. Gursky, H. Lee, K. Xu, F. Liang, Z. Xie, F. Xu, H.-M. Lin, W. E. Van Nostrand, X. Gu, R. Elkin, A. Tannenbaum, H. Benveniste, Divergent brain solute clearance in rat models of cerebral amyloid angiopathy and Alzheimer's disease. *iScience* **27**, 111463 (2024).

25. V. Vinje, B. Zapf, G. Ringstad, P. K. Eide, M. E. Rognes, K.-A. Mardal, Human brain solute transport quantified by glymphatic mri-informed biophysics during sleep and sleep deprivation. *Fluids Barriers CNS* **20**, 62 (2023).

26. T. Bohr, P. G. Hjorth, S. C. Holst, S. Hrabětová, V. Kiviniemi, T. Lilius, I. Lundgaard, K.-A. Mardal, E. A. Martens, Y. Mori, U. V. Nägerl, C. Nicholson, A. Tannenbaum, J. H. Thomas, J. Tithof, H. Benveniste, J. J. Iliff, D. H. Kelley, M. Nedergaard, The glymphatic system: Current understanding and modeling. *iScience* **25**, 104987 (2022).

27. L. Ray, J. J. Iliff, J. J. Heys, Analysis of convective and diffusive transport in the brain interstitium. *Fluids Barriers CNS* **16**, 6 (2019).

28. K. E. Holter, B. Kehlet, A. Devor, T. J. Sejnowski, A. M. Dale, S. W. Omholt, O. P. Ottersen, E. A. Nagelhus, K.-A. Mardal, K. H. Pettersen, Interstitial solute transport in 3D reconstructed neuropil occurs by diffusion rather than bulk flow. *Proc. Nat. Acad. Sci. U.S.A.* **114**, 9894–9899 (2017).

29. M. Keith Sharp, R. O. Carare, B. A. Martin, Dispersion in porous media in oscillatory flow between flat plates: Applications to intrathecal, periarterial and paraarterial solute transport in the central nervous system. *Fluids Barriers CNS* **16**, 13 (2019).

30. F. Romanò, V. Suresh, P. A. Galie, J. B. Grotberg, Peristaltic flow in the glymphatic system. *Sci. Rep.* **10**, 21065 (2020).

31. R. T. Kedarasetti, P. J. Drew, F. Costanzo, Arterial vasodilation drives convective fluid flow in the brain: A poroelastic model. *Fluids Barriers CNS* **19**, 34 (2022).

32. M. M. Meerschaert, C. Tadjeran, Finite difference approximations for fractional advection–dispersion flow equations. *J. Comput. Appl. Math.* **172**, 65–77 (2004).

33. M. Raissi, P. Perdikaris, G. E. Karniadakis, Physics-informed neural networks: A deep learning framework for solving forward and inverse problems involving nonlinear partial differential equations. *J. Comput. Phys.* **378**, 686–707 (2019).

34. M. Raissi, A. Yazdani, G. E. Karniadakis, Hidden fluid mechanics: Learning velocity and pressure fields from flow visualizations. *Science* **367**, 1026–1030 (2020).

35. S. Cai, H. Li, F. Zheng, F. Kong, M. Dao, G. E. Karniadakis, S. Suresh, Artificial intelligence velocimetry and microaneurysm-on-a-chip for three-dimensional analysis of blood flow in physiology and disease. *Proc. Natl. Acad. Sci. U.S.A.* **118**, e2100697118 (2021).

36. J. D. Toscano, T. Käufer, Z. Wang, M. Maxey, C. Cierpka, G. E. Karniadakis, AIVT: Inference of turbulent thermal convection from measured 3D velocity data by physics-informed Kolmogorov-Arnold networks. *Sci. Adv.* **11**, eads5236 (2025).

37. S. Kida, R. O. Weller, E.-T. Zhang, M. J. Phillips, F. Iannotti, Anatomical pathways for lymphatic drainage of the brain and their pathological significance. *Neuropathol. Appl. Neurobiol.* **21**, 181–184 (1995).

38. H. Mansour, R. Azrak, J. J. Cook, K. J. Hornburg, Y. Qi, Y. Tian, R. W. Williams, F.-C. Yeh, L. E. White, G. A. Johnson, The duke mouse brain atlas: MRI and light sheet microscopy stereotaxic atlas of the mouse brain. *Sci. Adv.* **11**, eadq8089 (2025).

39. M. Moazen, A. Alazmani, K. Rafferty, Z.-J. Liu, J. Gustafson, M. L. Cunningham, M. J. Fagan, S. W. Herring, Intracranial pressure changes during mouse development. *J. Biomech.* **49**, 123–126 (2016).

40. E. A. Schmidt, F. Despas, A. P.-L. Traon, Z. Czosnyka, J. D. Pickard, K. Rahmouni, A. Pathak, J. M. Senard, Intracranial pressure is a determinant of sympathetic activity. *Front. Physiol.* **9**, 11 (2018).

41. G. Shen, S. Link, S. Kumar, D. M. Nusbaum, D. Y. Tse, F. Yingbin, S. M. Wu, B. J. Frankfort, Characterization of retinal ganglion cell and optic nerve phenotypes caused by sustained intracranial pressure elevation in mice. *Sci. Rep.* **8**, 2856 (2018).

42. L. Bordoni, B. Li, S. Kura, D. A. Boas, S. Sakadžić, L. Østergaard, S. Frische, E. Gutiérrez-Jiménez, Quantification of capillary perfusion in an animal model of acute intracranial hypertension. *J. Neurotrauma* **38**, 446–454 (2021).

43. K. Oshio, H. Watanabe, Y. Song, A. S. Verkman, G. T. Manley, Reduced cerebrospinal fluid production and intracranial pressure in mice lacking choroid plexus water channel Aquaporin-1. *FASEB J.* **19**, 76–78 (2005).

44. S. Feiler, B. Friedrich, K. Schöller, S. C. Thal, N. Plesnila, Standardized induction of subarachnoid hemorrhage in mice by intracranial pressure monitoring. *J. Neurosci. Methods* **190**, 164–170 (2010).

45. B. Yang, Z. Zador, A. S. Verkman, Glial cell aquaporin-4 overexpression in transgenic mice accelerates cytotoxic brain swelling. *J. Biol. Chem.* **283**, 15280–15286 (2008).

46. K. A. S. Boster, J. Tithof, D. D. Cook, J. H. Thomas, D. H. Kelley, Sensitivity analysis on a network model of glymphatic flow. *J. R. Soc. Interface* **19**, 20220257 (2022).

47. B. Lakshminarayanan, A. Pritzel, C. Blundell, Simple and scalable predictive uncertainty estimation using deep ensembles. *Adv. Neural Inf. Proces. Syst.* **30**, 6405–6416 (2017).

48. S. J. Anagnostopoulos, J. D. Toscano, N. Stergiopulos, G. E. Karniadakis, Learning in PINNs: Phase transition, total diffusion, and generalization. arXiv:2403.18494 (2024).

49. S. J. Anagnostopoulos, J. D. Toscano, N. Stergiopulos, G. E. Karniadakis, Residual-based attention in physics-informed neural networks. *Comput. Methods Appl. Mech. Eng.* **421**, 116805 (2024).

50. J. D. Toscano, L.-L. Wang, G. E. Karniadakis, KKANS: Kůrková-Kolmogorov-Arnold networks and their learning dynamics. *Neural Netw.* **191**, 107831 (2025).

51. K. Shukla, J. D. Toscano, Z. Wang, Z. Zou, G. E. Karniadakis, A comprehensive and FAIR comparison between MLP and KAN representations for differential equations and operator networks. *Comput. Methods Appl. Mech. Eng.* **431**, 117290 (2024).

52. C. Wu, J. D. Toscano, K. Shukla, Y. Chen, A. Shahmohammadi, E. Raymond, T. Toupy, N. Nazemifard, C. Papageorgiou, G. E. Karniadakis, FMEnets: Flow, material, and energy networks for non-ideal plug flow reactor design. arXiv:2505.20300 (2025).

53. J. J. Iliff, M. Wang, B. A. Yang Liao, W. P. Plog, G. A. Gundersen, H. Benveniste, G. E. Vates, R. Deane, S. A. Goldman, E. A. Nagelhus, M. Nedergaard, A paravascular pathway facilitates CSF flow through the brain parenchyma and the clearance of interstitial solutes, including amyloid β. *Sci. Transl. Med.* **4**, 147ra111 (2012).

54. J. V. George, K. J. Hornburg, A. Merrill, E. Marvin, K. Conrad, K. Welle, R. Gelein, D. Chalupa, U. Graham, G. Oberdörster, G. A. Johnson, D. A. Cory-Slechta, M. Sobolewski, Brain iron accumulation in neurodegenerative disorders: Does air pollution play a role? *Part. Fibre Toxicol.* **22**, 9 (2025).

55. R. D. Penn, A. Linninger, The physics of hydrocephalus. *Pediatr. Neurosurg.* **45**, 161–174 (2009).

56. Y. Guo, K. Quirk, D. H. Kelley, J. H. Thomas, Advection and diffusion in perivascular and extracellular spaces in the brain. *J. R. Soc. Interface* **22**, 20250010 (2025).

57. B. Bedussi, M. Almasian, J. de Vos, E. Van Bavel, E. N. Bakker, Paravascular spaces at the brain surface: Low resistance pathways for cerebrospinal fluid flow. *J. Cereb. Blood Flow Metab.* **38**, 719–726 (2018).

58. P. J. Basser, Interstitial pressure, volume, and flow during infusion into brain tissue. *Microvasc. Res.* **44**, 143–165 (1992).

59. P. J. Drew, A. Y. Shih, D. Kleinfeld, Fluctuating and sensory-induced vasodynamics in rodent cortex extend arteriole capacity. *Proc. Natl. Acad. Sci. U.S.A.* **108**, 8473–8478 (2011).

60. S. J. van Veluw, S. S. Hou, M. Calvo-Rodriguez, M. Arbel-Ornath, A. C. Snyder, M. P. Frosch, S. M. Greenberg, B. J. Bacskai, Vasomotion as a driving force for paravascular clearance in the awake mouse brain. *Neuron* **105**, 549–561.e5 (2020).

61. X. Wang, J. A. Padawer-Curry, A. R. Bice, B. Kim, Z. P. Rosenthal, J.-M. Lee, M. S. Goyal, S. L. Macauley, A. Q. Bauer, Spatiotemporal relationships between neuronal, metabolic, and hemodynamic signals in the awake and anesthetized mouse brain. *Cell Rep.* **43**, 114723 (2024).

62. G. A. Johnson, Y. Tian, D. G. Ashbrook, G. P. Cofer, J. J. Cook, J. C. Gee, A. Hall, K. Hornburg, C. C. Kaczorowski, Y. Qi, F.-C. Yeh, N. Wang, L. E. White, R. W. Williams, Merged magnetic resonance and light sheet microscopy of the whole mouse brain. *Proc. Natl. Acad. Sci. U.S.A.* **120**, e2218617120 (2023).

63. X. Han, S. Maharjan, J. Chen, Y. Zhao, Y. Qi, L. E. White, G. Allan Johnson, N. Wang, High-resolution diffusion magnetic resonance imaging and spatial-transcriptomic in developing mouse brain. *Neuroimage* **297**, 120734 (2024).

64. E. H. Stanton, N. D. Å. Persson, R. S. Gomolka, T. Lilius, B. Sigurdsson, H. Lee, A. L. R. Xavier, H. Benveniste, M. Nedergaard, Y. Mori, Mapping of csf transport using high spatiotemporal resolution dynamic contrast-enhanced mri in mice: Effect of anesthesia. *Magn. Reson. Med.* **85**, 3326–3342 (2021).

65. R. S. Gomolka, L. M. Hablitz, H. Mestre, M. Giannetto, D. Ting, N. L. Hauglund, L. Xie, W. Peng, P. M. Martinez, M. Nedergaard, Y. Mori, Loss of aquaporin-4 results in glymphatic system dysfunction via brain-wide interstitial fluid stagnation. *eLife* **12**, e82232 (2023).

66. T. D. Nevins, D. H. Kelley, Front tracking for quantifying advection-reaction-diffusion. *Chaos* **27**, 043105–043110 (2017).

67. T. D. Nevins, D. H. Kelley, Front tracking velocimetry in advection-reaction-diffusion systems. *Chaos* **28**, 043122–043111 (2018).

68. G. Ringstad, L. M. Valnes, A. M. Dale, A. H. Pripp, S.-A. S. Vatnehol, K. E. Emblem, K.-A. Mardal, P. K. Eide, Brain-wide glymphatic enhancement and clearance in humans assessed with mri. *JCI Insight* **3**, 7 (2018).

69. P. K. Eide, G. Ringstad, Functional analysis of the human perivascular subarachnoid space. *Nat. Commun.* **15**, 2001 (2024).

70. J. D. Toscano, V. Oommen, A. J. Varghese, Z. Zou, N. A. Daryakenari, W. Chenxi, G. E. Karniadakis, From pinns to pikans: Recent advances in physics-informed machine learning. arXiv:2410.13228 [cs.LG] (2024).

71. S. Wang, S. Sankaran, H. Wang, P. Perdikaris, An expert's guide to training physics-informed neural networks. arXiv:2308.08468 [cs.LG] (2023).

72. T. Salimans, D. P. Kingma, Weight normalization: A simple reparameterization to accelerate training of deep neural networks. *Adv. Neural Inf. Proces. Syst.* **29**, 901–909 (2016).

73. S. Wang, B. Li, Y. Chen, P. Perdikaris, Piratenets: Physics-informed deep learning with residual adaptive networks. *J. Mach. Learn. Res.* **25**, 19707–19757 (2024).

74. J. D. Toscano, D. T. Chen, V. Oommen, J. Darbon, G. E. Karniadakis, A variational framework for residual-based adaptivity in neural PDE solvers and operator learning. arXiv:2509.14198 [cs.LG] (2025).

75. J. Quiñonero-Candela, I. Dagan, B. Magnini, F. D'Alché-Buc, Machine Learning Challenges: Evaluating Predictive Uncertainty, Visual Object Classification, and Recognizing Textual Entailment, First Pascal Machine Learning Challenges Workshop, MLCW 2005, Southampton, UK, April 11-13, 2005, Revised Selected Papers (Springer, 2006), vol. 3944, 10.1007/11736790.

76. I. Loshchilov, F. Hutter, Decoupled weight decay regularization. arXiv:1711.05101 [cs.LG] (2017).

77. L. D. McClenny, UlissesMBraga-Neto., Self-adaptive physics-informed neural networks. *J. Comput. Phys.* **474**, 111722 (2023).

78. J. D. Toscano, D. T. Chen, G. E. Karniadakis, ATHENA: Agentic team for hierarchical evolutionary numerical algorithms. arXiv:2512.03476 [cs.LG] (2025).

79. W. Chenxi, M. Zhu, Q. Tan, Y. Kartha, L. Lu, A comprehensive study of non-adaptive and residual-based adaptive sampling for physics-informed neural networks. *Comput. Methods Appl. Mech. Eng.* **403**, 115671 (2023).

80. A. A. Linninger, M. Xenos, D. C. Zhu, M. B. R. Somayaji, S. Kondapalli, R. D. Penn, Cerebrospinal fluid flow in the normal and hydrocephalic human brain. *IEEE Trans. Biomed. Eng.* **54**, 291–302 (2007).

81. T. Koch, K.-A. Mardal, Estimation of fluid flow velocities in cortical brain tissue driven by the microvasculature. *Interface Focus* **15**, 20240042 (2025).

82. J. P. Kinney, J. Spacek, T. M. Bartol, C. L. Bajaj, K. M. Harris, T. J. Sejnowski, Extracellular sheets and tunnels modulate glutamate diffusion in hippocampal neuropil. *J. Comp. Neurol.* **521**, 448–464 (2013).

83. A. F. Psaros, X. Meng, Z. Zou, L. Guo, G. E. Karniadakis, Uncertainty quantification in scientific machine learning: Methods, metrics, and comparisons. *J. Comput. Phys.* **477**, 111902 (2023).

84. Z. Zou, X. Meng, G. E. Karniadakis, Uncertainty quantification for noisy inputs-outputs in physics-informed neural networks and neural operators arXiv:2311.11262 [cs.LG] (2023).

85. Z. Zou, X. Meng, A. F. Psaros, G. E. Karniadakis, NeuralUQ: A comprehensive library for uncertainty quantification in neural differential equations and operators. *SIAM Rev.* **66**, 161–190 (2024).
